# Synthesis and biological assessment of indole derivatives containing penta-heterocycles scaffold as novel anticancer agents towards A549 and K562 cells

**DOI:** 10.1080/14756366.2022.2163393

**Published:** 2023-01-11

**Authors:** Guanglong Zhang, Zhenhua Tang, Sili Fan, Chengpeng Li, Yan Li, Weiqin Liu, Xuesha Long, Wenjing Zhang, Yi Zhang, Zhurui Li, Zhenchao Wang, Danping Chen, Guiping Ouyang

**Affiliations:** a State Key Laboratory Breeding Base of Green Pesticide and Agricultural Bioengineering, Key Laboratory of Green Pesticide and Agricultural Bioengineering, Ministry of Education, Center for Research and Development of Fine Chemicals of Guizhou University, Guiyang, China; b College of Pharmacy, Guizhou University, Guiyang, China; c Guizhou Engineering Laboratory for Synthetic Drugs, Guizhou University, Guiyang, China

**Keywords:** Indole derivatives, penta-heterocycle, anticancer, molecular docking

## Abstract

Herein, a new series of 2-chloro-*N*-(5-(2-oxoindolin-3-yl)-4*H*-pyrazol-3-yl) acetamide derivatives containing 1,3,4*-*thiadiazole (**10a***–***i**) and 4*H*-1,2,4-triazol-4-amine (**11a***–***r**) moiety was designed, synthesised as novel anticancer agents. The antiproliferative activity values indicated that compound **10 b** stood as the most potent derivative with IC_50_ values of 12.0 nM and 10 nM against A549 and K562 cells, respectively. Mechanism investigation and docking studies of **10 b** indicated that it possessed good apoptosis characteristic and dose-dependent growth arrest of A549 and K562 cells, blocked cell cycle into G2/M phase. Interestingly, **10 b** suppressed the growth of A549 and K562 cells via modulation of EGFR and p53-MDM2 mediated pathway.

## Graphical abstract

**Figure:**
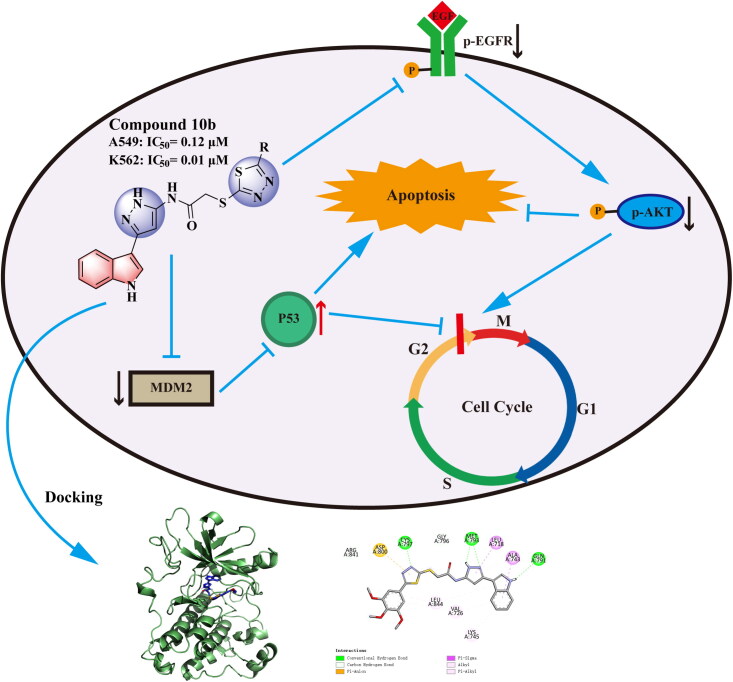

## Introduction

Cancer, a kind of pathological proliferation of abnormal cells, fast, incontrollable, and unregulated, is the second leading cause of death worldwide after cardiovascular disease^1^^,^^2^. Worldwide, the cancer incidence, an estimated 19.3 million new cancer cases in 2020, compared to 2018, an estimated 18.1 million, increased by 6.2%. Lung cancer is the most commonly diagnosed cancer (11.6% of the total cases), closely followed by female breast cancer (11.6%), prostate cancer (7.1%), and colorectal cancer (6.1%) for incidence in 2018^3^^,^^4^. Lung cancer and leukaemia remain major public health concern among current all diseases due to the toxicity and side effects of the available commercially synthesised drugs^5^^,^^6^. Currently, chemotherapy, surgical resection, and radio therapy are the main means of cancer treatment, while chemotherapy plays an indispensable role in current clinical treatment. However, due to poor selectivity, drug resistance and serious side effects of chemotherapy, its clinical application is limited. Therefore, it is of great significance to find, design and synthesise novel and highly effective targeted small molecule antitumor drugs for cancer treatment^7–9^.

**Figure 1.**
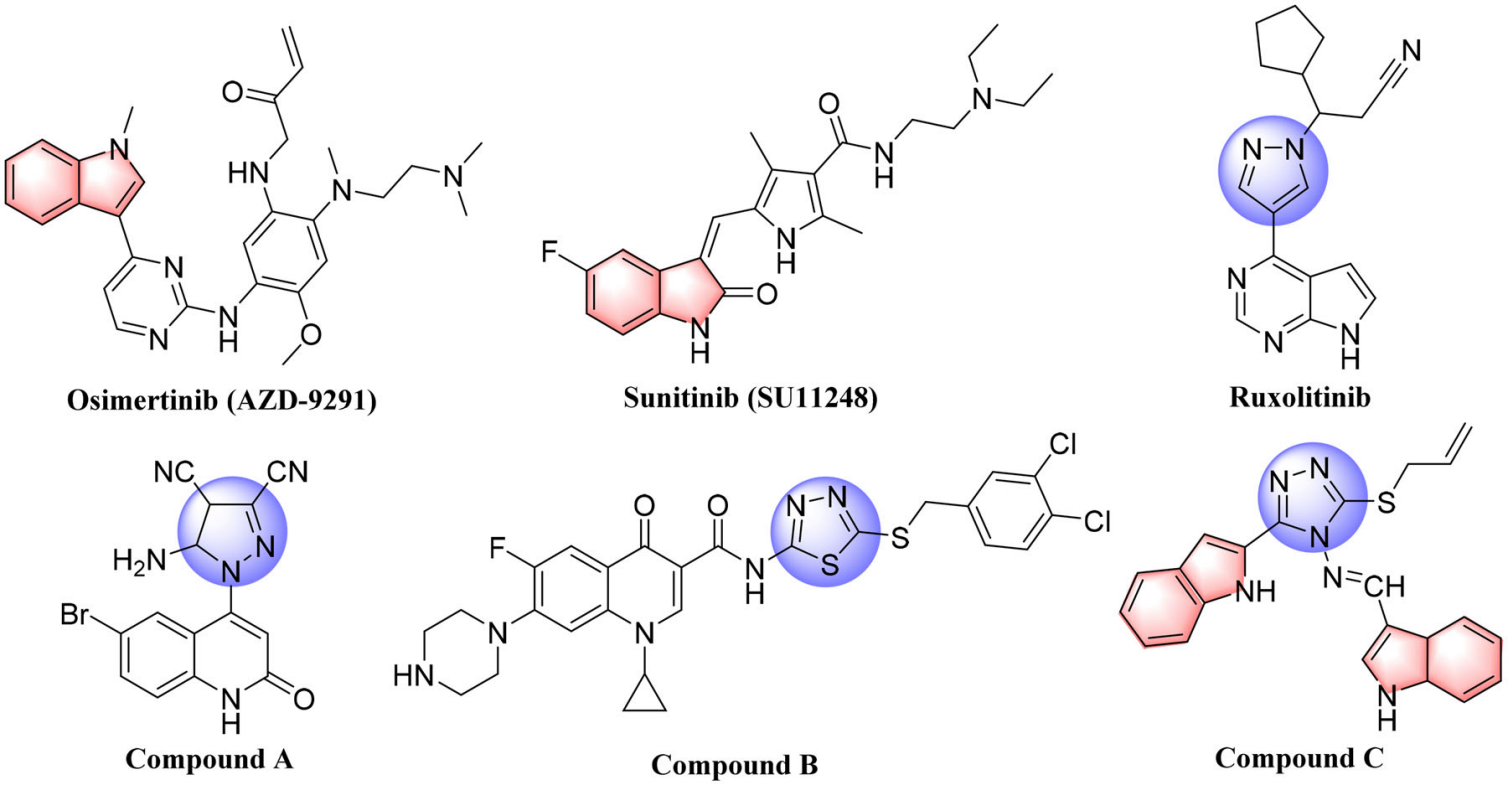
Structures of Osimertinib (AZD-9291), Sunitinib (SU11248), approved anticancer drugs, and some previously reported penta-heterocycles scaffold as antiproliferative agents (**Compound A**, **B** and **C**).

In recent studies, the development of novel scaffolds focussed on the mutant-selective EGFR-TKIs against T790M and C797S resistance, which was proven as an effective approach^10^^,^^11^. Indole core plays an important role in drug discovery, which has a wide range of biological activities, especially in the development of anticancer agents, such as Vinblastine (VLB), Vincristine (VCR), Osimertinib (AZD-9291), and Sunitinib (SU11248) (Figure 1)^12–15^. Penta-heterocycles framework is an exceptionally adaptable drug-like format that is being utilised broadly in the structure of cancer treatments and cellular apoptosis such as pyrazoles, which were reported in a variety of potent anti-cancer active agents as listed on Ruxolitinib^16^ and Compound **A**^17^ (Figure 1). Furthermore, 1,3,4-thiadiazoles such as Compound **B**^18^ was reported as highly anti-cancer agent by inducing apoptosis significantly in MCF-7 cell (Figure 1). Substituted 1,2,4-triazazole (Compound **C**)^19^ also displayed remarkable epidermal growth factor receptor (EGFR) inhibition activity with IC_50_ value of 19.8 nM (Figure 1).

**Figure 2.**
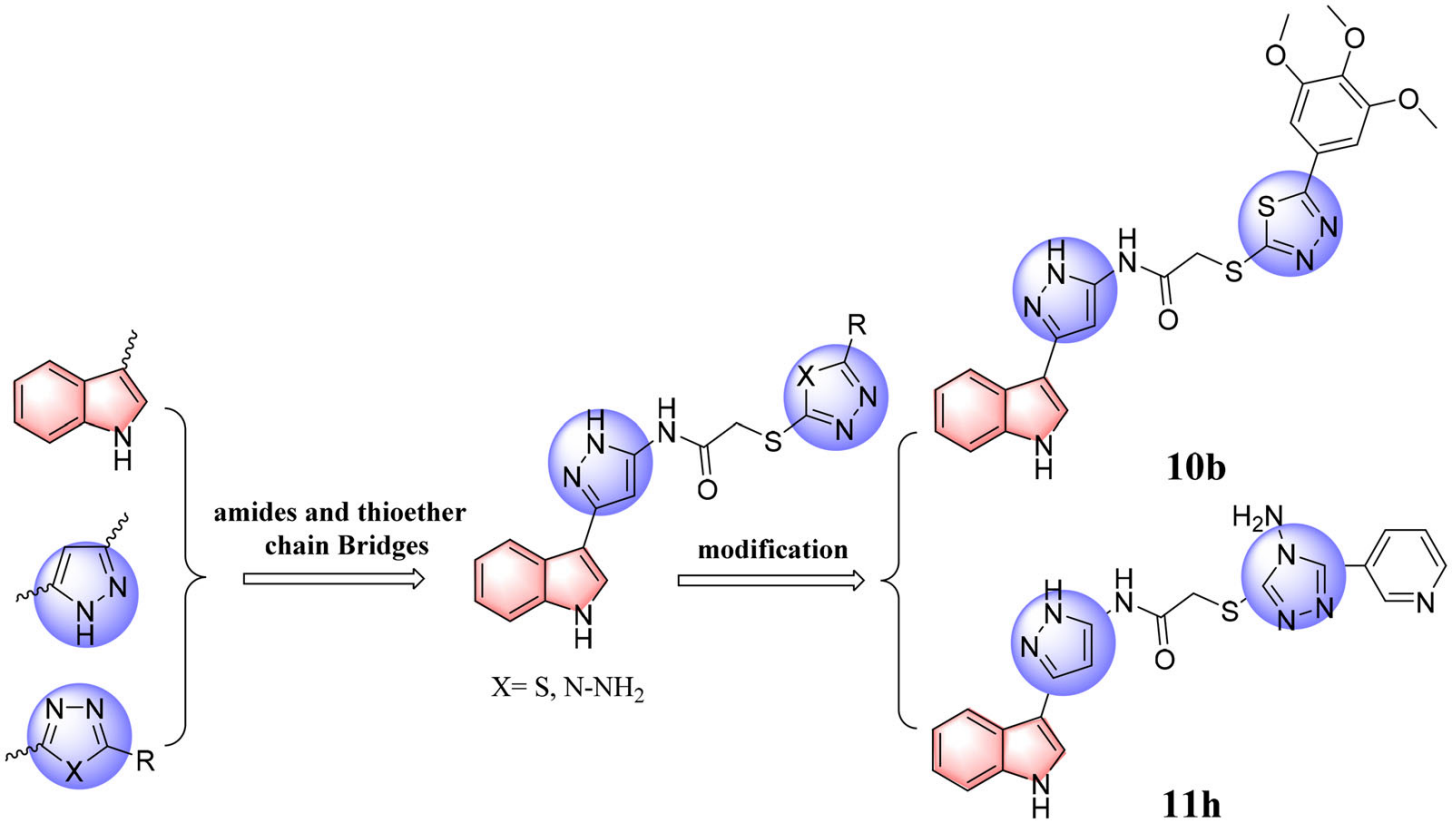
The design strategy of the target compounds in this study, and the resulting most active antitumor candidates (**10 b** and **11 h**).

EGFR mutation and overexpression are closely related to tumorigenesis and development of non-small-cell lung cancer (NSCLC), which makes EGFR an important target for the design and development of antitumor agents^20–22^. MDM2 directly binds to and forms a complex with p53, inhibiting p53 transactivation^23^. Several different therapeutic approaches have been attempted with the goal of restoring p53 function^24–27^. Among these, targeting the MDM2-p53 interaction by small molecules for the reactivation of p53 has emerged as a promising approach for the treatment of cancers that retain wild-type p53^28–30^. Our previous work explored strategies for designing and synthesising a series of heterocycle antitumor inhibitors^31–34^. Based on structure guided molecular hybridisation, we designed and synthesised a series of novel indole derivatives in this research containing 1,3,4-thiadiazoles (**10a*–*i**) and 4*H*-1,2,4-triazol-4-amines (**11a*–*r**), expecting to discover compounds with more powerful activity. In particularly, we found the resulting most active antitumor candidates (**10 b** and **11 h**) (Figure 2), as well as the inhibition mechanisms of the said compounds by apoptosis, cell cycle, cell migration, EGFR and p53-MDM2 enzyme inhibition analysis, and docking studies, wherein it proved to be a highly potent antitumor agents in the future.

Reagents and conditions: **a**: Cyanoacetic acid, Ac_2_O, 40 °C, 4 h; **b**: Hydrazine hydrate (80%), EtOH, reflux; **c**: Chloroacetyl chloride, 1,4-dioxane, rt; **d**: Hydrazine hydrate (80%), EtOH, reflux; **e**: KOH/CS_2_, EtOH, reflux; **f**: (1) KOH/CS_2_, EtOH, reflux; (2) HCl, pH4-5; **g**: (1) Hydrazine hydrate (80%), reflux; (2) HCl, pH2-3; **h**: intermediate **4**, KOH, EtOH, rt.

## Results and discussion

### Chemistry

**Scheme 1:**
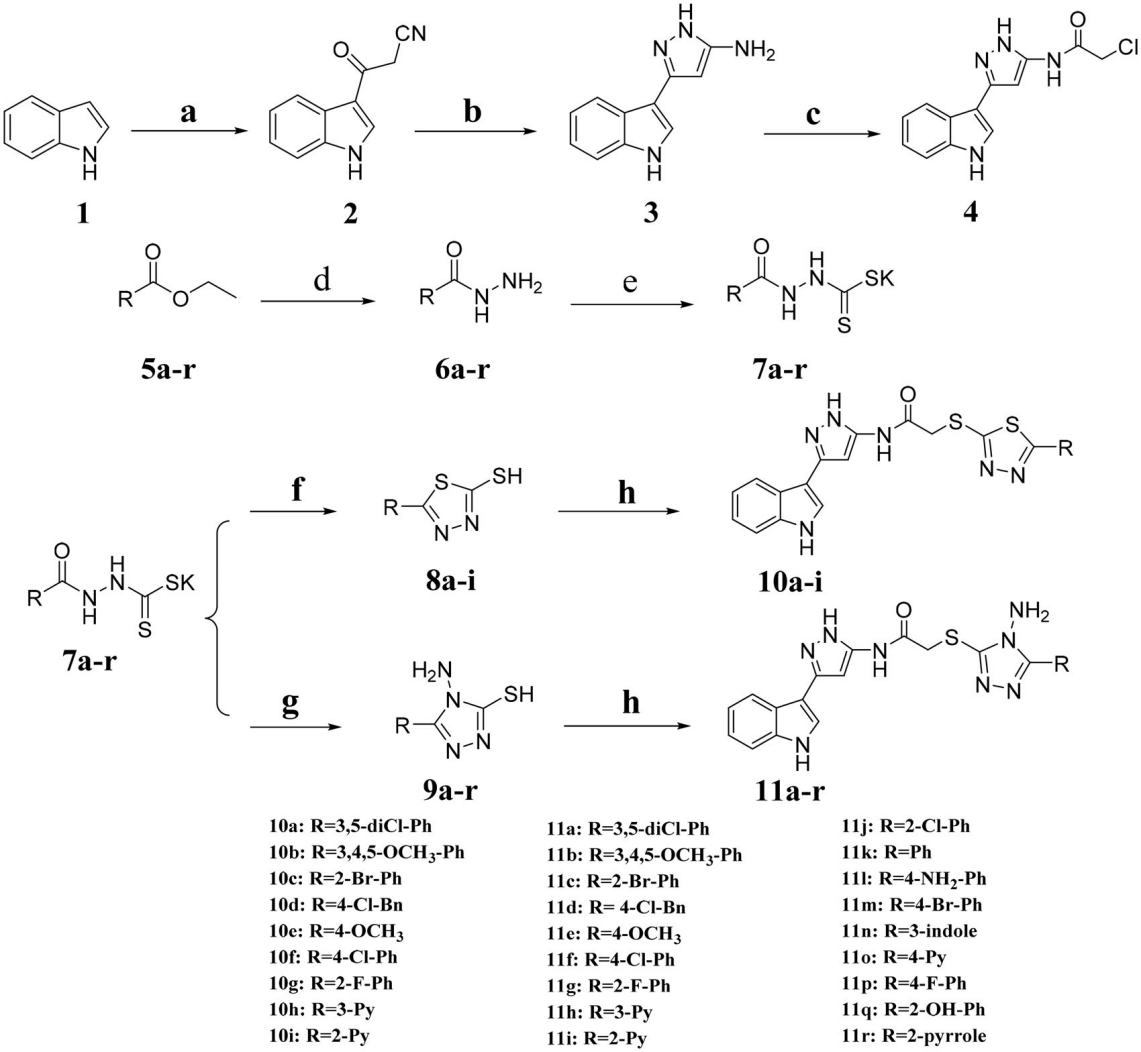
Synthetic route for the title compounds **10a*–*i** and **11a*–*r**.

The general route for the synthesis of title compounds **10a*–*i** and **11a*–*r** was illustrated in Scheme 1. Compound **2** was synthesised through indole as starting material added to cyanoacetic acid and acetic anhydride. Then, cyclisation reaction was carried out to obtain compound **3**. The step of amino acylation was operated subsequently with chloroacetyl chloride to give intermediate **4**. The 1,3,4-thiadiazoles (**8a*–*i**) and 1,2,4-triazoles (**9a–r**) were prepared according to a series of reactions of esterification, hydrazinolysis, salt formation and cyclisation. Target compounds were synthesised by binding intermediate **4** with 1,3,4-thiadiazoles (**8a–i**) and 1,2,4-triazoles (**9a–r**) using potassium hydroxide as a catalyst at room temperature. All the target derivatives were fully characterised by ^1^H NMR, ^13^C NMR and HR-MS.

### In vitro antiproliferative activities

To probe the *in vitro* cytotoxic impact of these synthesised indole derivatives (**10a*–*i** and **11a*–*r**), four human cancer cell lines, human non-small cell lung cancer cells (A549), human prostate cancer cells (PC-3), human liver cancer cells (HepG2) and human chronic myeloid leukaemia cells (K562) were selected. The results expressed as IC_50_ (µM) were summarised in Table 1. The majority of the tested compounds exhibited moderate to potent antiproliferative activity, especially the series of compounds **10a*–*i**. Remarkably, analogues **10a**, **10 b**, **10d**, **10e**, **10f**, **11 h**, **11 l** exhibited potent antitumor efficacies against K562 (IC_50_ = 0.06*–*0.99 µM), while **10a**, **10 b**, **10e** were found to be the most active compounds with the IC_50_ values of 0.12*–*0.83 µM against A549. Interestingly, **10 b** was stood as the most potent derivative with IC_50_ values of 0.12 µM, 0.85 µM, 0.12 µM, 0.21 µM, 0.01 µM, respectively, against A549, PC-3, HepG2 and K562. Clearly, **10 b** showed 82 folds and 1225 times superior potency to the reference drug Gefitinib against A549, and 5-Fluorouracil (5-Fu) against K562. On the other hand, except for compounds **11 h**, **11i** and **11 l** in the series of compounds **11a*–*r**, the rest of the compounds displayed weak to moderate anti-proliferative activities against the tested cell lines. Amazingly, compound **11 h** (IC_50_ = 0.06 µM) had excellent anticancer activity against the K562 cell line. Next, we found that compound 1,3,4-thiadiazoles displayed dramatically potent anticancer activity than 1,2,4-triazoles against four cancer cell lines. However, after introducing an electron-withdrawing group, bromine and fluorine atoms to the ortho-position in the benzene group of 1,3,4-thiadiazole derivatives, compound **10 c** and **10 g** only kept a quite weak antiproliferative activity.

**Table 1.** Antiproliferative activity of the synthesised compounds **10a*–*i** and **11a–r** ^a^.

Compound	*In vitro* cytotoxicity IC_50_^b^ (µM)				
	A549	PC-3	HepG2	K562	*HEK293*
**10 a**	0.433 ± 0.10	0.141 ± 0.01	4.45 ± 0.42	0.0527 ± 0.02	1.33 ± 0.07
**10 b**	0.124 ± 0.05	0.853 ± 0.72	0.210 ± 0.07	0.0132 ± 0.01	1.12 ± 1.39
**10 c**	18.8 ± 2.92	31.9 ± 18.01	61.3 ± 17.03	33.7 ± 3.54	63.7 ± 6.51
**10 d**	1.28 ± 0.33	1.60 ± 0.44	0.732 ± 0.15	0.184 ± 0.02	0.74 ± 1.25
**10 e**	0.834 ± 0.09	0.852 ± 0.43	0.801 ± 0.09	0.193 ± 0.09	0.89 ± 2.32
**10 f**	4.33 ± 0.30	8.86 ± 7.04	3.34 ± 0.60	0.991 ± 0.52	2.56 ± 0.90
**10 g**	14.2 ± 1.61	2.98 ± 0.28	11.8 ± 2.02	3.32 ± 0.09	10.1 ± 2.20
**10 h**	7.96 ± 1.39	16.7 ± 4.86	5.58 ± 2.69	6.93 ± 1.63	21.9 ± 1.31
**10 i**	12.0 ± 1.46	24.1 ± 5.07	8.37 ± 2.29	12.5 ± 2.46	26.3 ± 1.63
**11a**	27.3 ± 3.47	7.66 ± 1.50	40.3 ± 7.83	3.64 ± 0.08	16.8 ± 5.56
**11 b**	29.8 ± 4.05	5.73 ± 1.69	10.6 ± 1.42	3.09 ± 0.41	20.2 ± 4.08
**11 c**	67.7 ± 1.35	60.8 ± 4.73	74.7 ± 13.41	43.4 ± 1.96	75.4 ± 2.35
**11 d**	4.79 ± 0.21	8.42 ± 0.56	49.6 ± 10.19	3.51 ± 1.05	11.6 ± 0.87
**11 e**	21.4 ± 4.46	11.7 ± 1.64	10.4 ± 2.86	4.32 ± 0.53	4.94 ± 0.75
**11 f**	11.8 ± 3.00	19.1 ± 8.43	9.04 ± 0.93	32.3 ± 4.05	18.1 ± 3.14
**11 g**	10.1 ± 2.37	6.52 ± 1.85	9.65 ± 5.87	3.78 ± 0.54	5.05 ± 1.17
**11 h**	1.15 ± 0.26	3.16 ± 0.23	1.23 ± 0.06	0.0639 ± 0.02	1.79 ± 0.63
**11 i**	2.97 ± 0.83	2.77 ± 0.91	2.50 ± 1.44	1.26 ± 0.24	1.45 ± 0.22
**11 j**	56.8 ± 5.27	34.4 ± 2.57	50.6 ± 4.69	42.4 ± 3.60	63.0 ± 0.87
**11 k**	23.9 ± 3.08	25.4 ± 2.77	73.1 ± 8.07	39.7 ± 7.96	23.6 ± 4.14
**11 l**	1.73 ± 0.30	2.88 ± 0.10	1.37 ± 0.57	0.363 ± 0.10	1.38 ± 1.17
**11 m**	5.81 ± 0.69	21.3 ± 2.75	10.7 ± 5.21	2.57 ± 0.43	5.81 ± 0.88
**11 n**	31.3 ± 3.29	10.4 ± 0.46	2.76 ± 1.74	4.38 ± 0.45	5.02 ± 1.29
**11 o**	30.3 ± 7.00	38.3 ± 1.75	33.4 ± 7.97	66.0 ± 3.48	21.8 ± 1.76
**11 p**	93.9 ± 7.47	33.3 ± 5.94	19.3 ± 6.15	33.2 ± 4.95	28.9 ± 0.52
**11 q**	4.14 ± 1.71	2.89 ± 0.15	8.23 ± 7.58	2.34 ± 0.34	2.66 ± 1.29
**11 r**	2.72 ± 0.26	2.73 ± 1.05	1.75 ± 0.07	1.84 ± 0.66	1.53 ± 0.93
**5-Fluorouracil**	3.46 ± 0.45	10.9 ± 3.40	15.0 ± 3.87	12.3 ± 2.50	3.62 ± 0.25

^a^The antiproliferation activities were determined by MTT assay for 48 h. The data represented the mean of triplicate determination.

^b^IC_50_ values are indicated as the mean ± SD of at least three independent experiments.

The results of CC_50_ against normal cell line (HEK293) indicated these synthesised compounds were also moderately cytotoxic to normal cells, which were not currently the most promising candidate drugs. However, by calculating the corresponding relative selectivity indexes of compounds **10 b** and **11 h** in A549 and K562 cell lines, the results in Table 2 showed that the possibility of drug development was still optimistic. The follow-up study for us is to optimise the structure of the compound to reduce the toxicity to normal cells

**Table 2.** The selectivity index for the compounds **10 b a**nd **11 h**.

Compound	The selectivity index values (SI) ^a^				
	A549	K562	HEK293	SI (A549)	SI (K562)
**10 b**	0.124 ± 0.05	0.0132 ± 0.01	1.12 ± 1.39	9.03	84.8
**11h**	1.15 ± 0.26	0.0639 ± 0.02	1.79 ± 0.63	1.56	28.0

^a^The selectivity index values for the compounds were calculated as the ratio of the CC_50_/IC_50._

### Apoptosis analysis

Concerning the antitumor activity of derivative **10 b**, we first examined whether the inhibitory effects of **10 b** on A549 and K562 cell lines were due to apoptosis by DAPI staining and apoptosis morphological analysis. After 48 h incubation with **10 b** at 1, 2, and 4 µM in DAPI staining, analogue **10 b** induced apoptosis as shown by DAPI staining of micronuclei of proliferating A549 cells. As the nuclear membrane was ruptured, **10 b** induced significant dense blue staining and apoptotic bodies formation, thereby indicating that **10 b** had apoptotic activity against A549 cells.

**Figure 3.**
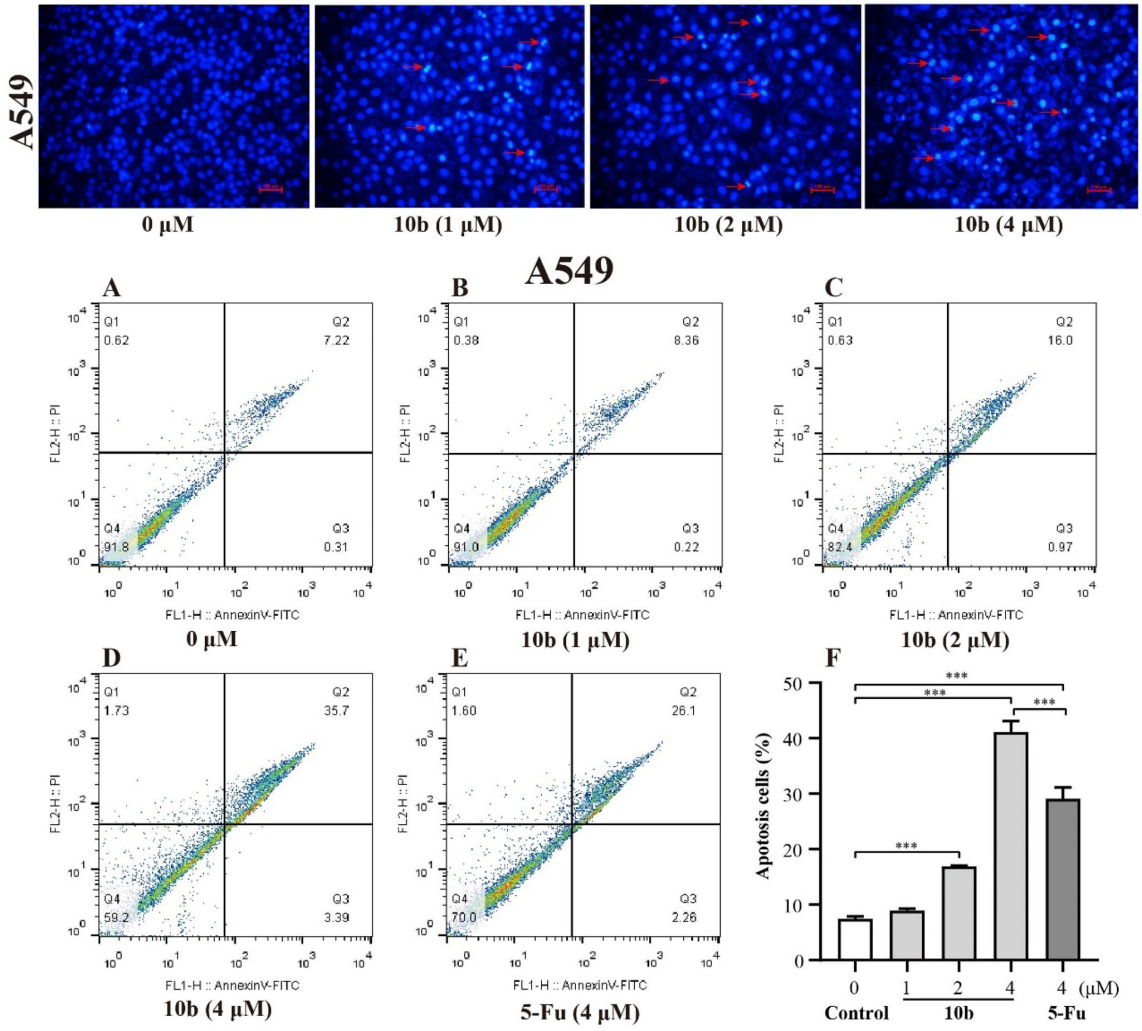
The fluorescence microscopy images of A549 cells after DAPI staining. A549 cells treated with compound **10 b** at 1, 2 and 4 μM for 48 h. Arrows indicated signs of nuclear shrinkage and chromatin condensation, the scar bar is 100 μm. And the apoptosis analysis through annexin V-FITC/PI double staining and following flow cytometry for the A549 cells treated with compound **10 b** at 1, 2 and 4 μM for 48 h. DMSO was the negative control, as 5-Fu was the reference drug. (A) Control, (B) **10 b** (1 μM), (C) **10 b** (2 μM), (D) **10 b** (4 μM) and (E) 5-Fu (4 μM). (F) The percentage of apoptosis cells was quantified in the segments of the bar chart. Three individual experiments were performed for each group. Data are expressed as the mean ± SD of three independent experiments. ***p < 0.005.

**Figure 4.**
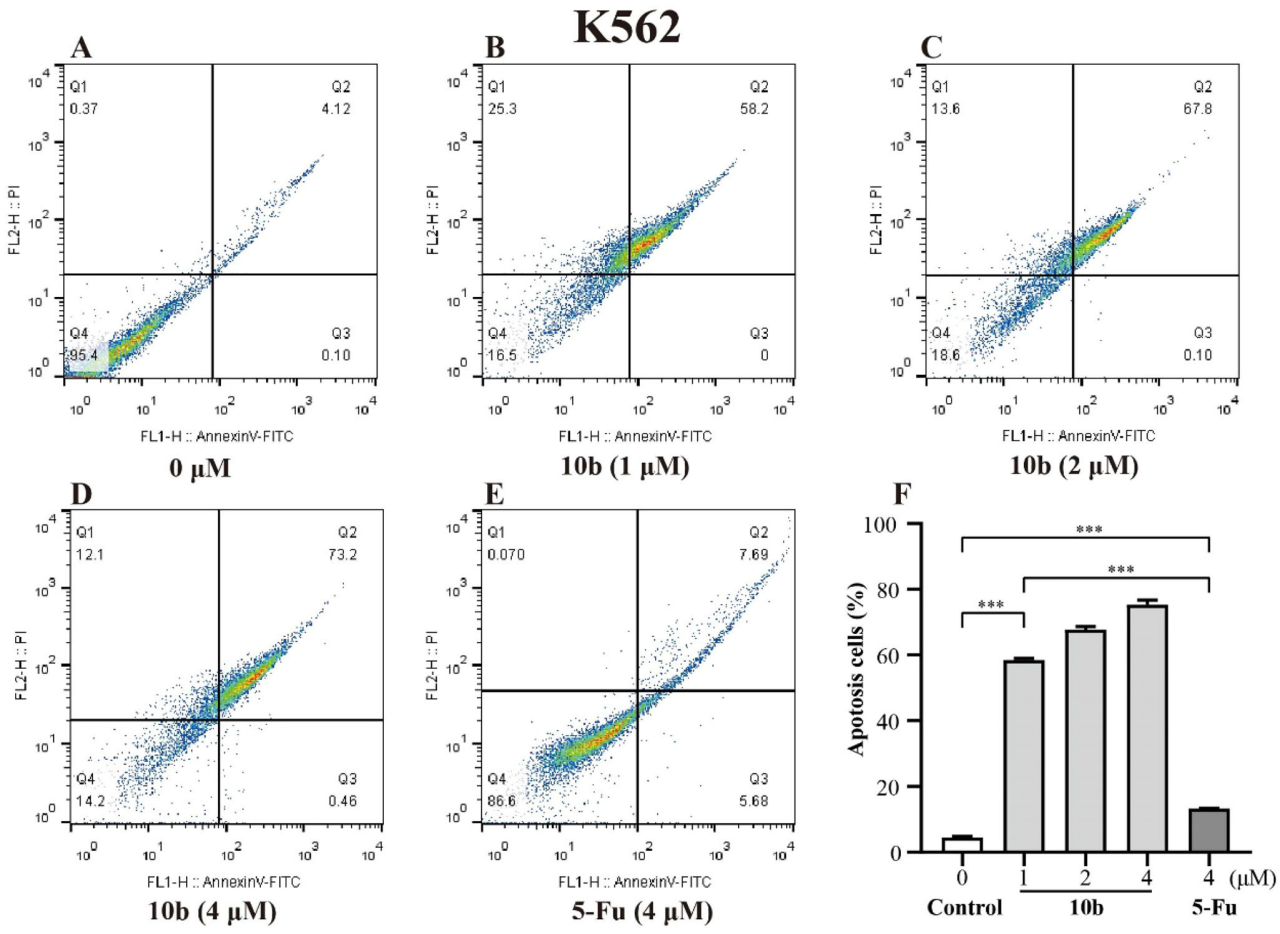
Apoptosis analysis through annexin V-FITC/PI double staining and following flow cytometry for the K562 cells treated with compound **10 b** at 1, 2 and 4 μM for 48 h. DMSO was the negative control, as 5-Fu was the reference drug. (A) Control, (B) **10 b** (1 μM), (C) **10 b** (2 μM), (D) **10 b** (4 μM) and (E) 5-Fu (4 μM). (F) The percentage of apoptosis cells was quantified in the segments of the bar chart. Three individual experiments were performed for each group. Data are expressed as the mean ± SD of three independent experiments. ***p < 0.005.

Meanwhile, the detailed cytotoxic effects of A549 and K562 cells were also performed through annexin V-FITC/PI double staining and quantified by flow cytometry for apoptotic analysis method. As shown in Figure 3, we tested apoptosis caused by **10 b** and compared it with 5-Fu in A549 and K562 cells. After 48 h incubation, the results showed that the percentage of the apoptotic cells of A549 and K562 treated with **10 b** mainly lead to late apoptotic. Specifically, the numbers of late apoptotic cells of A549 were up to 8.36%, 16.0% and 35.7% at 1, 2 and 4 µM in **10 b** treatment, respectively, whereas the total numbers of apoptotic cells were only 28.3% at 4 µM 5-Fu treatment (Figure 3). There were special concerns associated with the percentage of late apoptotic cells of K562 were rapidly up to 58.2%, 67.8% and 73.2% in a dose dependent manner at 1, 2 and 4 µM **10 b** treatment, respectively, whereas that the total numbers of apoptotic cells were only 13.4% at 4 µM 5-Fu treatment (Figure 4). Indeed, cell apoptosis was increased for **10 b** compared with control 5-Fu both in A549 and K562 cells.

### Cell cycle analysis

**Figure 5.**
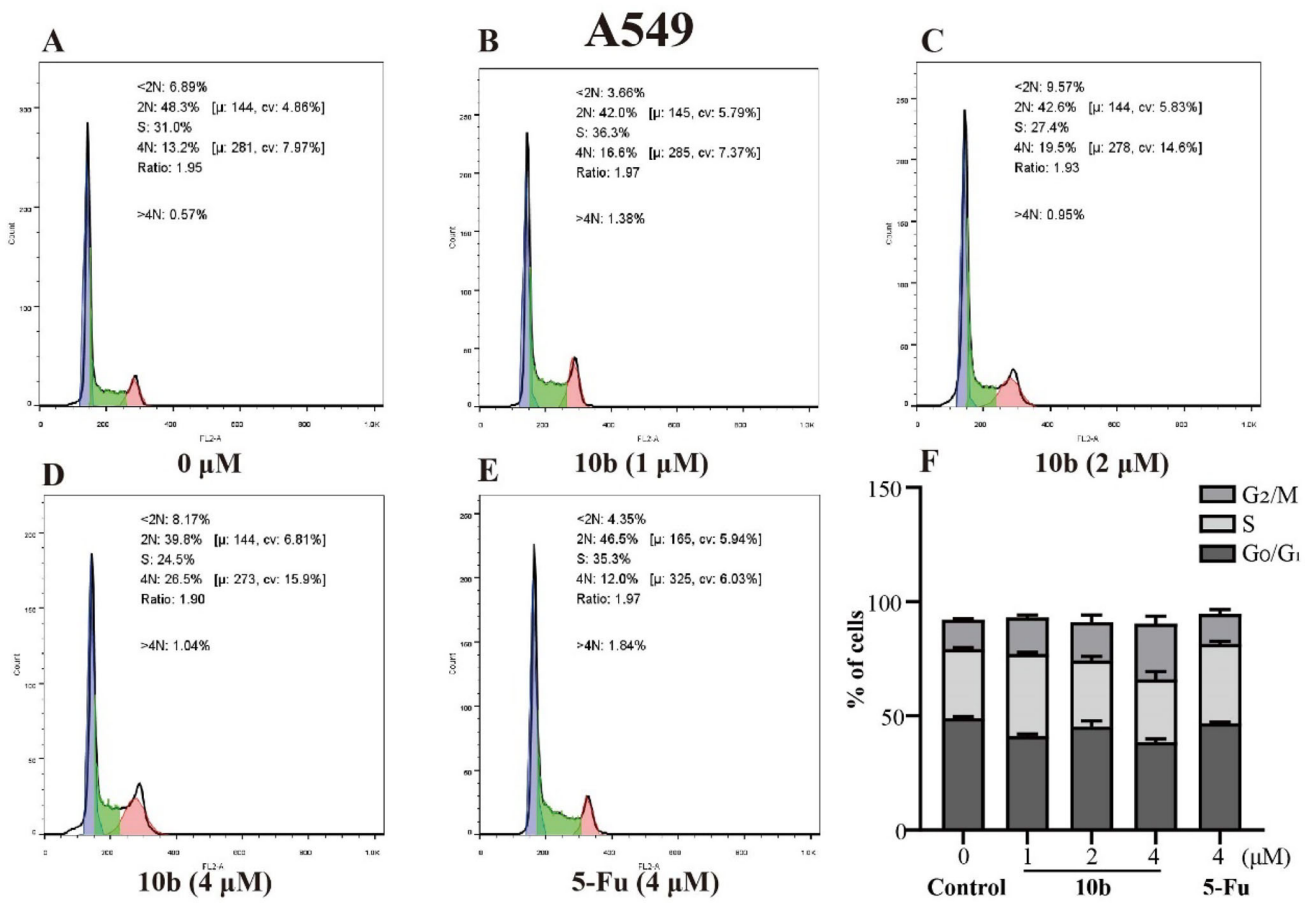
Cell cycle analysis through PI staining and following flow cytometry for the A549 cells treated with compound **10 b** at 1, 2 and 4 μM for 24 h. DMSO was the negative control, as 5-Fu was the reference drug. (A) Control, (B) **10 b** (1 μM), (C) **10 b** (2 μM), (D) **10 b** (4 μM) and (E) 5-Fu (4 μM). (F) The proportion of the cell cycle was quantified in the segments of the bar chart. Three individual experiments were performed for each group. Data are expressed as the mean ± SD of three independent experiments. ***p < 0.005.

**Figure 6.**
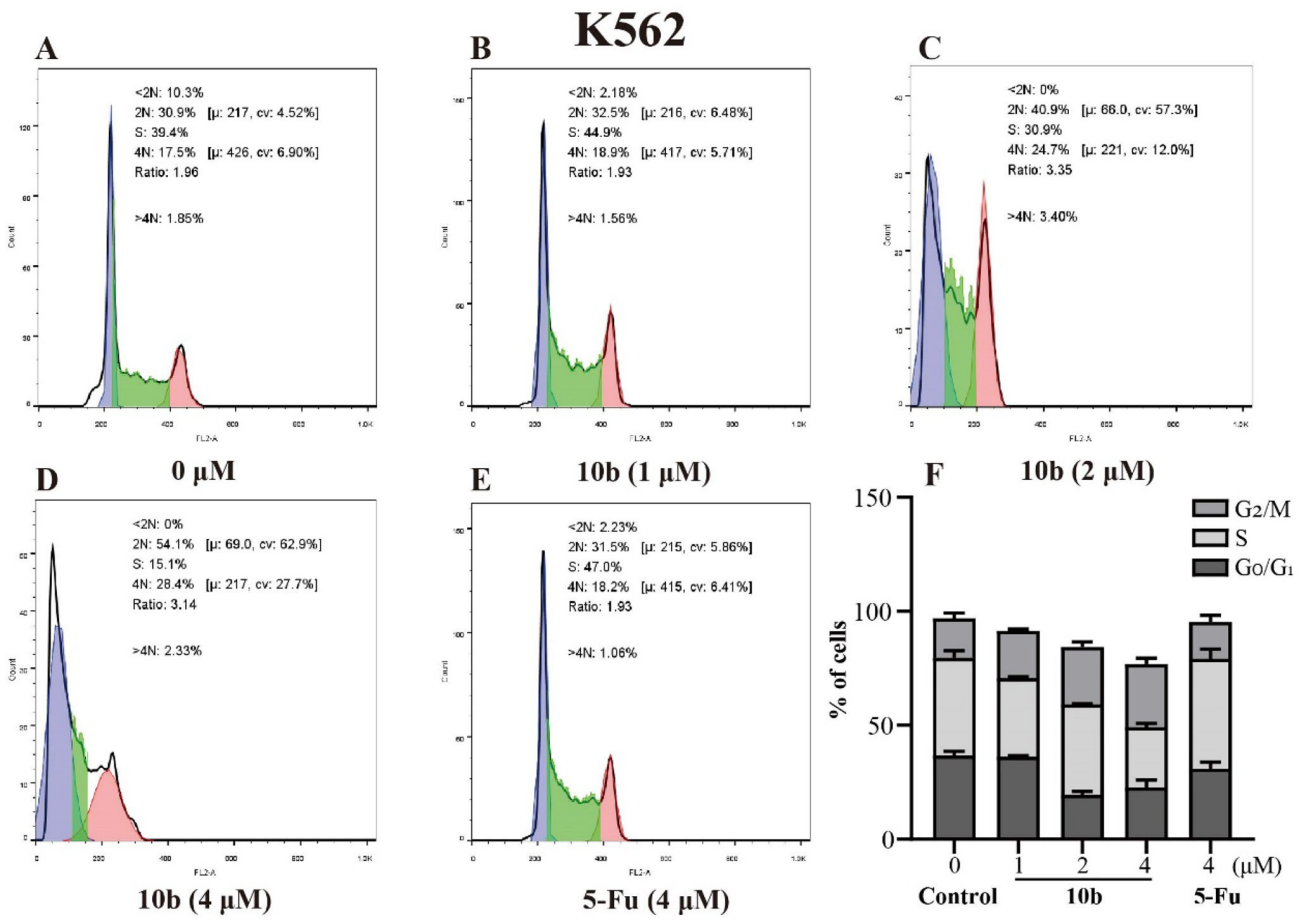
Cell cycle analysis through PI staining and following flow cytometry for the K562 cells treated with compound **10 b** at 1, 2 and 4 μM for 24 h. DMSO was the negative control, as 5-Fu was the reference drug. (A) Control, (B) **10 b** (1 μM), (C) **10 b** (2 μM), (D) **10 b** (4 μM) and (E) 5-Fu (4 μM). (F) The proportion of the cell cycle was quantified in the segments of the bar chart. Three individual experiments were performed for each group. Data are expressed as the mean ± SD of three independent experiments. ***p < 0.005.

Cell cycle arrest was an important indication for inhibition of proliferation^35^; therefore, we evaluated the effect of the most promising compound **10 b** on the cell cycle of A549 and K562 cells using the flow cytometry assay. Thus, the influence of **10 b** on the cell cycle progression was investigated, with 48 h following treatment at 1, 2, 4 µM, interpreted in Figures 5 and 6. When the A549 cell line was exposed to **10 b** at 4 µM for 48 h, the DNA contents of the live population were 49.03%, 30.33% and 12.77% for untreated cells, 38.60%, 27.47% and 24.33% for **10 b**-treated cells, and 46.87%, 34.63%, and 13.13% for 5-Fu-treated cells (4 µM), respectively, for G0/G1, S, and G2/M phase. Indeed, treatment with **10 b** (4 µM) resulted in a 1.85*-*fold than 5-Fu treatment, respectively increased in the fraction of cells arrested at G2/M. Similar results were also observed for the K562 cell line. When the K562 cell line was exposed to **10 b** at 4 µM for 48 h, the DNA contents of the live population were 36.70%, 42.80%, and 17.43% for untreated cells, 22.73%, 26.47%, and 27.60% for **10 b**-treated cells, and 30.93%, 48.27%, and 16.07% for 5-Fu-treated cells (4 µM), respectively, for G0/G1, S, and G2/M phase. Meanwhile, treatment with **10 b** (4 µM) resulted in a 1.72-fold than 5-Fu treatment, respectively increased in the fraction of cells arrested at G2/M. Taken together, these results suggested that **10 b** delayed cell cycle progression by arresting cells in the G2/M phase of the cell cycle in a dose-dependent manner, regarding A549 and K562 cell growth.

### Cell migration assay

**Figure 7.**
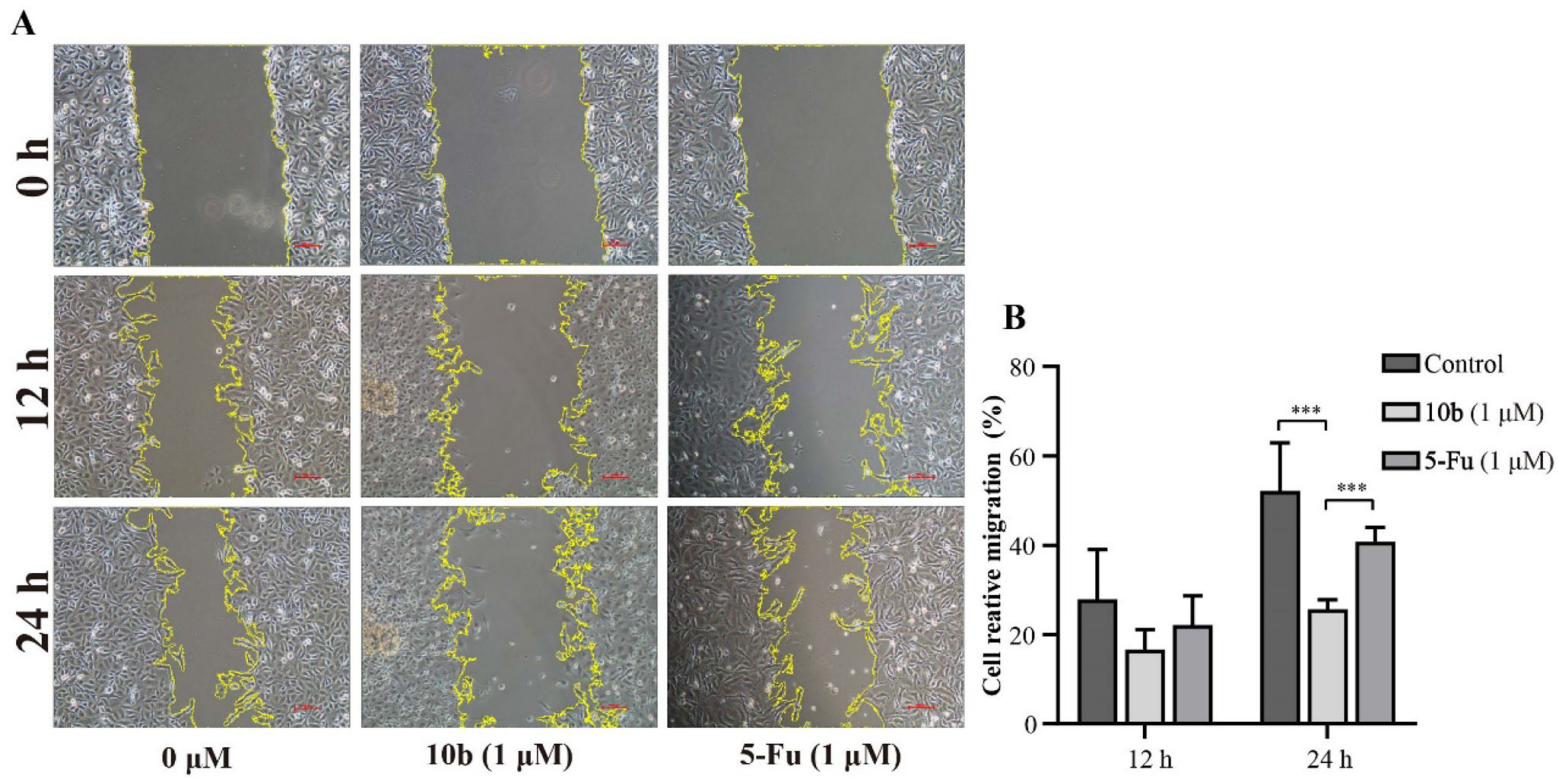
Cell migration and invasion assay carried out in A549 after 12 and 24 h of 1 μM **10 b** or 5-Fu treatment. (A) Representative images of wound healing migration assay; (B) Semi-quantitative analysis of migration cells at non-covered area. The scar bar is 100 μm. Three individual experiments were performed for each group. Data are expressed as the mean ± SD of three independent experiments. ***p < 0.005.

Analysis of cell migration *in vitro* is a useful assay to quantify alterations in cell migratory capacity in response to experimental manipulations^36^. Meanwhile, cell migration also is an important malignant behaviour and contributes to metastasis, a major cause of cancer-related death^37^. After incubation with compound **10 b** of 1 µM for a few hours, the scratched areas of experimental groups were significantly larger than the control groups, indicating that compound **10 b** could inhibit the migration of A549 cells (Figure 7).

### Western blot assay

**Figure 8.**
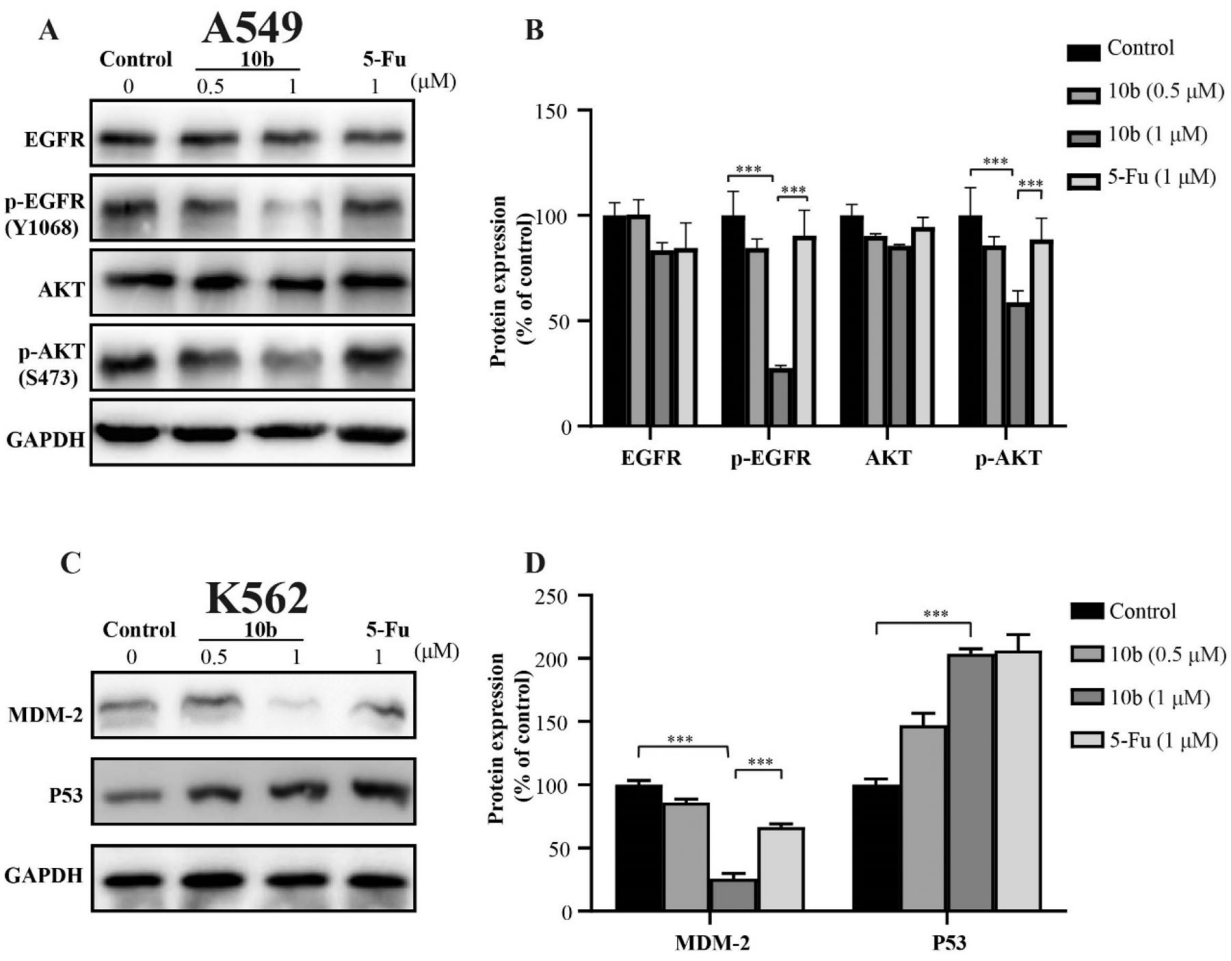
Western blot analysis of proteins obtained from cells treated with compound **10 b** at 0.5 and 1 μM for 48 h. DMSO was the negative control, as 5-Fu was the reference drug. (A) Western blotting analysis of EGFR, p-EGFR, Akt and p-Akt levels in A549. (B) Average fold change of EGFR, p-EGFR, Akt and p-Akt with respect to the housekeeping gene GAPDH in different treatment groups following densitometric analysis. (C) Western blotting analysis of MDM2 and p53 levels in K562. (D) Average fold change of MDM2 and p53 with respect to the housekeeping gene GAPDH in different treatment groups following densitometric analysis. Three individual experiments were performed for each group. Data are expressed as the mean ± SD of three independent experiments. ***p < 0.005.

To better understand the mechanisms involved in compound **10 b**-mediated antitumor activity, we further evaluated the effect of **10 b** on expression level and phosphorylation of EGFR and AKT in A549 cells using western blot assay^38^^,^^39^. Because MDM2 is a primary cellular inhibitor of p53, it may be effective in the treatment of human cancer through retaining wild-type p53 by reactivating the p53 tumour suppressor function. Thus, the effect of **10 b** on p53 in K562 cells was further investigated^40^. These results indicated that the p-EGFR and p-Akt proteins in A549 cells was downregulated in a dose dependently manner after 48 h treatment of **10 b**. Moreover, after treated with compound **10 b** at 0.5 µM in K562 cells, p53 protein was upregulated in a certain extent. Obviously, the expression of MDM2, as the negative regulator of p53 protein, was significantly downregulated. These data suggested that compound **10 b** could inhibit the proliferation of A549 cells through EGFR-mediated pathway and regulate MDM2-p53 interaction to inhibit the K562 cells (Figure 8).

### Docking studies

**Figure 9.**
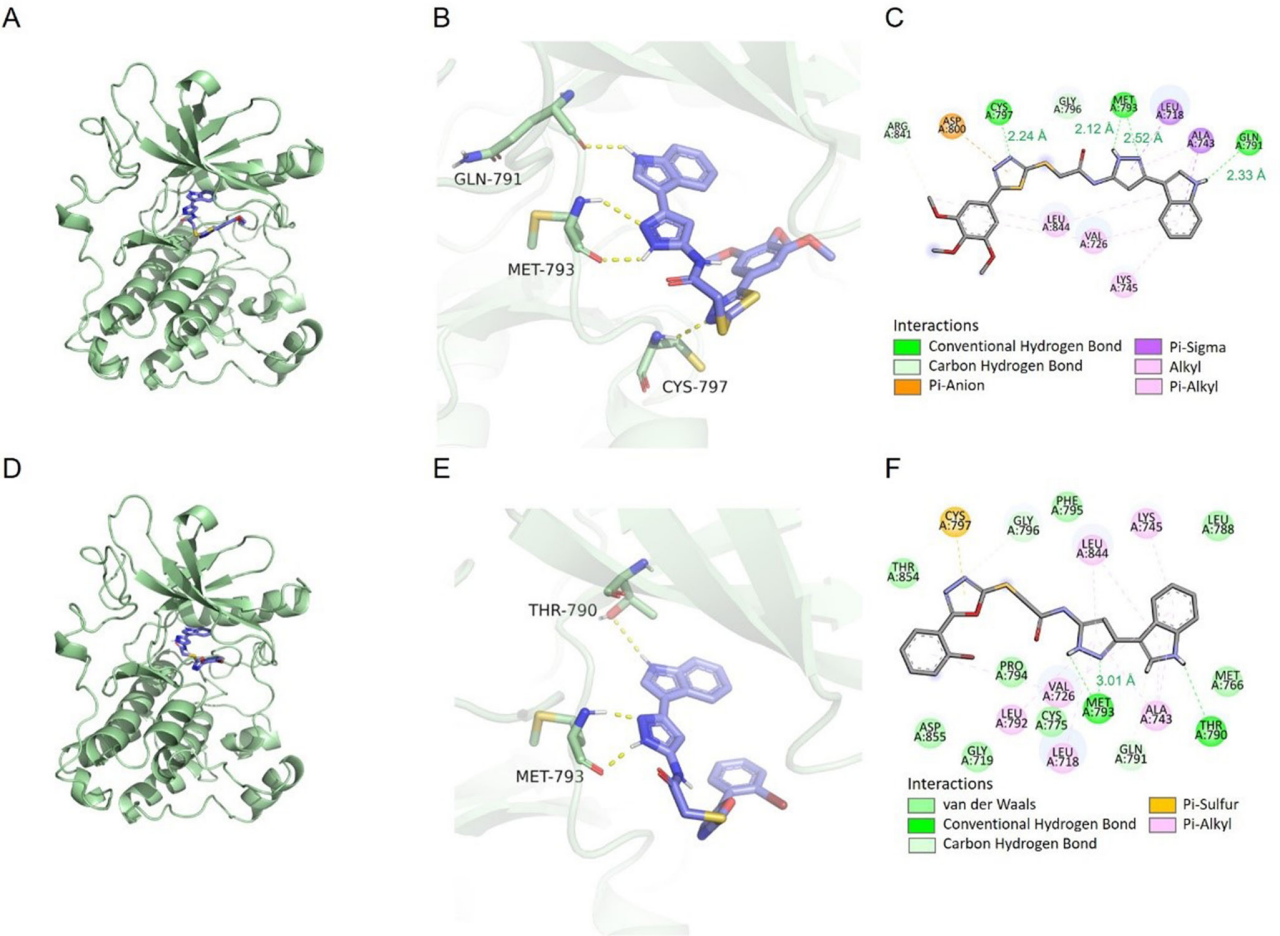
The docking results were analysed visually and binding interactions of compounds **10 b** and **11 h** into EGFR. (A, D) Cartoon like representation showing the co-crystallized Gefitinib into EGFR of compound **10 b** and **11 h**. (B, E) 3 D binding mode of compound **10 b** and **11 h** into the active site of EGFR. (C, F) 2 D binding mode of compound **10 b** and **11 h** in the active site EGFR.

To corroborate the relation between *in vitro* cytotoxicity findings and binding affinities of analogues, docking analysis of the compound **10 b** against EGFR (PDB code: 2ITY) was performed using docking studies, including the binding modes/orientations, affinities, and interactions analysis. The results of the docking study revealed nice fitting of the two compounds into EGFR with absence of any steric clashes or unfavourable interactions. Investigation of the binding interactions of compound **10 b** showed two conventional hydrogen bonds with Met793 (2.12 Å and 2.52 Å), one conventional hydrogen bonds with Cys797 (2.24 Å) and Gln791 (2.33 Å), respectively. Compound **10 b** also showed several hydrophobic interactions of the pi-sigma, pi-alkyl, pi-anion and alkyl types with Leu844, Val726, Lys745, Ala743, Leu718, Asp800 (Figure 9C). The docking score of delta G is minus 8.1, that negative binding affinity indicates the possibility of binding, while smaller value indicates stronger binding. We also examined whether this series of compounds such as **11 h** have similar binding properties. Investigation of the binding interactions of compound **11 h** showed one conventional hydrogen bonds with Gly796 (3.01 Å). Compound **11 h** also showed several hydrophobic interactions of the pi-sigma, pi-alkyl, pi-anion and alkyl types with Met793, Leu718, Cys775, Leu844, Ala743, Val726, Lys745, Asp855 (Figure 9F). The docking score of delta G by **11 h** is minus 8.9, which also has the fine combination ability. Compound **10 b** and **11 h** were superposed with gefitinib, found that although there were some differences in chemical structure, there was still certain superimposition, such as compound **10 b** with an RMSD value of 4.87 Å, and compound **11 h** with an RMSD value of 6.14 Å. Docking results showed that compounds **10 b** and **11 h** could successfully bind to the ATP pocket of EGFR protein, suggesting that it may be an ATP competitive inhibitor (Figure 9).

## Conclusion

Herein, a series of indole derivatives with penta-heterocycles substitutions were reported. The antiproliferative activity values indicated that compound **10 b** stood as the most potent derivative with IC_50_ values of 120 nM and 10 nM, respectively, against A549 and K562 cells. To further investigate the mechanism underlying the antiproliferative effect of **10 b**, the analysis of apoptosis, cell cycle arrest, cell migration, and the expression level and phosphorylation of EGFR and AKT, and p53 related proteins were investigated. Accordingly, it was evident that both DAPI staining and apoptosis morphological analysis observation strongly suggested **10 b** induced apoptosis both in A549 and K562 cells. As illustrated in this study, the flow cytometric results showed that the exposure of A549 and K562 cells to compound **10 b** gave rise to a significant rise in the cell populations at G2/M phase in a dose-dependent manner. Furthermore, **10 b** displayed effects on suppression of EGFR-mediated proteins and promotion of p53 protein and indicating its anti-proliferation characteristics against A549 and K562 cells. In addition, the molecular docking assay of **10 b** was performed to identify the protein-binding properties for the potential active sites of the EGFR. Interestingly enough, both **11 h** and **10 b** applied similar mode to bind with ATP pocket of EGFR protein. However, we still misunderstand the molecular pathway engaged by **11 h** regular consumption and there are still a lot of studies in our future work. In conclusion, this series indole derivatives might serve as promising candidate for further development of potent A549 and K562 inhibitors.

## Experimental section

### Chemistry

The reagents were purchased from commercial sources, used directly. The melting points of the compounds were tested on an X-4D melting point apparatus. ^1^H NMR and ^13^C NMR spectra were recorded on a Bruker DPX 400 MHz (Bruker, Germany), or on a JEOLECX-500 (JEOL, Japan) in DMSO-*d_6_* solution. High-resolution mass spectra were obtained with Thermo Scientific Q Exactive (Thermo Scientific, USA).

### General synthesis

Initially, indole as the starting material was reacted with cyanoacetic acid to obtain intermediate **2** as per the reported method^41^. The cyclic reaction of compound **2** with 80% hydrazine hydrate was carried out to obtain intermediate **3**^42^. The reaction of intermediate **4** with chloroacetyl chloride, in 1,4-dioxane was constructed according to the published procedures with some modifications^43^.

#### General procedure for the synthesis of compounds 10a–i

The different 1,3,4-thiadiazoles (**8a*–*i**) were prepared according to known methods^44–46^. Then, a mixture of the intermediate 1,3,4-thiadiazoles (10 mmol) and potassium hydroxide (20 mmol, 1.1 g) was stirred in anhydrous ethanol (50 ml). A solution of intermediate **4** (10 mmol, 2.7 g) was subsequently added into the mixture, which was stirred at room temperature for 3 h. Then, poured into 200 ml of ice water, when the reaction was completed. The solid was collected through filtration and then purified through crystallisation from absolute alcohol to give pure title compounds (**10a*–*i**).

*N*-*(3*-*(1H*-*indol*-*3*-*yl)*-*1H*-*pyrazol*-*5*-*yl)*-*2*-*((5*-*(3,5*-*dichlorophenyl)-1,3,4-thiadiazol-2-yl)thio)acetamide* (**10a**).

A green solid, yield 64.2%, m. p. 169–171 °C; ^1^H NMR (400 MHz, DMSO-*d_6_*) *δ* 12.57 (*s*, 1H), 11.45 (*s*, 1H), 10.86 (d, *J* = 38.4 Hz, 1H), 8.08–7.69 (*m*, 4H), 7.46 (d, *J* = 7.8 Hz, 1H), 7.28–7.02 (*m*, 3H), 6.85 (d, *J* = 16.2 Hz, 1H), 4.27 (s, 2H). ^13^C NMR (101 MHz, DMSO-*d_6_*) *δ* 167.2, 165.7, 164.7, 164.2, 136.8, 135.6, 132.8, 131.0, 126.4, 124.8, 123.9, 122.3, 121.7, 120.4, 119.5, 112.5, 105.7, 93.3, 43.5. HRMS (ESI)

[M + Na] ^+^ calcd for C_21_H_14_ Cl_2_N_6_OS_2_: 522.9924, found: 522.9940.

*N-(3-(1H-indol-3-yl)-1H-pyrazol-5-yl)-2-((5-(3,4,5-trimethoxyphenyl)-1,3,4-thiadiazol-2-yl)thio)acetamide* (**10 b**).

A yellow solid, yield 27.2%, m. p 261–263 °C; ^1^H NMR (400 MHz, DMSO-*d_6_*) *δ* 12.54 (*s*, 1H), 11.46 (*s*, 1H), 10.88 (*s*, 1H), 7.89–7.68 (*m*, 2H), 7.46 (d, *J* = 8.1 Hz, 1H), 7.32*–*6.99 (*m*, 4H), 6.83 (*s*, 1H), 4.35 (*s*, 2H), 3.91*–*3.84 (*m*, 6H), 3.76*–*3.63 (*m*, 3H). ^13^C NMR (101 MHz, DMSO-*d_6_*) *δ* 168.5, 165.3, 164.8, 153.9, 148.1, 140.4, 138.2, 136.8, 125.1, 124.8, 123.8, 122.3, 120.4, 119.4, 112.5, 105.6, 105.3, 104.0, 93.3, 60.7, 56.6, 38.3. HRMS (ESI) [M-H] ^+^ calcd for C_24_H_22_N_6_O_4_S_2_ 521.1060, found 521.1082.

*N-(3-(1H-indol-3-yl)-1H-pyrazol-5-yl)-2-((5–(2-bromophenyl)-1,3,4-thiadiazol-2-yl)thio)acetamide* (**10c**).

A white solid, yield 21.2%, m. p. 159*–*161 °C; ^1^H NMR (500 MHz, DMSO-*d_6_*) *δ* 12.51 (*s*, 1H), 11.42 (*s*, 1H), 10.89 (*s*, 1H), 7.93 (dd, *J* = 7.8, 1.8 Hz, 1H), 7.81 (dd, *J* = 8.0, 1.2 Hz, 1H), 7.73 (*t*, *J* = 5.1 Hz, 2H), 7.57*–*7.38 (*m*, 3H), 7.18*–*7.04 (*m*, 2H), 6.79 (*s*, 1H), 4.35 (*s*, 2H). ^13^C NMR (126 MHz, DMSO-*d_6_*) *δ* 168.5, 164.9, 164.3, 164.3, 162.1, 148.1, 138.3, 136.8, 129.7, 124.8, 123.9, 123.9, 122.4, 122.3, 120.4, 119.5, 115.4, 112.6, 105.7, 93.4, 43.6. HRMS (ESI) [M*-*H] ^+^ calcd for C_21_H_15_BrN_6_OS_2_ 508.9848, found 508.9865.

*N-(3-(1H-indol-3-yl)-1H-pyrazol-5-yl)-2-((5-(4-chlorobenzyl)-1,3,4-thiadiazol-2-yl)thio)acetamide* (**10d**).

A yellow solid, yield 67.4%, m. p. 151*–*153 °C; ^1^H NMR (400 MHz, DMSO-*d_6_*) δ12.55 (*s*, 1H), 11.48 (*s*, 1H), 10.84 (*s*, 1H), 7.78 (d, *J* = 2.7 Hz, 1H), 7.41 (ddd, *J* = 22.3, 21.4, 8.2 Hz, 6H), 7.20–7.11 (*m*, 2H), 6.82 (d, *J* = 15.7 Hz, 1H), 4.44 (*s*, 2H), 4.30*–*4.24 (*m*, 2H). ^13^C NMR (101 MHz, DMSO-*d_6_*) *δ* 169.9, 165.9, 164.8, 136.8, 136.8, 132.4, 131.2, 129.2, 124.8, 123.9, 123.8, 122.3, 120.3, 119.5, 112.5, 105.7, 93.3, 49.1, 43.5. HRMS (ESI) [M-H] ^+^ calcd for C_22_H_17_ClN_6_OS_2_: 479.0510, found: 479.0519.

*N-(3-(1H-indol-3-yl)-1H-pyrazol-5-yl)-2-((5-(4-methoxyphenyl)-1,3,4-thiadiazol-2-yl)thio)acetamide* (**10e**).

A brown solid, yield 38.6%, m. p. 220*–*222 °C; ^1^HNMR (500 MHz, DMSO-*d_6_*) *δ* 12.51 (d, *J* = 12.5 Hz, 1H), 11.42 (*s*, 1H), 10.81 (d, *J* = 31.5 Hz, 1H), 7.84–7.69 (*m*, 4H), 7.42 (dd, *J* = 7.9, 4.2 Hz, 1H), 7.17*–*7.02 (*m*, 4H), 6.79 (d, *J* = 6.3 Hz, 1H), 4.26 (d, *J* = 31.2 Hz, 3H), 3.78 (*s*, 2H). ^13^CNMR (126 MHz, DMSO-*d_6_*) *δ* 168.5, 164.9, 164.3, 164.3, 162.1, 148.1, 138.3, 136.8, 129.7, 124.8, 123.9, 123.9, 122.4, 122.3, 120.4, 119.5, 115.4, 112.6, 105.7, 93.4, 43.6. HRMS (ESI) [M*-*H] ^+^ calcd for C_22_H_18_N_6_O_2_S_2_: 461.0849, found: 461.0863.

*N-(3-(1H-indol-3-yl)-1H-pyrazol-5-yl)-2-((5-(4-chlorophenyl)-1,3,4-thiadiazol-2-yl)thio)acetamide* (**10f**).

A reddish-brown solid, yield 40.4%, m. p. 175*–*177 °C; ^1^H NMR (400 MHz, DMSO-*d_6_*) *δ* 12.56 (*s*, 1H), 11.47 (*s*, 1H), 10.92 (*s*, 1H), 7.93 (d, *J* = 7.6 Hz, 2H), 7.77 (*s*, 2H), 7.61 (d, *J* = 7.5 Hz, 2H), 7.46 (d, *J* = 6.7 Hz, 1H), 7.15 (dd, *J* = 14.8, 7.0 Hz, 2H), 6.84 (*s*, 1H), 4.37 (*s*, 2H). ^13^C NMR (101 MHz, DMSO-*d_6_*) *δ* 167.4, 166.0, 164.8, 148.1, 138.2, 136.8, 136.4, 130.0, 129.6, 128.6, 124.8, 123.8, 122.3, 120.4, 119.4, 112.5, 105.6, 93.2, 56.5. HRMS (ESI) [M*-*H] ^+^ calcd for C_21_H_15_ClN_6_OS_2_: 465.0354, found: 465.0363.

*N-(3-(1H-indol-3-yl)-1H-pyrazol-5-yl)-2-((5-(2-fluorophenyl)-1,3,4-thiadiazol-2-yl)thio)acetamide* (**10 g**).

A white solid, yield 53.7%, m. p. 163*–*165 °C; ^1^H NMR (400 MHz, DMSO-*d_6_*) *δ* 12.57 (*s*, 1H), 11.47 (*s*, 1H), 10.93 (*s*, 1H), 8.22 (*s*, 1H), 7.77 (d, *J* = 2.4 Hz, 2H), 7.63 (*s*, 1H), 7.54*–*7.37 (*m*, 3H), 7.15 (dd, *J* = 14.4, 7.6 Hz, 2H), 6.84 (*s*, 1H), 4.39 (*s*, 2H). ^13^C NMR (126 MHz, DMSO-*d_6_*) *δ* 167.1, 164.9, 160.7, 160.2, 158.2, 148.1, 138.3, 136.8, 133.9, 128.9, 126.1, 124.9, 123.9, 122.3, 120.4, 119.5, 117.7, 117.2, 112.5, 93.4, 56.6. HRMS (ESI) [M-H] ^+^ calcd for C_21_H_15_FN_6_OS_2_: 449.0649, found: 449.0657.

*N-(3-(1H-indol-3-yl)-1H-pyrazol-5-yl)-2-((5-(pyridine-3-yl)-1,3,4-thiadiazol-2-yl)thio)acetamide* (**10 h**).

A chartreuse solid, yield 77.8%, m. p. 233*–*235 °C; ^1^H NMR (500 MHz, DMSO-*d_6_*) *δ* 12.52 (*s*, 1H), 11.43 (*s*, 1H), 10.89 (*s*, 1H), 9.06 (*s*, 1H), 8.70 (d, *J* = 4.2 Hz, 1H), 8.27 (dd, *J* = 7.9, 1.6 Hz, 1H), 7.73 (*s*, 2H), 7.62*–*7.35 (*m*, 2H), 7.22 *−7.00* (*m*, 2H), 6.80 (*s*, 1H), 4.36 (*s*, 2H). ^13^C NMR (101 MHz, DMSO-*d_6_*) *δ* 166.5, 165.8, 164.7, 152.4, 148.4, 136.8, 135.4, 126.1, 124.9, 123.8, 122.3, 120.4, 119.4, 112.5, 105.6, 93.3, 38.2. HRMS (ESI) [M-H] ^+^ calcd for C_20_H_15_N_7_OS_2_: 432.0696, found: 432.0709.

*N-(3-(1H-indol-3-yl)-1H-pyrazol-5-yl)-2-((5-(pyridine-2-yl)-1,3,4-thiadiazol-2-yl)thio)acetamide* (**10i**).

A chartreuse solid, yield 87.6%, m. p 226*–*228 °C; ^1^H NMR (400 MHz, DMSO-*d_6_*) *δ* 12.56 (*s*, 1H), 11.47 (*s*, 1H), 10.92 (*s*, 1H), 9.10 (*s*, 1H), 8.73 (d, *J* = 3.8 Hz, 1H), 8.39–8.22 (*m*, 1H), 7.85–7.35 (*m*, 4H), 7.26–7.03 (*m*, 2H), 6.83 (*s*, 1H), 4.39 (*s*, 2H). 13 C NMR (101 MHz, DMSO-*d_6_*) *δ* 166.5, 165.7, 164.8, 152.3, 148.4, 148.0, 138.2, 136.7, 135.5, 126.1, 124.9, 124.8, 123.8, 122.3, 120.4, 119.4, 112.5, 105.6, 93.3, 56.5. HRMS (ESI) [M*–*H] ^+^ calcd for C_20_H_15_N_7_OS_2_: 432.0696, found: 432.0707.

#### General procedure for the synthesis of compounds 9a–r

A mixture of the potassium dithiocarbazinate as the respective aromatic esters (**7a*–*r**, 0.1 mol) and 80% hydrazine hydrate (0.3 mol, 18.9 ml) in water (5 ml) was stirred was refluxed for 5*–*8 h. After completion, the reaction was quenched with ice water. Then, the mixture was acidified with dilute HCl to pH 2 to 3. Finally, the crude products were filtered and recrystallized from ethanol to give intermediate **9a*–*r**^47^^,^^48^. Similarly, compounds **11a*–*r** was obtained according to the procedures of compounds **8a*–*i**.

##### N-(3-(1H-indol-3-yl)-1H-pyrazol-5-yl)-2-((4-amino-5–(3,5-dichlorophenyl)-4H-1,2,4-triazol-3-yl)thio)acetamide (11a)

A white solid, yield 45.6%, m. p. 258*–*260 °C; ^1^H NMR (500 MHz, DMSO-*d_6_*) *δ* 12.47 (*s*, 1H), 11.41 (*s*, 1H), 10.75 (*s*, 1H), 8.03 (*s*, 2H), 7.75 (*s*, 2H), 7.42 (d, *J* = 7.8 Hz, 1H), 7.11 (dt, *J* = 14.3, 6.9 Hz, 2H), 6.78 (*s*, 1H), 6.28 (*s*, 2H), 4.14 (*s*, 2H). ^13^C NMR (101 MHz, DMSO-*d_6_*) *δ* 164.4, 163.7, 148.0, 136.8, 133.7, 132.2, 131.6, 131.6, 124.8, 123.8, 122.6, 122.3, 120.3, 119.4, 112.5, 105.6, 93.2, 36.6. HRMS (ESI)

[M-H] ^+^ calcd for C_21_H_16_Cl_2_N_8_OS: 497.0461 found: 497.0473.

##### N-(3-(1H-indol-3-yl)-1H-pyrazol-5-yl)-2-((4-amino-5–(3,4,5-trimethoxyphenyl)-4H-1,2,4-triazol-3-yl)thio)acetamide (11 b)

A white solid, yield 69.3%, m. p. 259*–*261 °C; ^1^H NMR (400 MHz, DMSO-*d_6_*) *δ* 12.53 (*s*, 1H), 11.48 (*s*, 1H), 10.78 (*s*, 1H), 7.76 (d, *J* = 6.6 Hz, 2H), 7.46 (d, *J* = 7.9 Hz, 1H), 7.27 (*s*, 2H), 7.15 (dd, *J* = 13.8, 7.5 Hz, 2H), 6.83 (d, *J* = 1.4 Hz, 1H), 6.27 (*s*, 2H), 4.16 (*s*, 2H), 3.83 (*s*, 7H), 3.72 (*s*, 3H). ^13^C NMR (101 MHz, DMSO-*d_6_*) *δ* 165.7, 154.5, 153.7, 153.4, 148.1, 139.2, 138.1, 136.8, 124.8, 123.8, 122.5, 122.3, 120.3, 119.4, 112.5, 106.0, 105.6, 93.3, 49.0. HRMS (ESI) [M-H] ^+^ calcd for C_24_H_24_N_8_O_4_S: 519.1557 found: 519.1565.

##### N-(3-(1H-indol-3-yl)-1H-pyrazol-5-yl)-2-((4-amino-5–(2-bromophenyl)-4H-1,2,4-triazol-3-yl)thio)acetamide (11c)

A white solid, yield 27.7%, m. p. 194*–*195 °C; ^1^H NMR (400 MHz, DMSO-*d_6_*) *δ* 12.51 (*s*, 1H), 11.45 (*s*, 1H), 10.81 (*s*, 1H), 7.93*–*7.67 (*m*, 3H), 7.65*–*7.39 (*m*, 4H), 7.16 (dd, *J* = 12.9, 7.4 Hz, 2H), 6.84 (*s*, 1H), 5.93 (*s*, 2H), 4.18 (*s*, 2H). ^13^C NMR (101 MHz, DMSO-*d_6_*) *δ* 165.6, 155.1, 152.6, 148.1, 138.1, 136.8, 133.2, 133.1, 132.4, 128.9, 128.0, 124.8, 123.9, 123.8, 122.3, 120.3, 119.4, 112.5, 105.7, 93.3, 67.5, 36.3. HRMS (ESI) [M-H] ^+^ calcd for C_21_H_17_BrN_8_OS: 507.0346 found: 507.0361.

##### N-(3-(1H-indol-3-yl)-1H-pyrazol-5-yl)-2-((4-amino-5–(4-chlorobenzyl)-4H-1,2,4-triazol-3-yl)thio)acetamide (11d)

A white solid, yield 16.9%, m. p. 240*–*241 °C; ^1^H NMR (400 MHz, DMSO-*d_6_*) *δ* 12.49 (*s*, 1H), 11.44 (*s*, 1H), 10.72 (*s*, 1H), 7.76 (d, *J* = 6.4 Hz, 2H), 7.46 (d, *J* = 7.9 Hz, 1H), 7.33 (dt, *J* = 25.0, 6.7 Hz, 4H), 7.15 (dt, *J* = 14.6, 7.0 Hz, 2H), 6.96 (d, *J* = 120.6 Hz, 1H), 5.97 (*s*, 2H), 4.08 (d, *J* = 4.8 Hz, 4H). ^13^C NMR (101 MHz, DMSO-*d_6_*) *δ* 165.7, 155.5, 151.5, 148.1, 138.1, 136.8, 136.0, 131.7, 131.1, 128.8, 124.8, 123.8, 122.3, 120.3, 119.4, 112.5, 105.6, 93.3, 36.3. HRMS (ESI) [M-H] ^+^ calcd for C_22_H_19_ClN_8_OS: 477.1007 found: 477.1018.

##### N-(3-(1H-indol-3-yl)-1H-pyrazol-5-yl)-2-((4-amino-5–(4-methoxyphenyl)-4H-1,2,4-triazol-3-yl)thio)acetamide (11e)

A white solid, yield 86.8%, m. p. 238*–*240 °C; ^1^H NMR (400 MHz, DMSO-*d_6_*) *δ* 12.51 (*s*, 1H), 11.45 (*s*, 1H), 10.78 (*s*, 1H), 8.10*–*7.89 (*m*, 2H), 7.77 (*t*, *J* = 4.5 Hz, 2H), 7.46 (d, *J* = 7.9 Hz, 1H), 7.21*–*7.05 (*m*, 4H), 6.82 (*s*, 1H), 6.20 (*s*, 2H), 4.14 (*s*, 2H), 3.82 (*s*, 3H).^13^C NMR (101 MHz, DMSO-*d_6_*) *δ* 165.8, 160.8, 154.4, 153.1, 148.1, 138.1, 136.8, 129.8, 124.8, 123.8, 122.3, 120.3, 119.7, 119.5, 114.4, 112.5, 105.7, 93.3, 55.8, 36.2. HRMS (ESI) [M-H] ^+^ calcd for C_22_H_20_N_8_O_2_S: 459.1346 found: 459.1365.

##### N-(3-(1H-indol-3-yl)-1H-pyrazol-5-yl)-2-((4-amino-5–(4-chlorophenyl)-4H-1,2,4-triazol-3-yl)thio)acetamide (11f)

A white solid, yield 74.1%, m. p. 214*–*215 °C; ^1^H NMR (500 MHz, DMSO-*d_6_*) *δ* 12.48 (*s*, 1H), 11.42 (*s*, 1H), 10.76 (*s*, 1H), 8.13*–*7.90 (*m*, 2H), 7.72 (*t*, *J* = 4.3 Hz, 2H), 7.59*–*7.53 (*m*, 2H), 7.42 (d, *J* = 8.0 Hz, 1H), 7.20*–*7.02 (*m*, 2H), 6.78 (*s*, 1H), 6.22 (*s*, 2H), 4.13 (*s*, 2H). ^13^C NMR (101 MHz, DMSO-*d_6_*) *δ* 165.7, 154.0, 153.6, 136.8, 135.0, 129.9, 129.1, 126.2, 124.8, 123.8, 122.3, 120.3, 119.5, 112.5, 93.3, 36.2. HRMS (ESI) [M-H] ^+^ calcd for C_21_H_17_ClN_8_OS: 463.0851 found: 463.0877.

##### N-(3-(1H-indol-3-yl)-1H-pyrazol-5-yl)-2-((4-amino-5–(2-fluorophenyl)-4H-1,2,4-triazol-3-yl)thio)acetamide (11 g)

A white solid, yield 43.3%, m. p. 226*–*227 °C; ^1^H NMR (500 MHz, DMSO-*d_6_*) *δ* 12.49 (s), 11.43 (s), 10.79 (s), 7.73 (d, *J* = 7.2 Hz), 7.64 (dd, *J* = 7.3, 6.0 Hz), 7.56 (d, *J* = 7.9 Hz), 7.36 (ddd, *J* = 20.1, 19.5, 7.7 Hz), 7.11 (dd, *J* = 16.9, 7.6 Hz), 6.80 (d, *J* = 1.1 Hz), 6.02 (s), 4.15 (s). ^13^C NMR (126 MHz, DMSO-*d_6_*) *δ* 165.7, 161.2, 159.2, 153.5, 151.6, 148.2, 138.1, 136.8, 133.0, 132.9, 132.2, 125.1, 124.8, 123.9, 122.4, 120.4, 119.5, 116.7, 116.6, 115.5, 115.4, 112. 6, 105.7, 93.4, 49.1. HRMS (ESI) [M-H] ^+^ calcd for C_21_H_17_FN_8_OS: 447.1146 found: 447.1152.

##### N-(3-(1H-indol-3-yl)-1H-pyrazol-5-yl)-2-((4-amino-5-(pyridine-3-yl)-4H-1,2,4-triazol-3-yl)thio)acetamide (11 h)

A yellow solid, yield 62.1%, m. p. 195*–*197 °C; ^1^H NMR (500 MHz, DMSO-*d_6_*) *δ* 12.51 (d, *J* = 24.6 Hz), 11.42 (s), 10.76 (d, *J* = 9.0 Hz), 9.11 (d, *J* = 1.7 Hz), 8.65 (dd, *J* = 4.8, 1.6 Hz), 8.38*–*8.28 (m), 7.77*–*7.67 (m), 7.53 (ddd, *J* = 8.0, 4.9, 0.8 Hz), 7.42 (d, *J* = 8.0 Hz), 7.14*–*7.06 (m), 6.86 − 6.71 (m), 6.27 (s), 4.19 (d, *J* = 44.2 Hz). ^13^C NMR (126 MHz, DMSO-*d_6_*) *δ* 165.7, 164.3, 154.4, 152.7, 151.0, 148.7, 148.1, 138.1, 136.8, 135.6, 124.8, 124.2, 123.9, 123.9, 123.6, 122.3, 120.4, 119.5, 112.5, 105.7, 93.4, 43.6. HRMS (ESI) [M-H] ^+^ calcd for C_20_H_17_N_9_OS: 430.1193 found: 430.1213.

##### N-(3-(1H-indol-3-yl)-1H-pyrazol-5-yl)-2-((4-amino-5-(pyridine-2-yl)-4H-1,2,4-triazol-3-yl)thio)acetamide (11i)

A white solid, yield 43.8%, m. p. 208*–*210 °C; ^1^H NMR (500 MHz, DMSO-*d_6_*) *δ* 12.48 (*s*, 1H), 11.42 (*s*, 1H), 10.77 (*s*, 1H), 8.67 (d, *J* = 4.3 Hz, 1H), 8.15*–*7.84 (*m*, 2H), 7.72 (*s*, 2H), 7.45 (dd, *J* = 35.6, 6.7 Hz, 2H), 7.11 (dd, *J* = 18.0, 7.5 Hz, 2H), 6.80 (*s*, 1H), 6.59 (*s*, 2H), 4.17 (*s*, 2H). ^13^C NMR (126 MHz, DMSO-*d_6_*) *δ* 165.6, 153.5, 151.5, 149.5, 148.2, 147.4, 146.8, 138.3, 138.1, 136.8, 125.0, 124.8, 123.8, 122.7, 122.3, 120.4, 119.5, 112.5, 105.7, 93.3, 56.6. HRMS (ESI) [M-H] ^+^ calcd for C_20_H_17_N_9_OS: 430.1193 found: 430.1120.

##### N-(3-(1H-indol-3-yl)-1H-pyrazol-5-yl)-2-((4-amino-5–(2-chlorophenyl)-4H-1,2,4-triazol-3-yl)thio)acetamide (11j)

A white solid, yield 59.9%, m. p. 136*–*138 °C; ^1^H NMR (400 MHz, DMSO-*d_6_*) *δ* 12.55 (*s*, 1H), 11.48 (*s*, 1H), 10.84 (*s*, 1H), 7.78 (d, *J* = 6.7 Hz, 2H), 7.68*–*7.41 (*m*, 5H), 7.16 (dt, *J* = 14.6, 6.9 Hz, 2H), 6.85 (*s*, 1H), 5.98 (*s*, 2H), 4.20 (*s*, 2H). ^13^C NMR (101 MHz, DMSO-*d_6_*) *δ* 165.6, 154.0, 152.9, 148.1, 138.1, 136.8, 133.9, 132.9, 132.28, 130.1, 127.6, 126.7, 124.8, 123.8, 122.3, 120.4, 119.4, 112.5, 105.7, 93.3, 36.2. HRMS (ESI) [M + Na] ^+^ calcd for C_21_H_17_ClN_8_OS: 487.0826 found: 487.0818.

##### N-(3-(1H-indol-3-yl)-1H-pyrazol-5-yl)-2-((4-amino-5-phenyl-4H-1,2,4-triazol-3-yl)thio)acetamide (11k)

A white solid, yield 32.6%, m. p. >300 °C; ^1^H NMR (400 MHz, DMSO-*d_6_*) *δ* 12.62 (*s*, 1H), 11.64 (*s*, 1H), 10.81 (*s*, 1H), 8.14*–*7.93 (*m*, 2H), 7.79 (dd, *J* = 23.4, 4.4 Hz, 2H), 7.58*–*7.44 (*m*, 4H), 7.23*–*7.04 (*m*, 2H), 6.80 (*s*, 1H), 6.28 (s, 2H), 4.16 (*s*, 2H).^13^C NMR (101 MHz, DMSO-*d_6_*) *δ* 165.7, 154.6, 153.7, 136.8, 130.1, 129.0, 128.3, 127.3, 124.8, 123.9, 122.2, 120.3, 119.4, 112.5, 93.2, 36.2. HRMS (ESI) [M-H] ^+^ calcd for C_21_H_18_N_8_OS: 429.1241 found: 429.1262.

##### N-(3-(1H-indol-3-yl)-1H-pyrazol-5-yl)-2-((4-amino-5–(4-aminophenyl)-4H-1,2,4-triazol-3-yl)thio)acetamide (11 l)

A white solid, yield 21.1%, m. p. 213*–*215 °C; ^1^H NMR (400 MHz, DMSO-*d_6_*) *δ* 12.50 (*s*, 1H), 11.44 (*s*, 1H), 10.76 (*s*, 1H), 7.76 (d, *J* = 2.6 Hz, 1H), 7.68 (d, *J* = 8.6 Hz, 2H), 7.46 (d, *J* = 7.9 Hz, 1H), 7.21–7.09 (*m*, 2H), 6.63 (d, *J* = 8.7 Hz, 2H), 6.09 (*s*, 2H), 5.53 (*s*, 2H), 4.10 (*s*, 2H). ^13^C NMR (126 MHz, DMSO-*d_6_*) *δ* 165.9, 155.2, 152.3, 150.7, 148.1, 136.8, 129.3, 124.9, 123.9, 122.3, 120.4, 119.5, 114.3, 113.7, 112.5, 105.7, 93.4, 43.6. HRMS (ESI) [M-H] ^+^ calcd for C_21_H_19_N_9_OS: 444.1350 found: 444.1371.

##### N-(3-(1H-indol-3-yl)-1H-pyrazol-5-yl)-2-((4-amino-5–(4-bromophenyl)-4H-1,2,4-triazol-3-yl)thio)acetamide (11 m)

A yellow solid, yield 53.4%, m. p. 270*–*272 °C; ^1^H NMR (500 MHz, DMSO-*d_6_*) *δ* 12.47 (*s*, 1H), 11.41 (*s*, 1H), 10.74 (*s*, 1H), 7.94*–*7.91 (*m*, 2H), 7.73*–*7.68 (*m*, 4H), 7.42 (d, *J* = 8.0 Hz, 1H), 7.16 *−7.06* (*m*, 2H), 6.78 (d, *J* = 1.9 Hz, 1H), 6.21 (*s*, 2H), 4.12 (*s*, 2H). ^13^C NMR (126 MHz, DMSO-*d_6_*) *δ* 165.7, 154.1, 153.7, 136.8, 132.1, 130.1, 126.5, 124.8, 123.9, 123.8, 122.3, 120.4, 119.5, 112.5, 105.7, 93.4, 36.2. HRMS (ESI)

[M-H] ^+^ calcd for C_21_H_17_BrN_8_OS: 507.0346 found 507.0367.

##### N-(3-(1H-indol-3-yl)-1H-pyrazol-5-yl)-2-((4-amino-5-(1H-indol-3-yl)-4H-1,2,4-triazol-3-yl)thio)acetamide (11n)

A white solid, yield 42.5%, m. p. 230*–*232 °C; ^1^H NMR (400 MHz, DMSO-*d_6_*) *δ* 13.72 (*s*, 1H), 12.54 (*s*, 1H), 11.46 (*s*, 1H), 10.84 (*s*, 1H), 8.26 (d, *J* = 8.1 Hz, 1H), 7.88*–*7.59 (*m*, 4H), 7.48 (*t*, *J* = 8.2 Hz, 2H), 7.29 (*t*, *J* = 7.5 Hz, 1H), 7.21*–*7.09 (*m*, 2H), 6.85 (*s*, 1H), 6.40 (*s*, 2H), 4.23 (*s*, 2H). ^13^C NMR (101 MHz, DMSO-*d_6_*) *δ* 165.7, 152.8, 149.7, 140.8, 136.8, 133.4, 127.6, 124.8, 123.8, 122.3, 122.3, 122.1, 121.4, 120.3, 119.5, 112.5, 111.0, 93.3, 35.4. HRMS (ESI) [M + H] ^+^ calcd for C_23_H_19_N_9_OS: 470.1506 found: 470.1477.

##### N-(3-(1H-indol-3-yl)-1H-pyrazol-5-yl)-2-((4-amino-5-(pyridin-4-yl)-4H-1,2,4-triazol-3-yl)thio)acetamide (11o)

A reddish*-*brown solid, yield 41.1%, m. p. 170*–*172 °C; ^1^H NMR (400 MHz, DMSO-*d_6_*) *δ* 12.54 (*s*, 1H), 11.47 (*s*, 1H), 10.80 (d, *J* = 18.0 Hz, 1H), 8.91*–*8.41 (*m*, 2H), 7.89 (dd, *J* = 101.8, 4.1 Hz, 4H), 7.46 (d, *J* = 7.9 Hz, 1H), 7.31*–* 6.98 (*m*, 2H), 6.82 (*s*, 1H), 6.27 (d, *J* = 83.6 Hz, 2H), 4.20 (*s*, 2H). ^13^C NMR (101 MHz, DMSO-*d_6_*) *δ* 165.7, 165.6, 155.2, 152.5, 150.6, 148.1, 146.8, 138.1, 136.7, 134.4, 124.8, 123.8, 122.3, 121.8, 120.4, 119.4, 112.5, 105.6, 93.3, 36.0. HRMS (ESI) [M-H] ^+^ calcd for C_20_H_17_N_9_OS: 430.1193 found: 430.1200.

##### N-(3-(1H-indol-3-yl)-1H-pyrazol-5-yl)-2-((4-amino-5–(4-fluorophenyl)-4H-1,2,4-triazol-3-yl)thio)acetamide (11p)

A brown solid, yield 33.2%, m. p. 209*–*212 °C; ^1^H NMR (500 MHz, DMSO-*d_6_*) *δ* 12.46 (*s*, 1H), 11.40 (*s*, 1H), 10.75 (*s*, 1H), 8.01 (*s*, 2H), 7.71 (*s*, 2H), 7.48*–*7.27 (*m*, 3H), 7.22*–*6.97 (*m*, 3H), 6.77 (*s*, 1H), 6.20 (*s*, 2H), 4.11 (*s*, 2H). ^13^C NMR (126 MHz, DMSO-*d_6_*) *δ* 165.75, 164.4, 162.4, 153.8, 148.2, 138.1, 136.8, 130.7, 130.6, 124.8, 123.9, 122.3, 120.4, 119.5, 116.2, 116.1, 112.5, 105.71, 93.3, 36.2. HRMS (ESI) [M-H] ^+^ calcd for C_21_H_17_FN_8_OS: 447.1146 found: 447.1156.

##### N-(3-(1H-indol-3-yl)-1H-pyrazol-5-yl)-2-((4-amino-5–(2-hydroxyphenyl)-4H-1,2,4-triazol-3-yl)thio)acetamide (11q)

A brown solid, yield 43.9%, m. p. 263*–*266 °C; ^1^H NMR (400 MHz, DMSO-*d_6_*) *δ* 12.53 (*s*, 1H), 11.47 (*s*, 1H), 11.08 (*s*, 1H), 10.83 (*s*, 1H), 7.88*–*7.73 (*m*, 3H), 7.47 (d, *J* = 7.9 Hz, 1H), 7.42*–*7.34 (*m*, 1H), 7.21*–*7.10 (*m*, 2H), 7.00 (ddd, *J* = 15.1, 8.2, 4.3 Hz, 2H), 6.85 (*s*, 1H), 6.11 (*s*, 2H), 4.19 (*s*, 2H). ^13^C NMR (101 MHz, DMSO-*d_6_*) *δ* 165.6, 156.1, 154.3, 153.3, 148.1, 138.1, 136.8, 132.0, 130.0, 124.8, 123.8, 122.29, 120.3, 119.7, 119.5, 116.9, 113.3, 112.5, 105.7, 93.3, 35.6. HRMS (ESI) [M-H] ^+^ calcd for C_21_H_18_N_8_O_2_S: 445.1190 found: 445.1194.

##### N-(3-(1H-indol-3–yl)-1H-pyrazol-5-yl)-2-((4-amino-5-(1H-pyrrol-2-yl)-4H-1,2,4-triazol-3-yl)thio)acetamide (11r)

A grey solid, yield 45.4%, m. p. 241*–*243 °C; ^1^H NMR (500 MHz, DMSO-*d_6_*) *δ* 12.48 (s), 11.60 (s), 11.42 (s), 10.74 (s), 7.73 (d, *J* = 7.6 Hz), 7.42 (d, *J* = 7.9 Hz), 7.11 (dd, *J* = 16.6, 7.5 Hz), 6.95*–*6.72 (m), 6.21*–*6.08 (m), 4.08 (s). ^13^C NMR (126 MHz, DMSO-*d_6_*) *δ* 165.9, 152.0, 150.1, 148.1, 138.2, 136.8, 124.8, 123.9, 122.4, 121.3, 120.4, 119.5, 118.3, 112.6, 110.1, 109.3, 105.7, 93.4, 36.3. HRMS (ESI) [M-H] ^+^ calcd for C_19_H_17_N_9_OS: 418.1193 found: 418.1203.

### Evaluation of biological activity

#### MTT assay

A549, PC-3, HepG2 and K562 cells were purchased from the Kunming cell bank of the Chinese academy of sciences. All cell lines were cultured in DMEM or RPMI 1640 complete medium. Logarithmic growth cells were cultured in 96-well plates at a concentration of 10^4^ cells per well and pre-cultured for 24 h before adding test compounds. The MTT test with 5-Fluorouracil and Gefitinib as reference compounds were used to evaluate antiproliferative activity of target compounds in four human cancer line cells. For the estimation of the inhibition concentration (IC_50_), the Graph Pad Prism (GraphPad Software, San Diego, CA) was used.

#### Apoptosis analysis

DAPI staining assay, A549 or K562 cells were separately seeded at 4.0 × 10^5^ cells per well in six*–*well plates and incubated for 24 h until adherent. Next, the cells were treated with **10 b** at 0, 1, 2 and 4 µM for 48 h. Then the cells were fixed with 4% paraformaldehyde for 15 min, and 1 ml/well of DAPI staining dye was added for 20 min in the dark. After adding the anti-fluorescence quencher, the cells can be imaged by an inverted fluorescence microscope.

Annexin-V/FITC assay, after the adherent cells grew stably in the 6-well plate, each group was reacted with different concentrations of compound solutions for 48 h. Then, cells were collected by trypsinization without EDTA, rinsed twice with PBS, followed by the Annexin V-FITC/PI apoptosis detection kit (Solarbio, Bei Jing, China) according to the kit instructions, flow cytometry by BD FACSCalibur instrument (American BD Company Shanghai Co., Ltd.).

#### Cell cycle analysis

A549 or K562 cells were separately seeded at 4.0 × 10^5^cells/well in six-well plates and incubated for 24 h until adherent. Compound **10 b** were added at 0, 1, 2 and 4 µM for 24 h. After treatment, the cells were collected, and washed twice with pre-cooled PBS and then fixed with ice-cold ethanol (70%), subsequently kept 4 °C for overnight. After washing twice with PBS, cells were incubated at 37 °C with 100 µ L RNase A for 30 min. After this, cells were incubated with 400 µL PI for 30 min at 4 °C. Finally, the cell cycle distribution of compound **10 b** was analysed via the BD FACSCalibur flow cytometry (American BD Corporation Shanghai Co. Ltd.)

#### Western blot assay

Cells were treated with different concentrations of compound for 48 h. After incubation, the cells were washed and collected. Total cell extracts were prepared with RIPA buffer, containing protease inhibitor and phosphatase inhibitor. The lysate was incubated at 0 °C for 60 min, and centrifuged at 12,000 rpm for 15 min at 4 °C to remove cellular debris. Next, the protein concentration was detected using BCA protein concentration assay kit (Beyotime, Shanghai). 20 µg of total protein was subjected to gel electrophoresis and transferred to PVDF membranes (Millipore, Burlington, MA, USA). After blocking in 5% BSA blocking buffer for 60 min, then incubated with primary antibody overnight at 4 °C, washed three times with TBST for 5 min each, followed by incubation with secondary antibody and washed. The blot was developed and exposed by chemiluminescence developer solution, which was placed on a chemiluminescence developer (Pierce, Rockford, Illinois, USA).

#### Wound healing assay

After 24 h of growth, cells were placed in a 6-well plate, scratched using a 10 µL pipet tip on the cell surface. Then, gently wash twice with medium to remove the detached cells. The cells were treated with compound **10 b** with 0.5 and 1.0 µM followed by 48 h incubation and photographed using an inverted microscope.

### Molecular docking

The computational docking study into the active site of EGFR (PDB code: 2ITY) was performed by PyMol 2.3.2 and AutoDock Vina 1.1.2^49^^,^^50^. The crystal structure was retrieved from the RCSB protein data bank (www.rcsb.org). Prior to docking, the receptor protein was treated with PyMol 2.5^51^, including removal of water molecules, salt ions, and small molecule Gefitinib, with the centre of mass of crystal ligand as the box centre, the box size was 22.5 * 22.5 * 22.5 cubic angstroms, subsequently the polar hydrogen atoms were added to the crystal structure. In addition, all processed small molecules and receptor proteins were converted to the required PDB QT format using ADFR suite 1.0 software^52^. Then, the global search detail was set to 32, other parameters ware default. The docking conformation with the highest output score was considered as the binding conformation, and the discovery Studio Viewer docking result was used for visual analysis^53^.

## Supplementary Material

Supplemental Material

## Supplementary data

^1^H NMR, ^13^C NMR and HR-MS spectra for the target compounds (Figures S1−S81), ^1^H NMR and ^13^C NMR spectra for t key intermediate 4(Figures S82, S83), the results of superimposition on molecular docking (Figures S84, S85) associated with this article can be found in the online version.
