# Supplementary material for: Synthesis and biological assessment of indole derivatives containing penta-heterocycles scaffold as novel anticancer agents towards A549 and K562 cells
Source: J Enzyme Inhib Med Chem. 2023 Jan 11;38(1):2163393. doi: 10.1080/14756366.2022.2163393 (PMC9848270; doi:10.1080/14756366.2022.2163393)
Supplement: Supplemental Material [file IENZ_A_2163393_SM5046.pdf]

Synthesis and biological assessment of indole derivatives containing penta-heterocycles scaffold as novel anticancer agents toward A549 and K562 cells

Guanglong Zhang [a, §], Zhenhua Tang [b, §], Sili Fan [b], Chengpeng Li [b], Yan Li [a], Weiqin Liu[b], Xuesha Long [b], Wenjing Zhang [b], Yi Zhang [b], Zhurui Li [b], Zhenchao Wang [a, b, c*], Danping Chen [b*] & Guiping Ouyang [a, b, c*]

[a] State Key Laboratory Breeding Base of Green Pesticide and Agricultural Bioengineering, Key Laboratory of Green Pesticide and Agricultural Bioengineering, Ministry of Education, Center for Research and Development of Fine Chemicals of Guizhou University, Guiyang 550025, China;

[b] College of Pharmacy, Guizhou University, Guiyang 550025, China;

[c] Guizhou Engineering Laboratory for Synthetic Drugs, Guizhou University, Guiyang 550025, China.

[§] Both authors contributed equally to this work.

* Corresponding authors.

Email: wzc.4884@163.com (Zhenchao Wang); Ouyangoygp710@163.com (Guiping Ouyang).

1. ^{1}H NMR, ^{13}C NMR and HRMS spectra for target compounds....................2

2. ^{1}H NMR and ^{13}C NMR spectra for key intermediate **4**....................42

3. Superimposition of the docked ligand of gefitinib with **10b** and **11h**, respectively....................44

4. Table S1. *In vitro* inhibitory activities of target compounds for human cancer cell lines A549, PC-3，HepG2, K562 and HEK293....................45

Information Classification: General

1. ^{1}H NMR, ^{13}C NMR and HRMS spectra for target compounds.

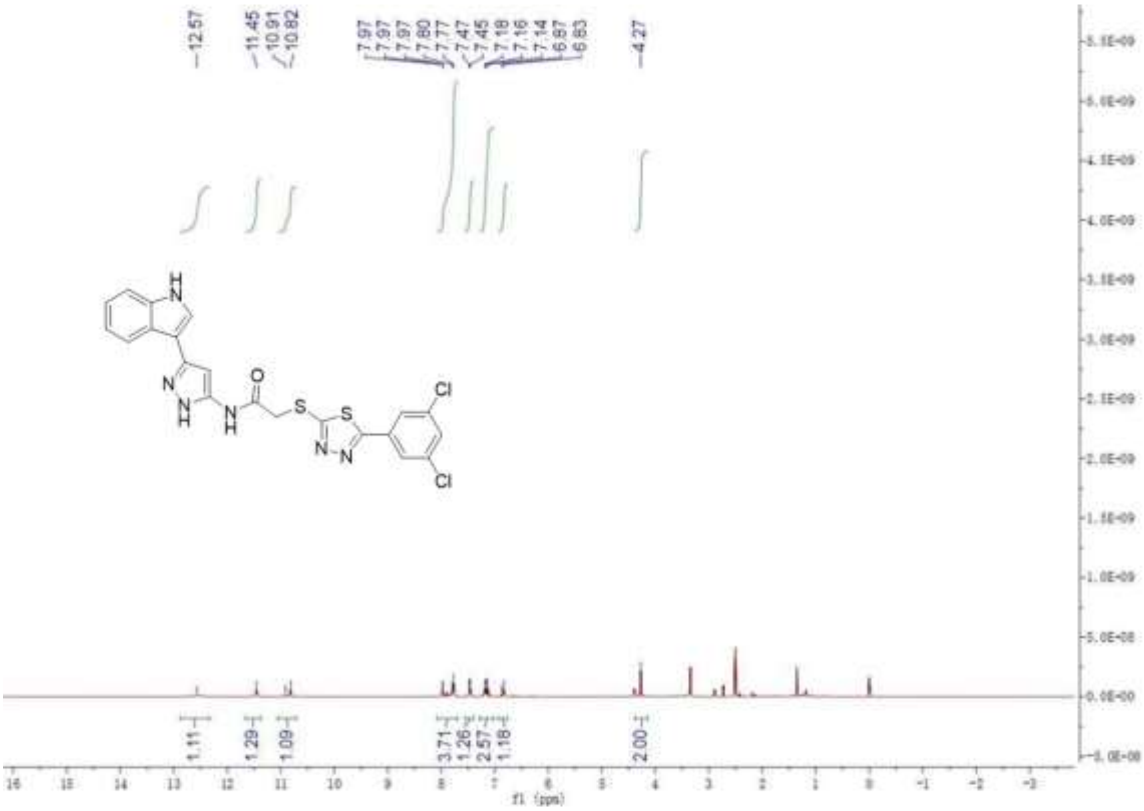

Figure S1. ^{1}H NMR Spectrum (DMSO-d_6, 500 MHz) of 10a.

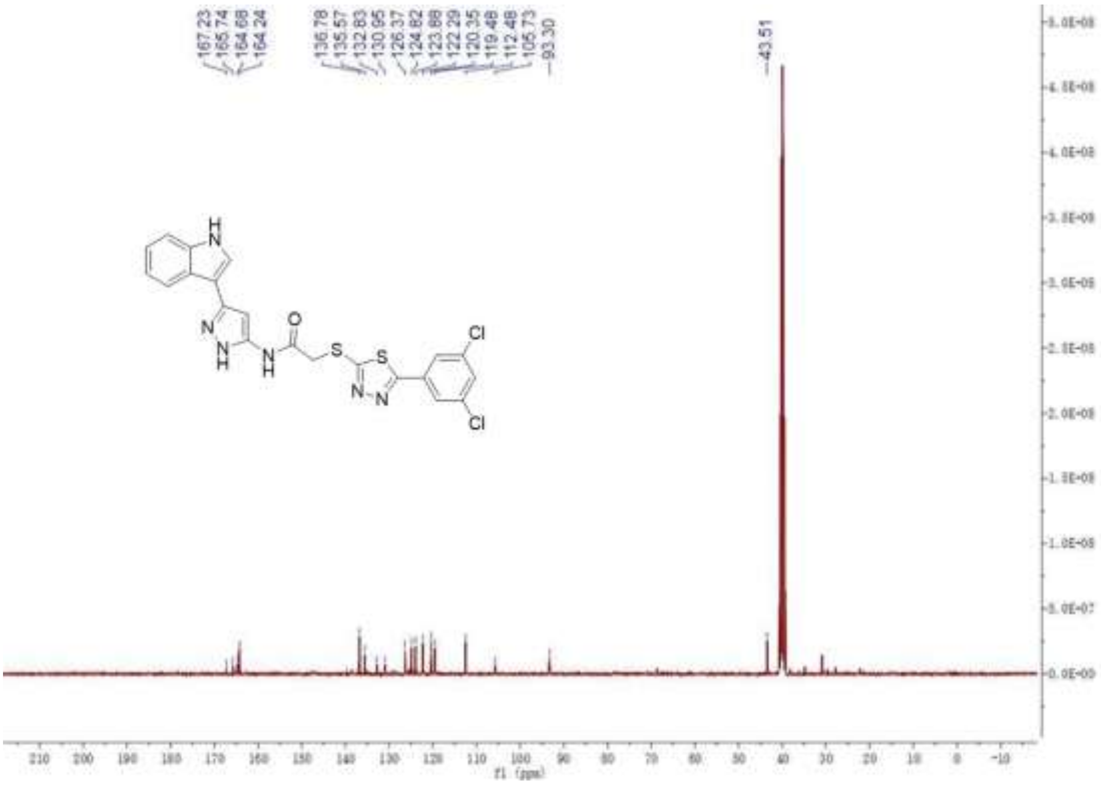

Figure S2. ^{13}C NMR Spectrum (DMSO-d_6, 126 MHz) of 10a.

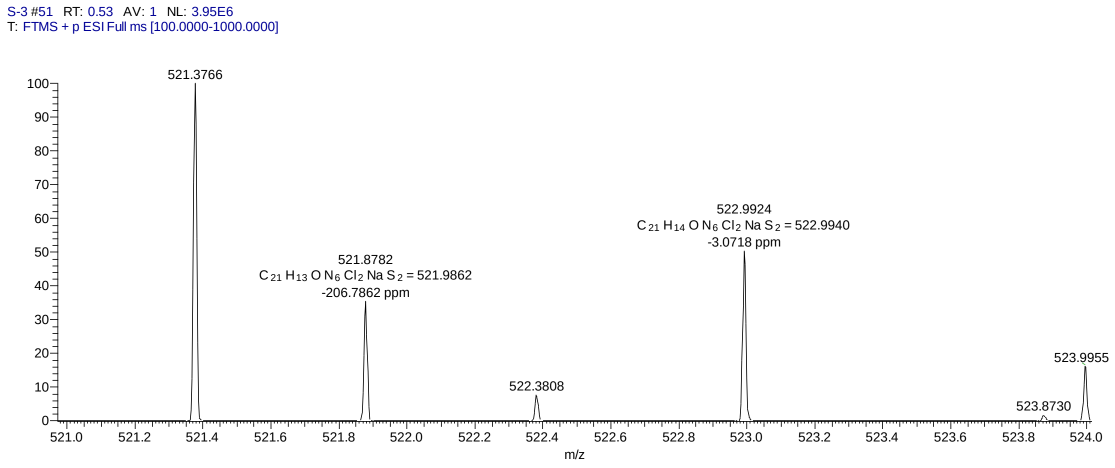

Figure S3. HR-MS Spectrum of 10a.

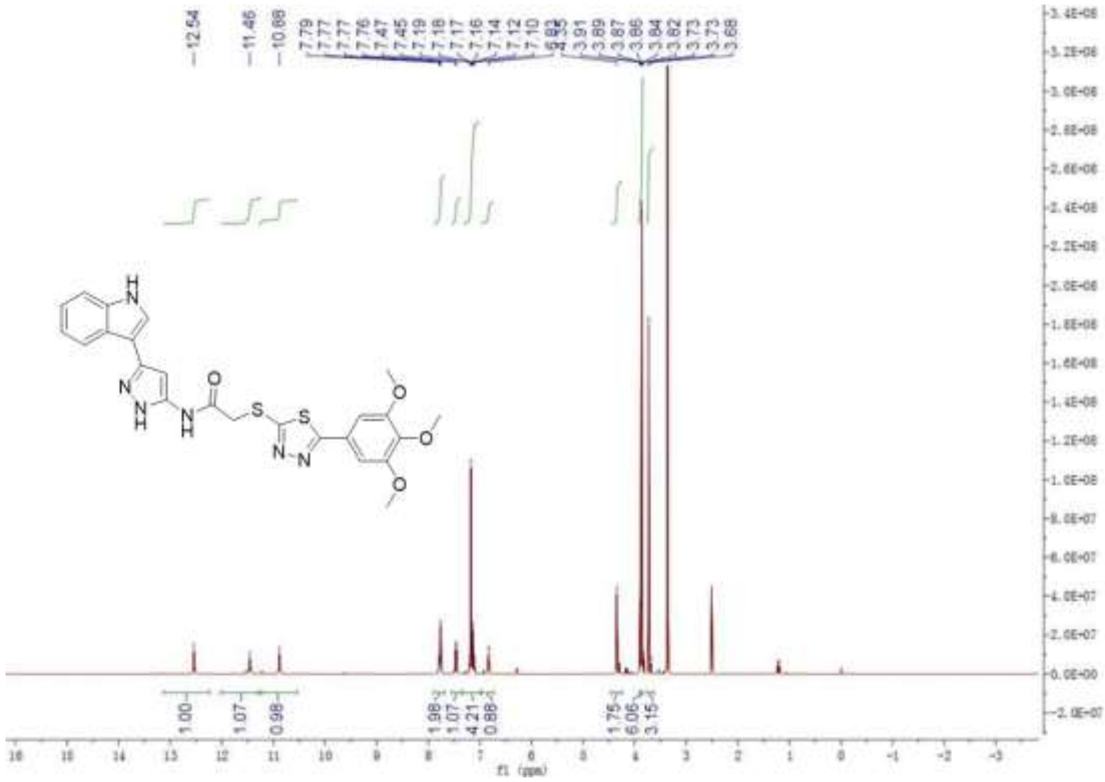

Figure S4. ^{1}H NMR Spectrum (DMSO-d_6, 500 MHz) of 10b.

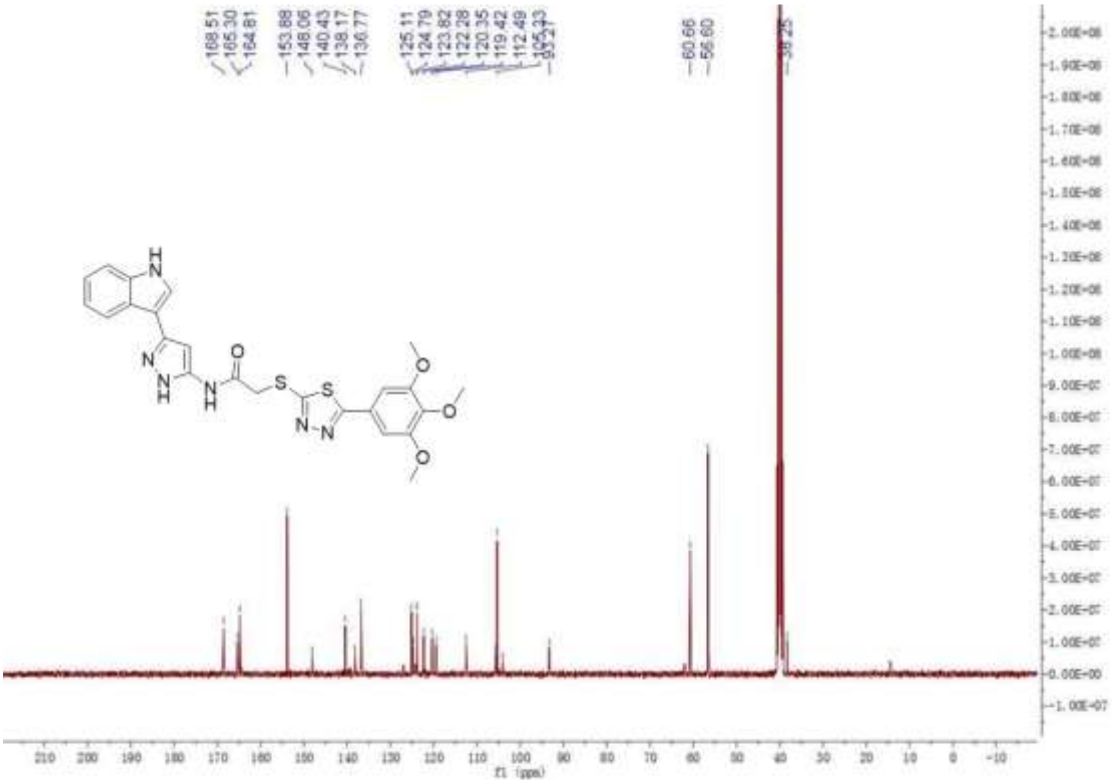

Figure S5. ^{13}C **NMR Spectrum (DMSO-d_6, 126 MHz) of 10b.**

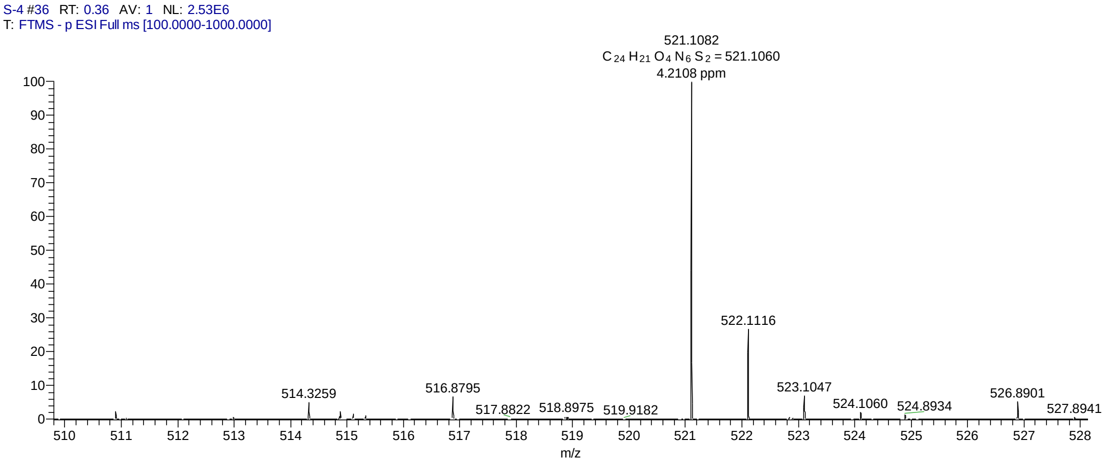

Figure S6. HR-MS Spectrum of 10b.

Information Classification: General

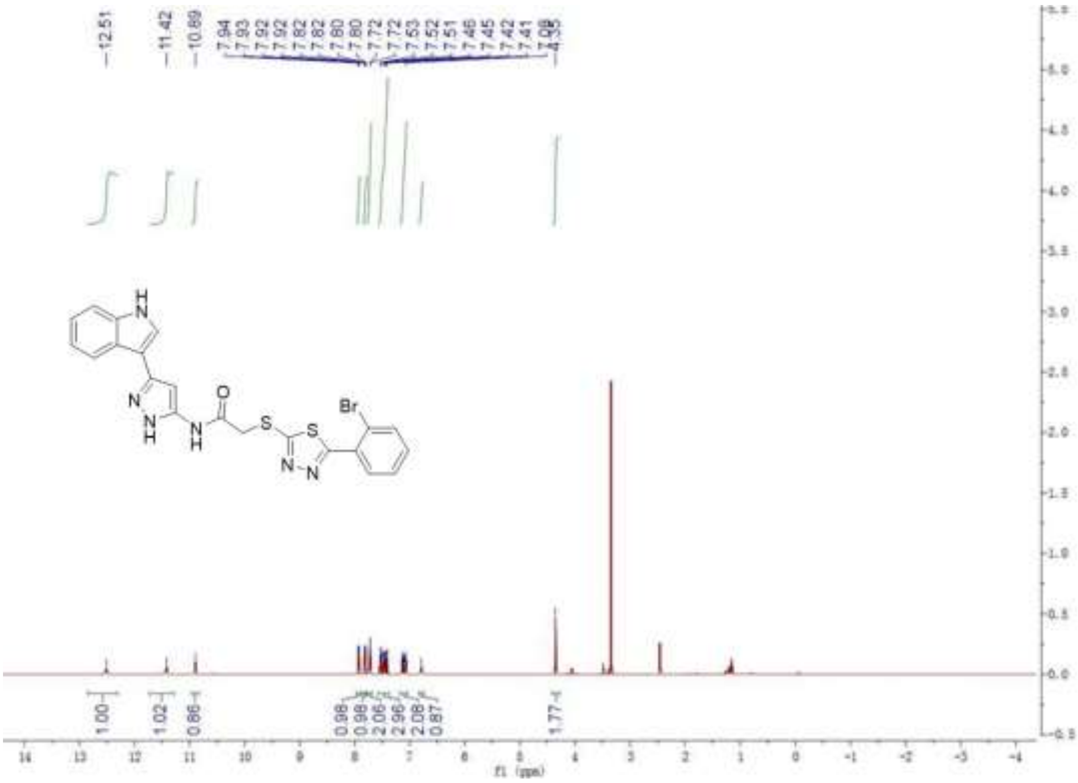

Figure S7. **^{1}H NMR Spectrum (DMSO-d_6, 500 MHz) of 10c.**

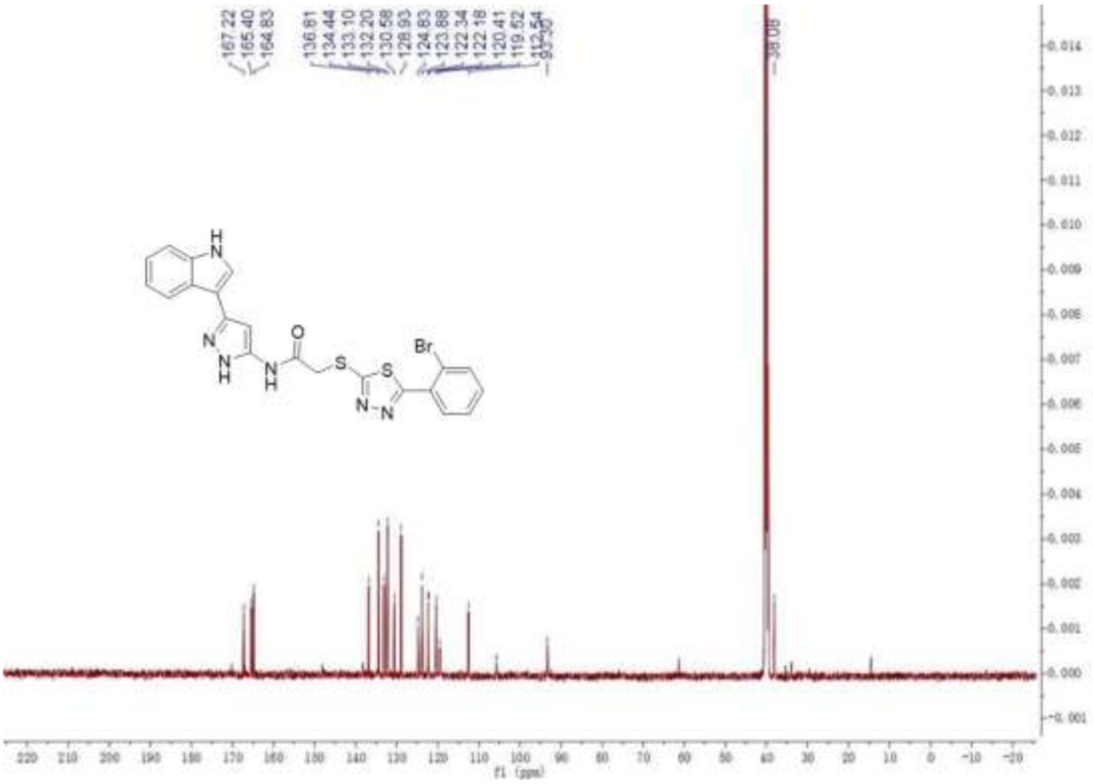

Figure S8. **^{13}C NMR Spectrum (DMSO-d_6, 126 MHz) of 10c.**

Information Classification: General

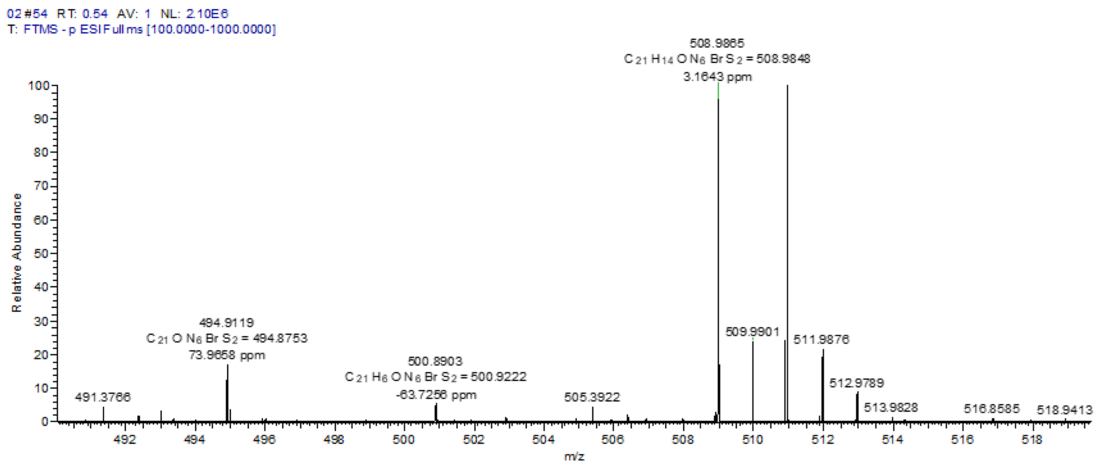

Figure S9. HR-MS Spectrum of 10c.

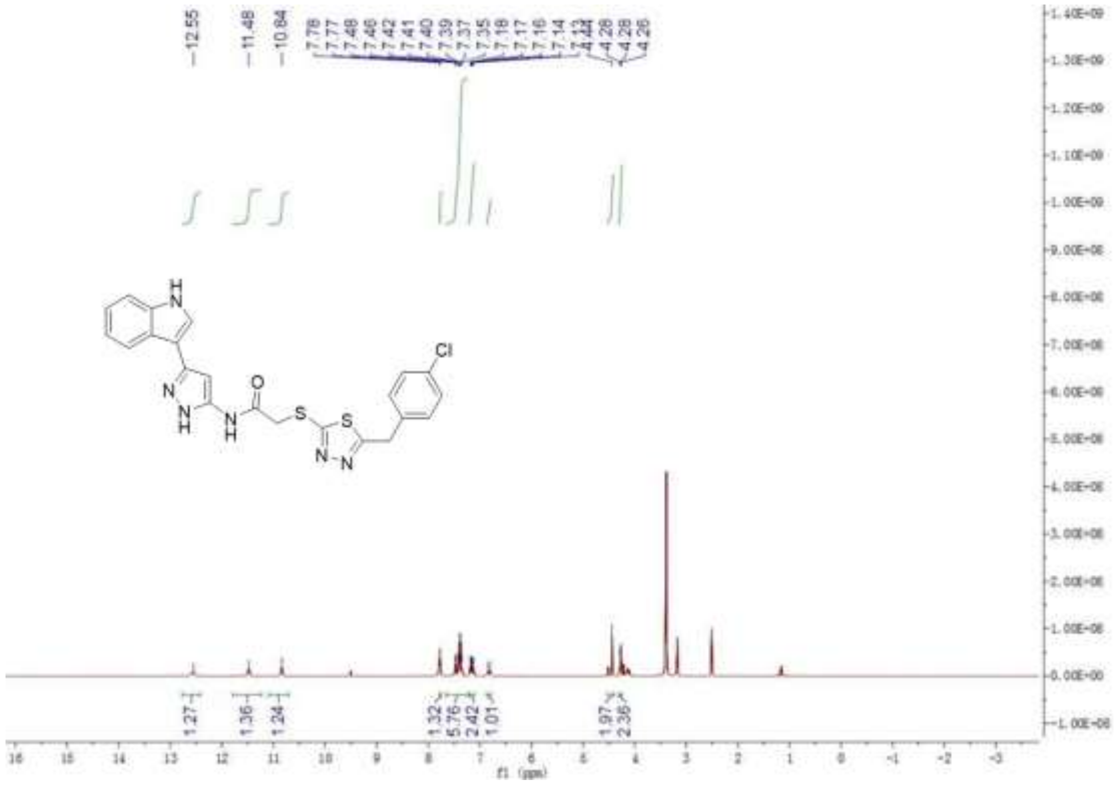

Figure S10. ^{1}H NMR Spectrum (DMSO-d_6, 500 MHz) of 10d.

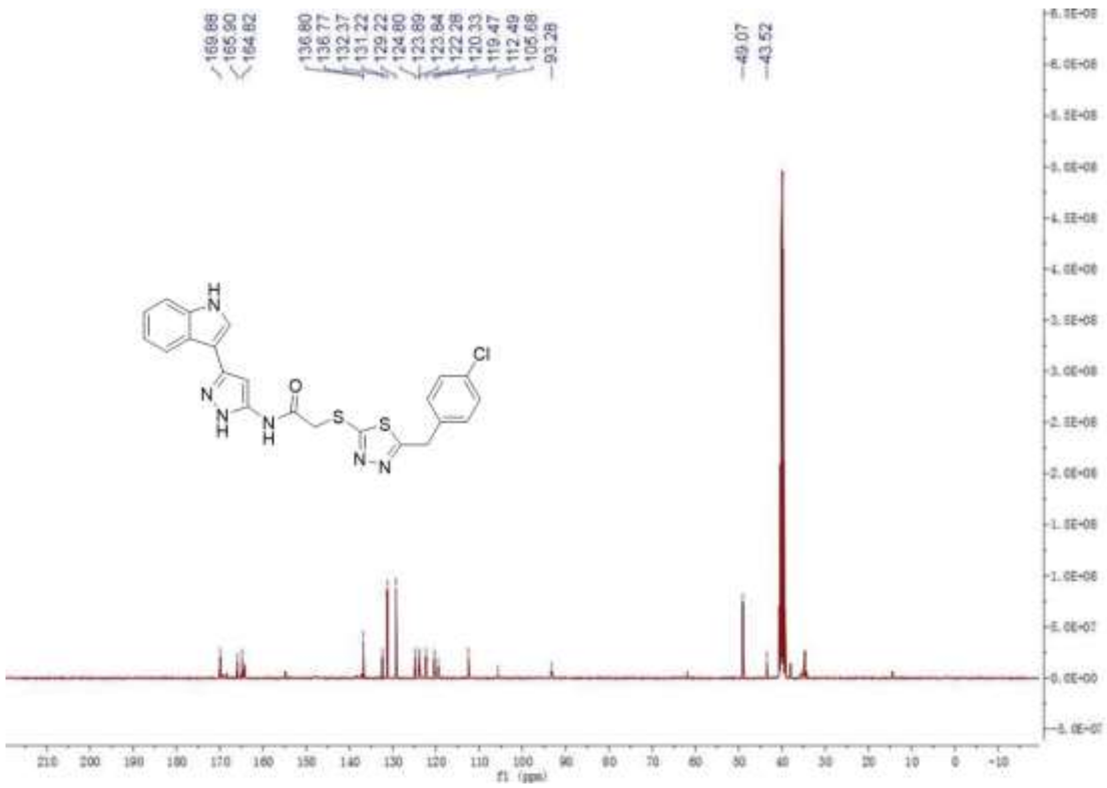

Figure S11. ^{13}**C NMR Spectrum (DMSO-d_6, 126 MHz) of 10d.**

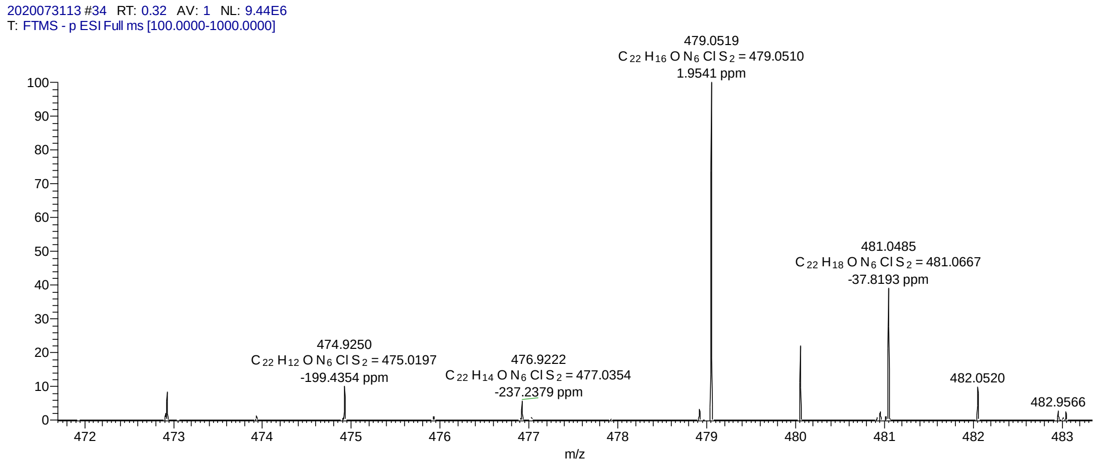

Figure S12. HR-MS Spectrum of 10d.

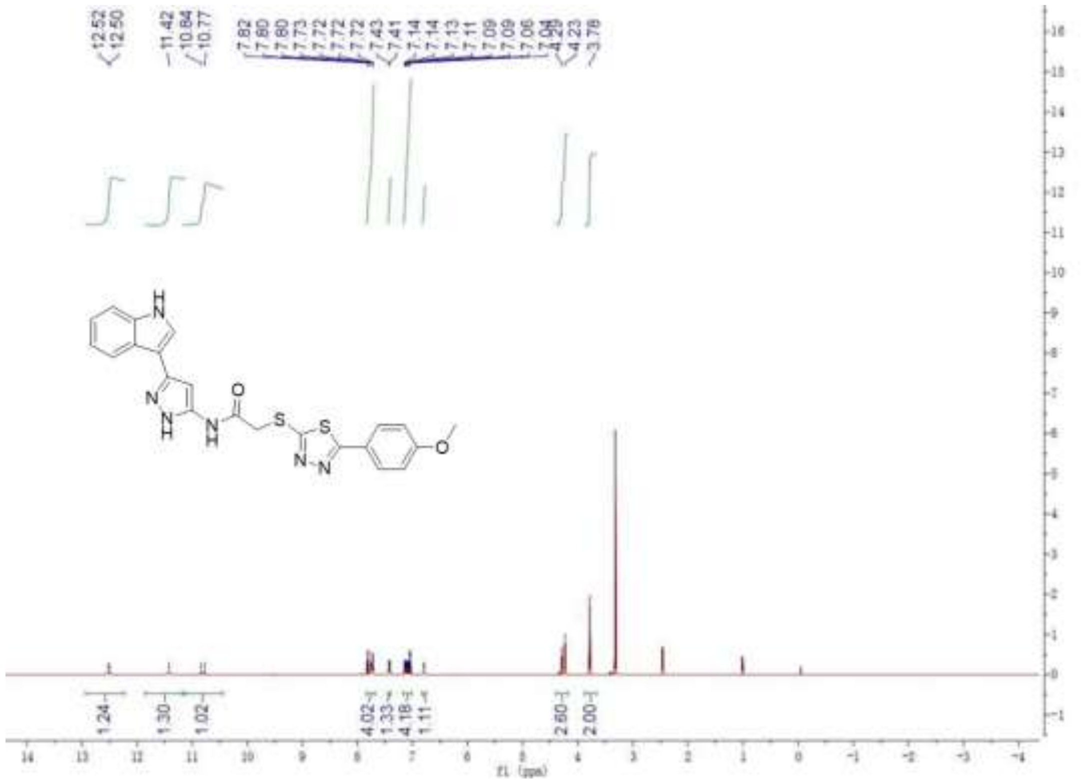

Figure S13. ^{1}H NMR Spectrum (DMSO-d_6, 500 MHz) of 10e.

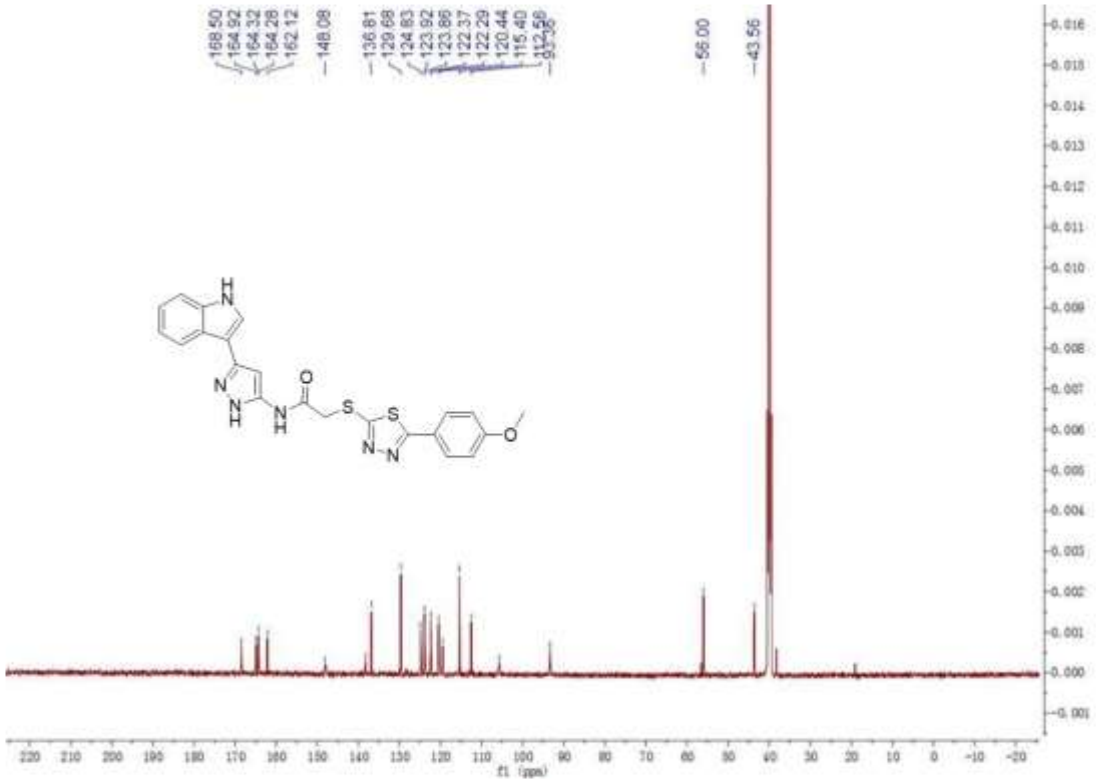

Figure S14. ^{13}C NMR Spectrum (DMSO-d_6, 126 MHz) of 10e.

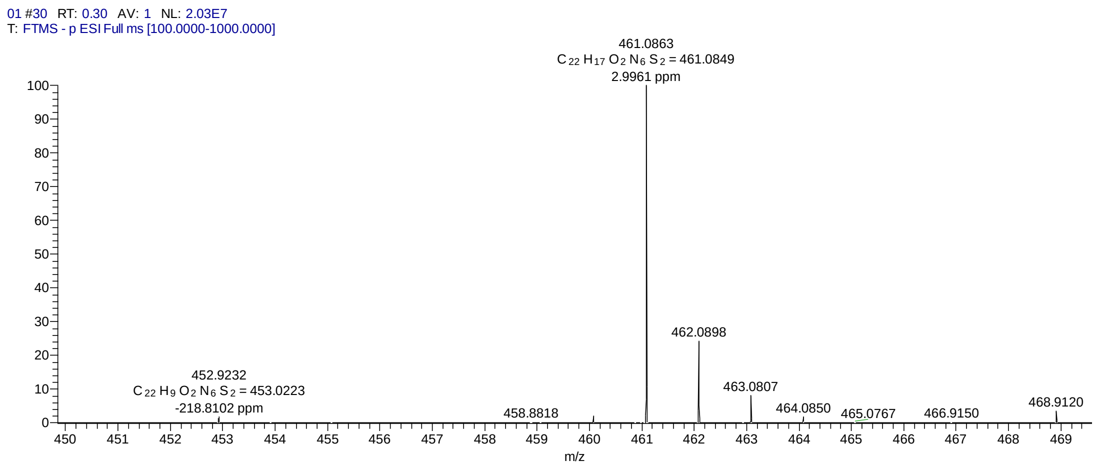

Figure S15. HR-MS Spectrum of 10e.

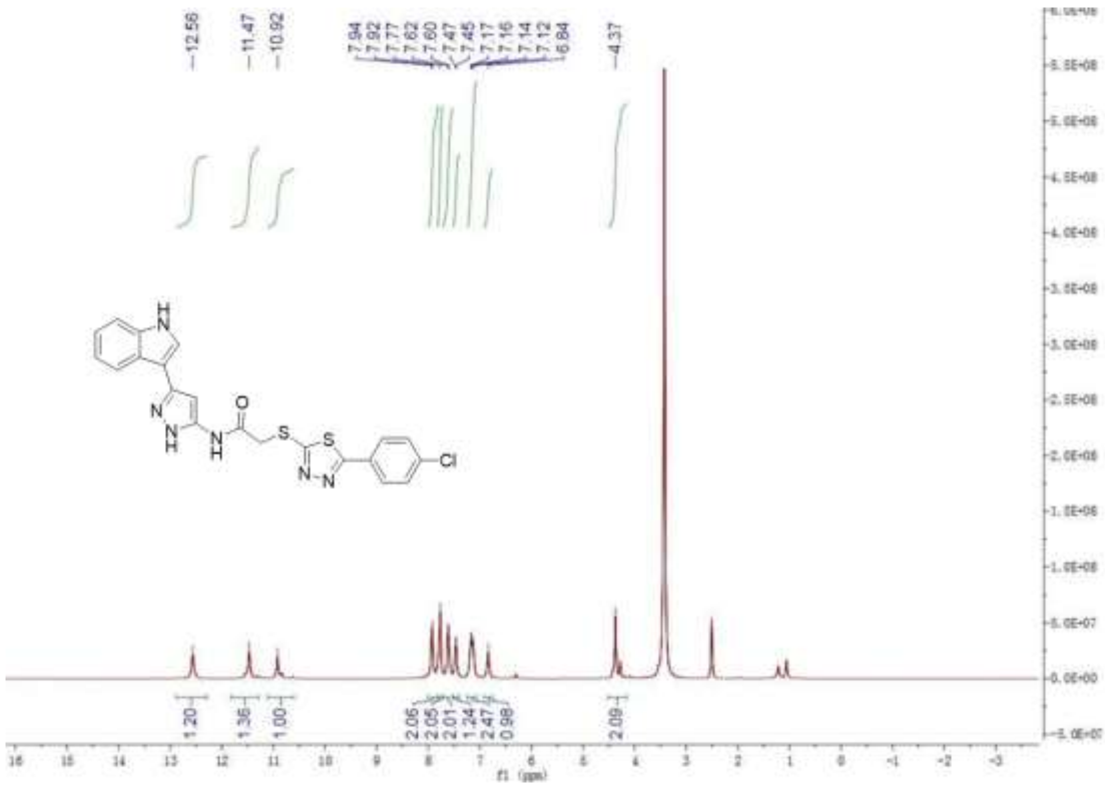

Figure S16. ^{1}H NMR Spectrum (DMSO-d_6, 500 MHz) of 10f.

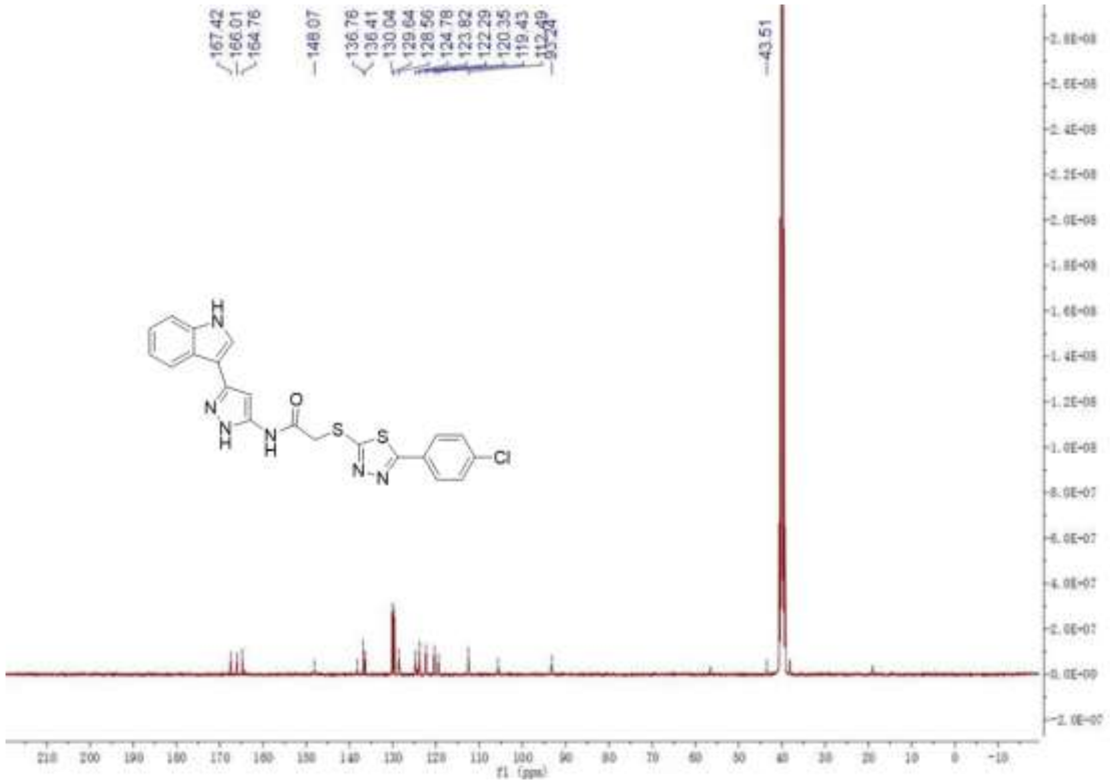

Figure S17. ^{13}**C NMR Spectrum (DMSO-**d_6**, 126 MHz) of 10f.**

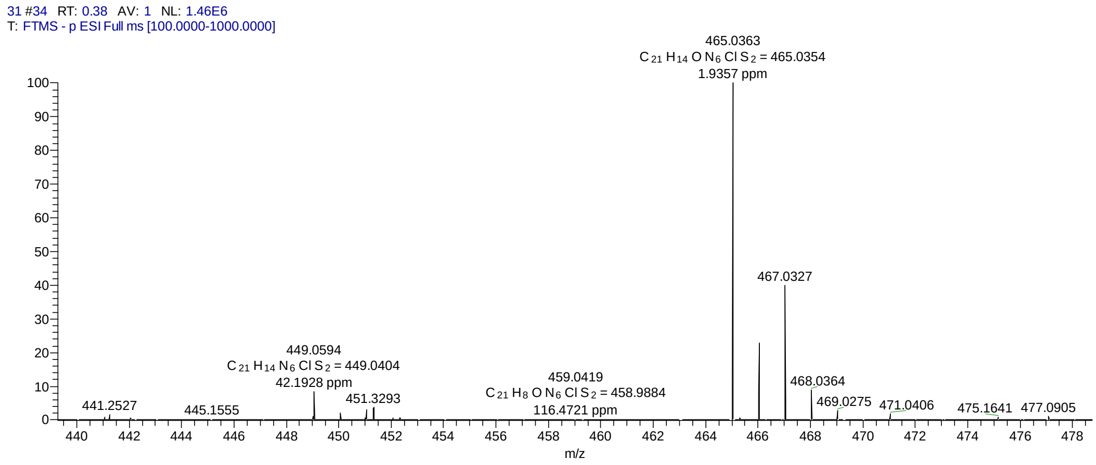

Figure S18. HR-MS Spectrum of 10f.

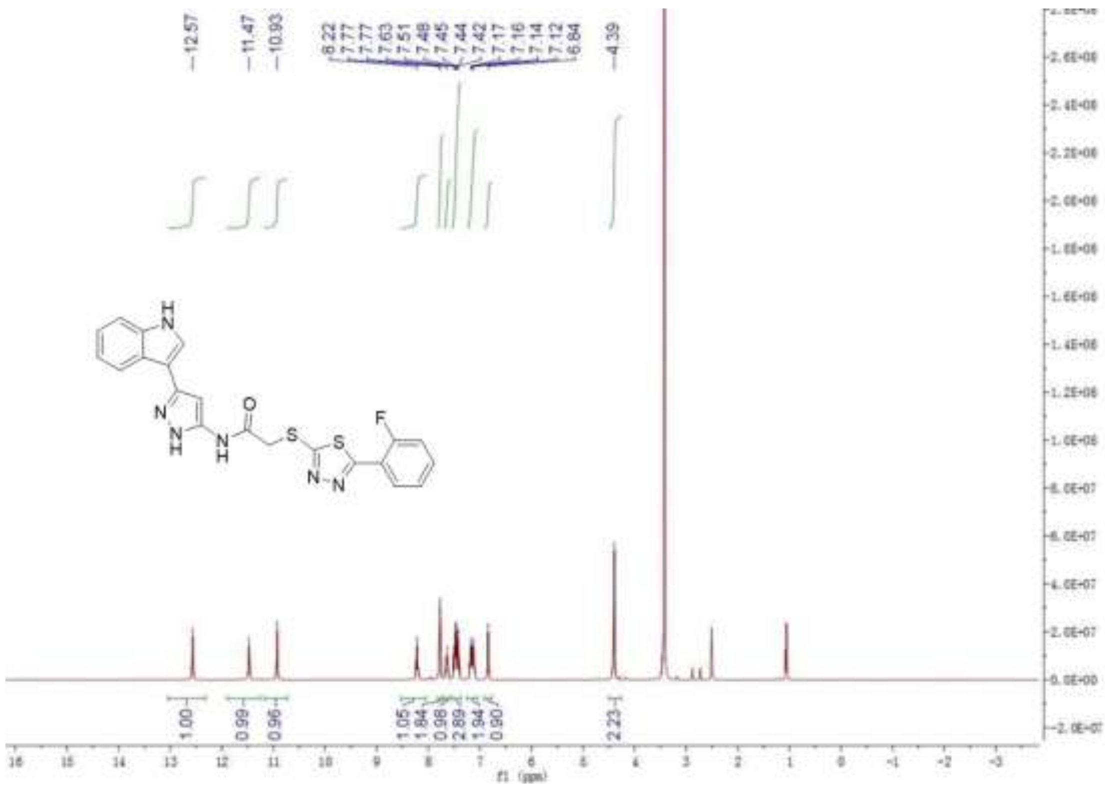

Figure S19. ^{1}**H NMR Spectrum (DMSO-*d*$_6$, 500 MHz) of 10g.**

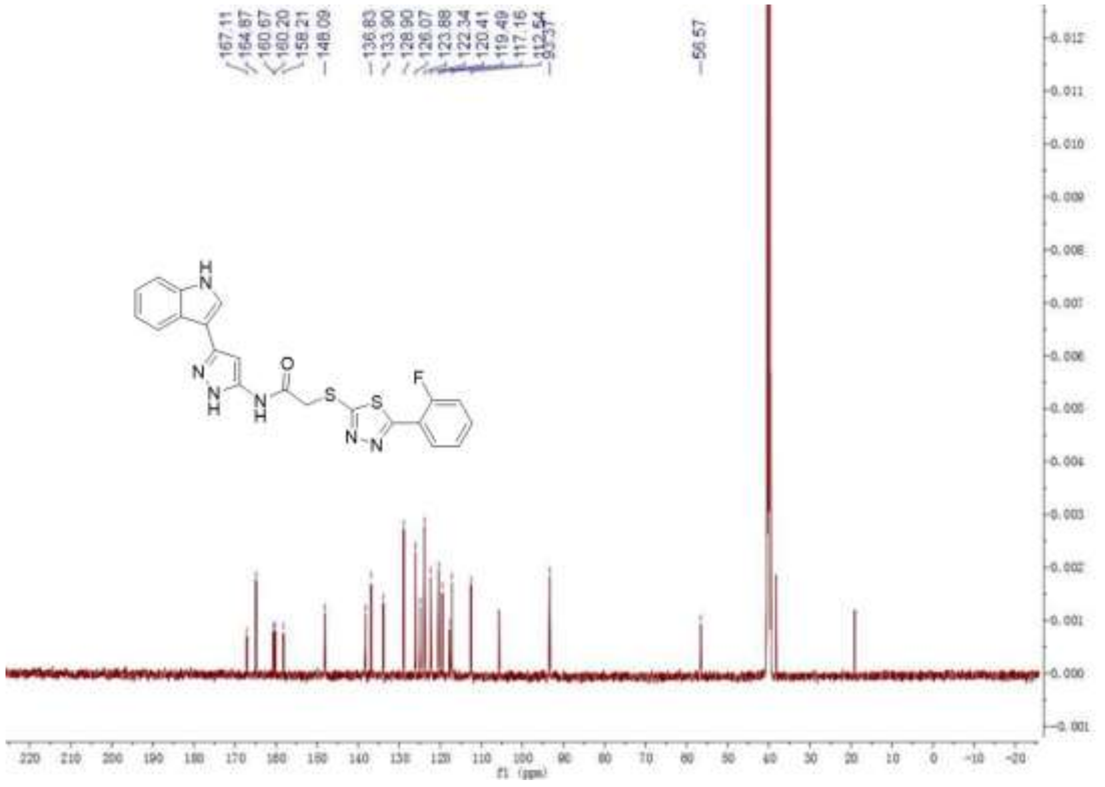

Figure S20. ^{13}**C NMR Spectrum (DMSO-*d*$_6$, 126 MHz) of 10g.**

Information Classification: General

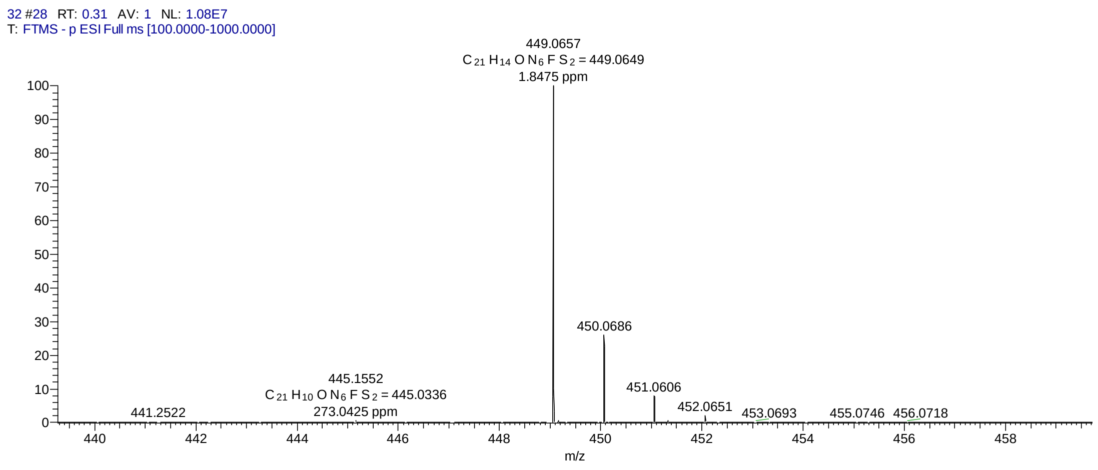

Figure S21. HR-MS Spectrum of 10g.

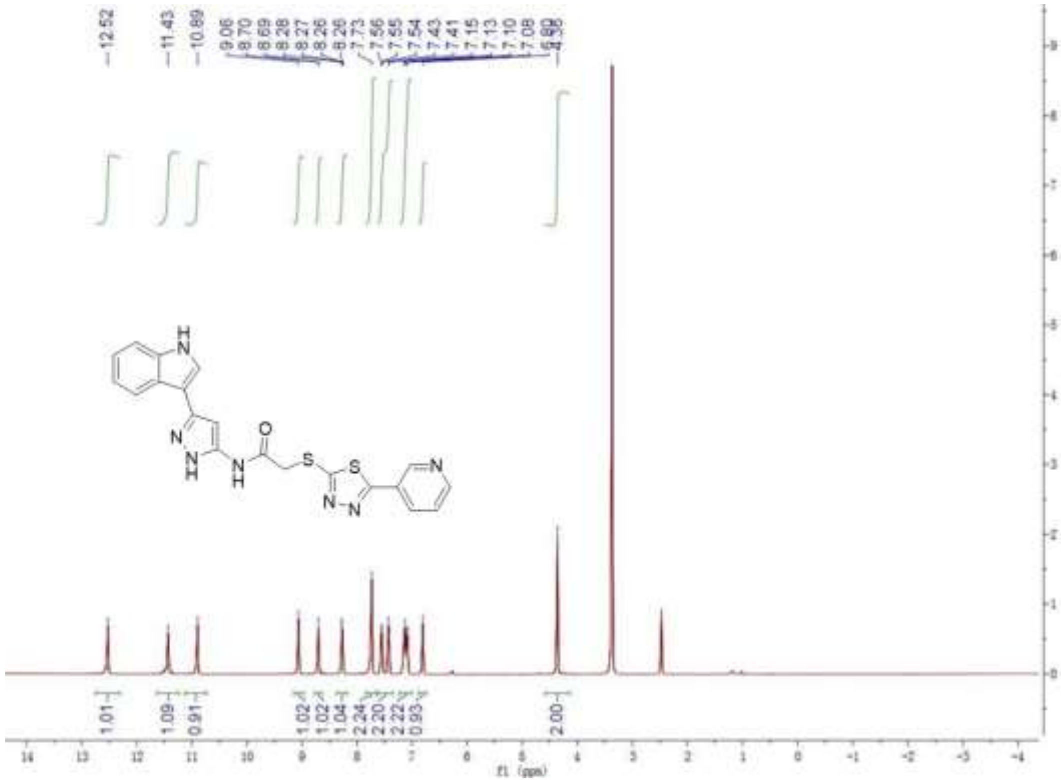

Figure S22. ¹H NMR Spectrum (DMSO-*d₆*, 500 MHz) of 10h.

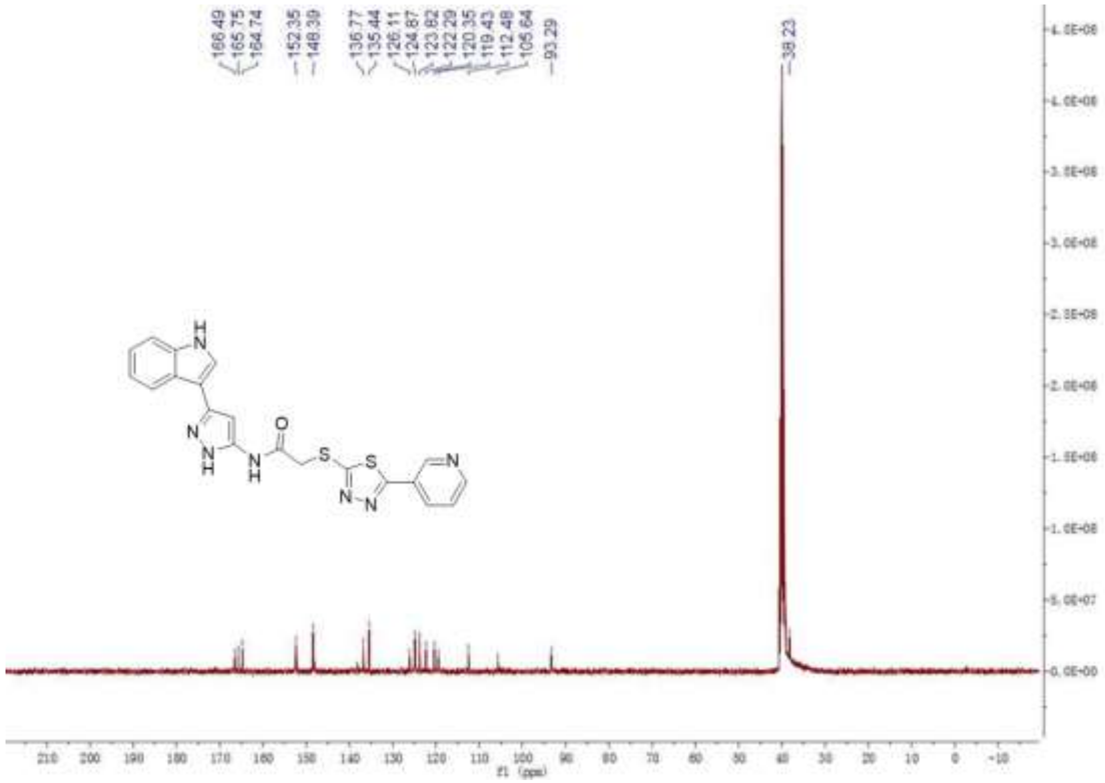

Figure S23. ^{13}C NMR Spectrum (DMSO-d_6, 126 MHz) of 10h.

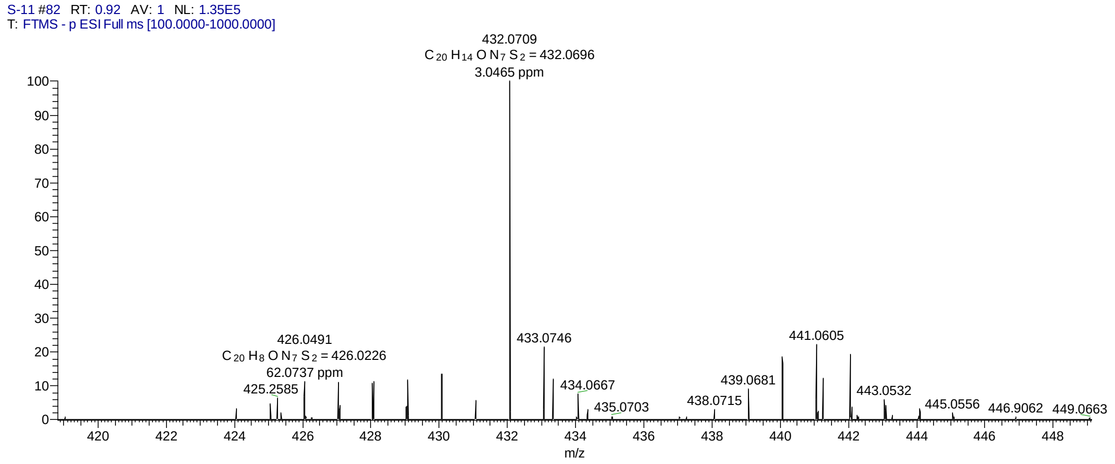

Figure S24. HR-MS Spectrum of 10h.

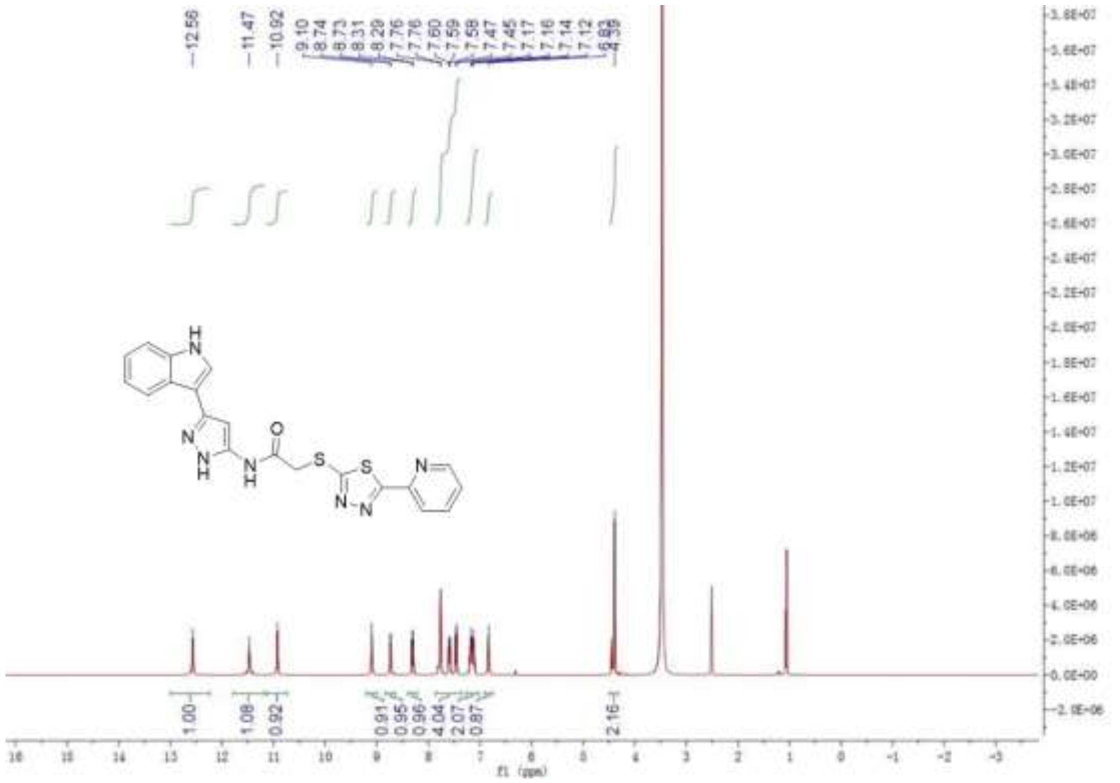

Figure S25. ^{1}H NMR Spectrum (DMSO-d_6, 500 MHz) of 10i.

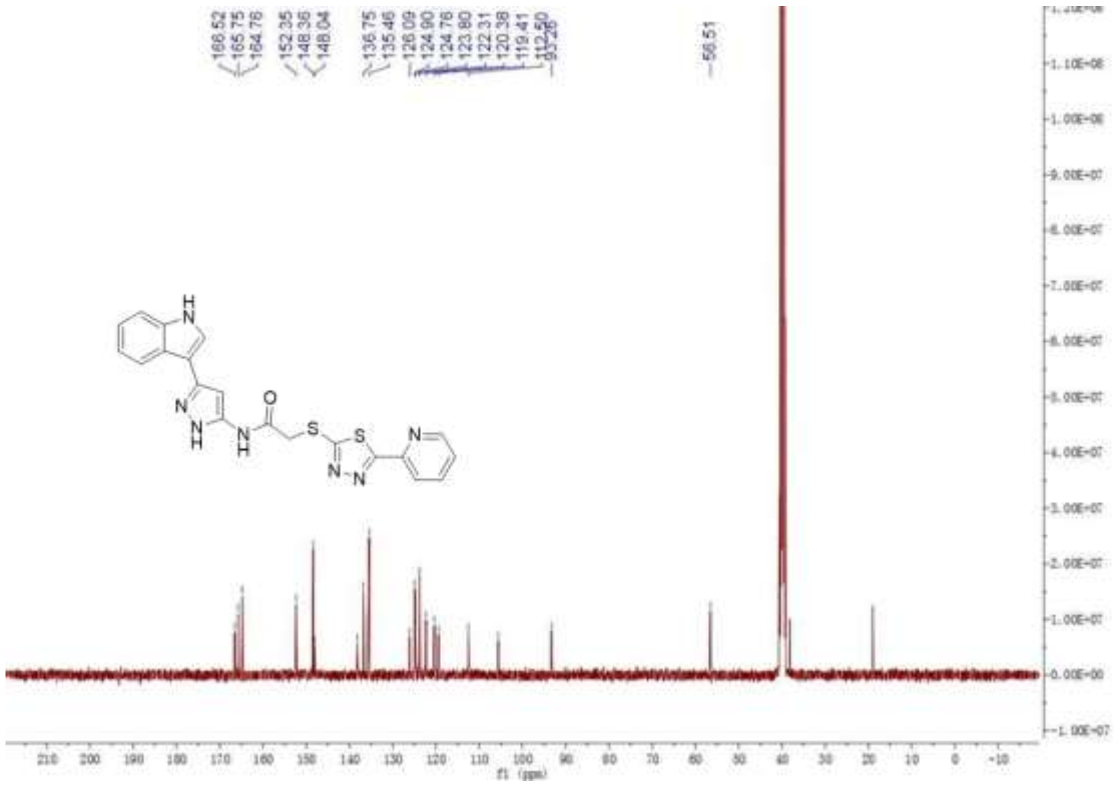

Figure S26. ^{13}C NMR Spectrum (DMSO-d_6, 126 MHz) of 10i.

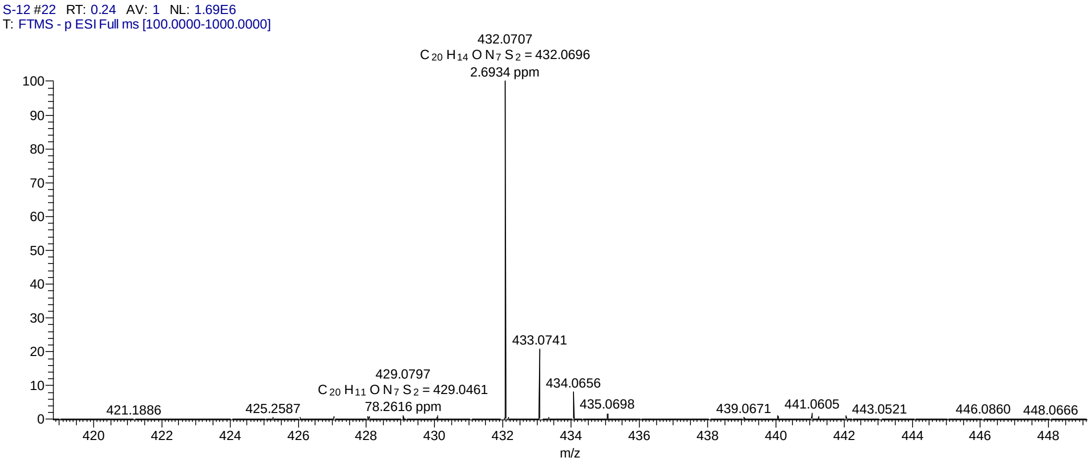

Figure S27. HR-MS Spectrum of 10i.

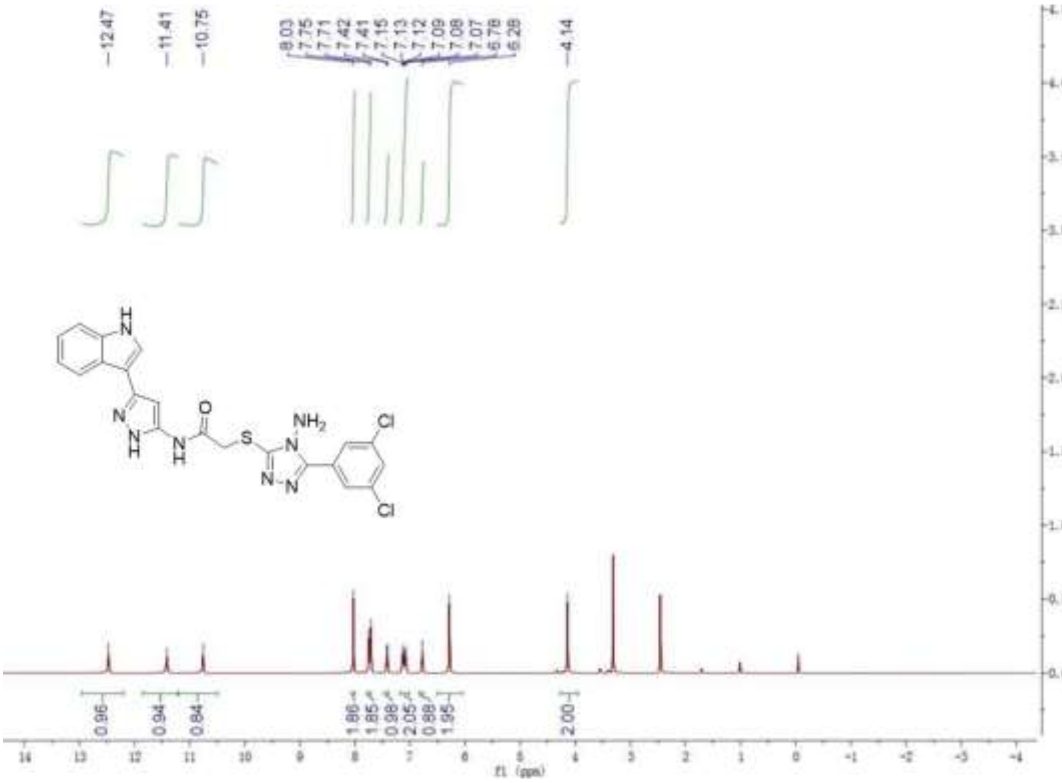

Figure S28. ^{1}H NMR Spectrum (DMSO-d_6, 500 MHz) of 11a.

Information Classification: General

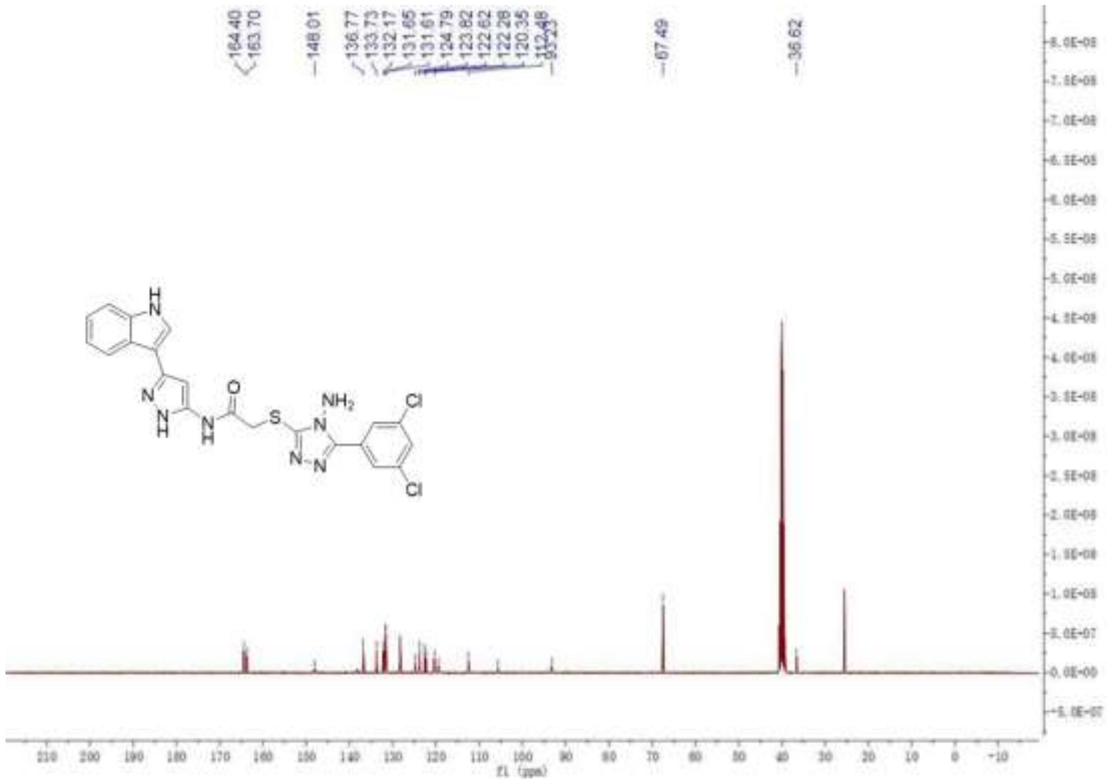

Figure S29. ^{13}C NMR Spectrum (DMSO-d_6, 126 MHz) of 11a.

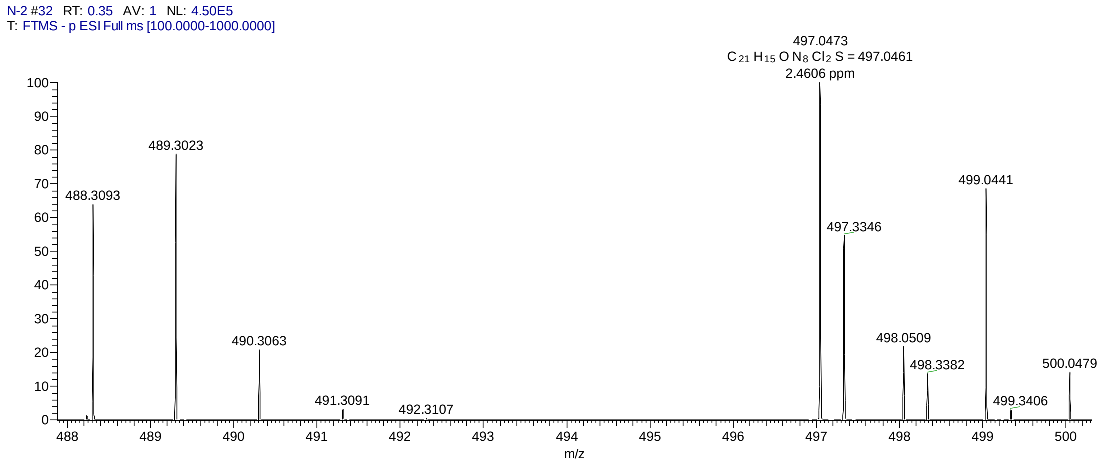

Figure S30. HR-MS Spectrum of 11a.

Information Classification: General

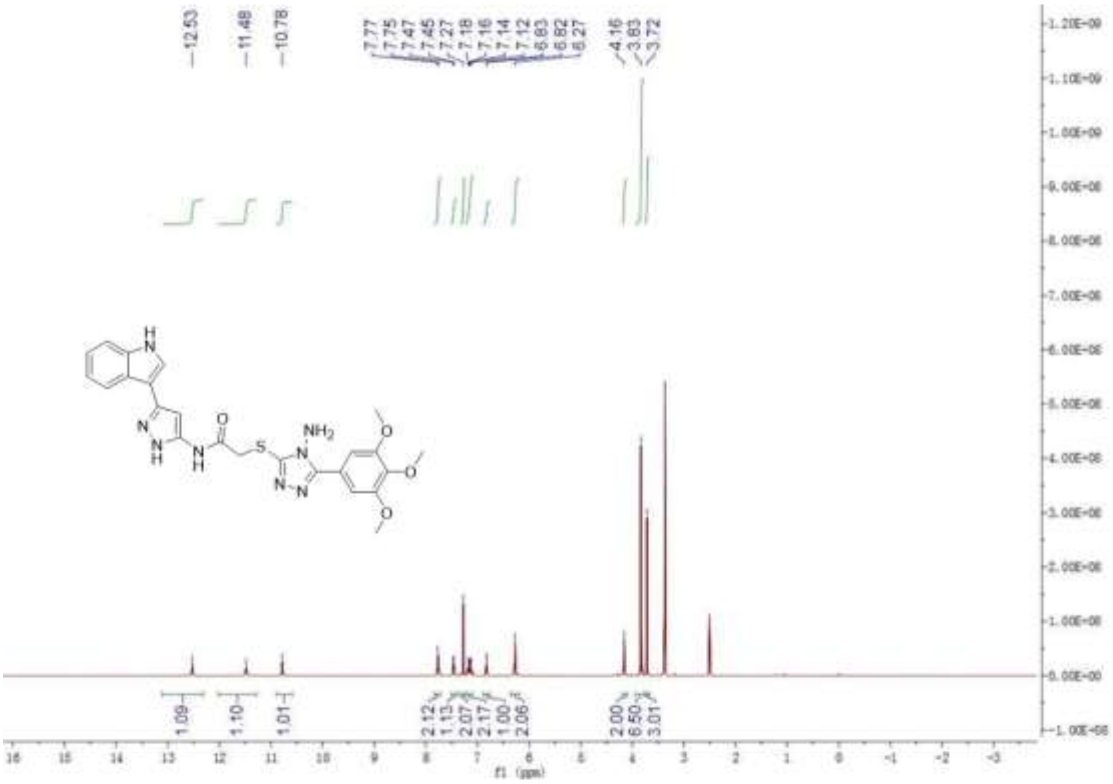

Figure S31. ^{1}**H NMR Spectrum (DMSO-**d_6**, 500 MHz) of 11b.**

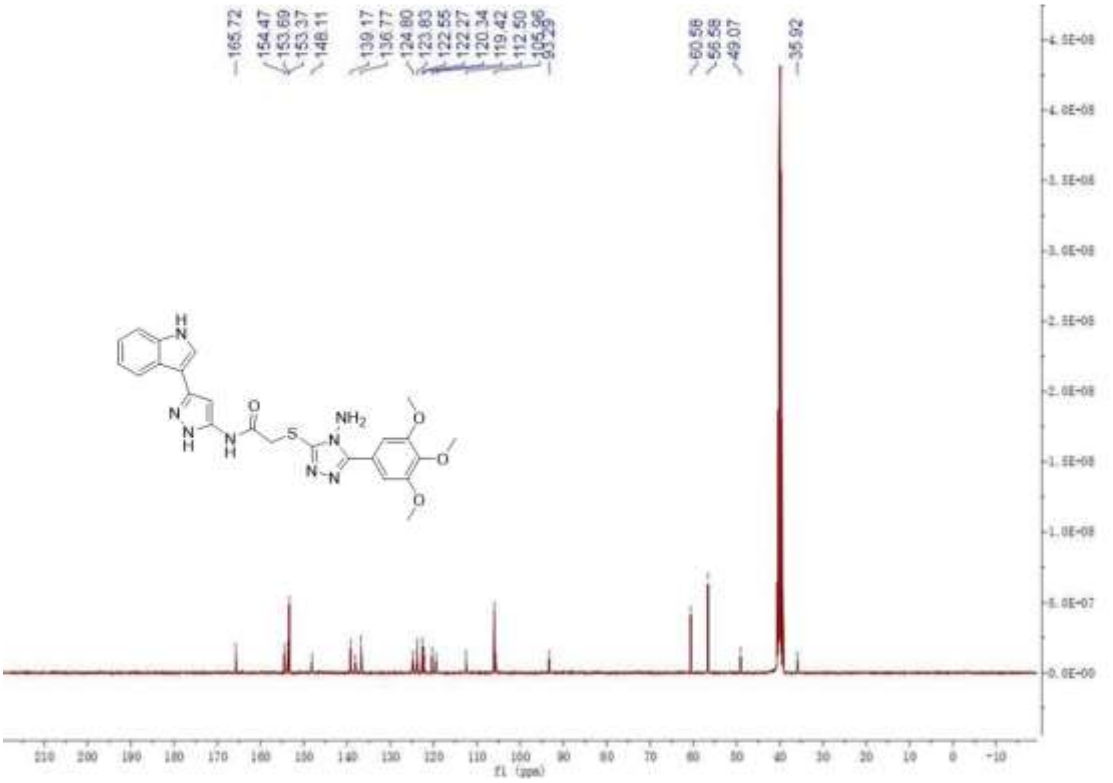

Figure S32. ^{13}**C NMR Spectrum (DMSO-**d_6**, 126 MHz) of 11b.**

Information Classification: General

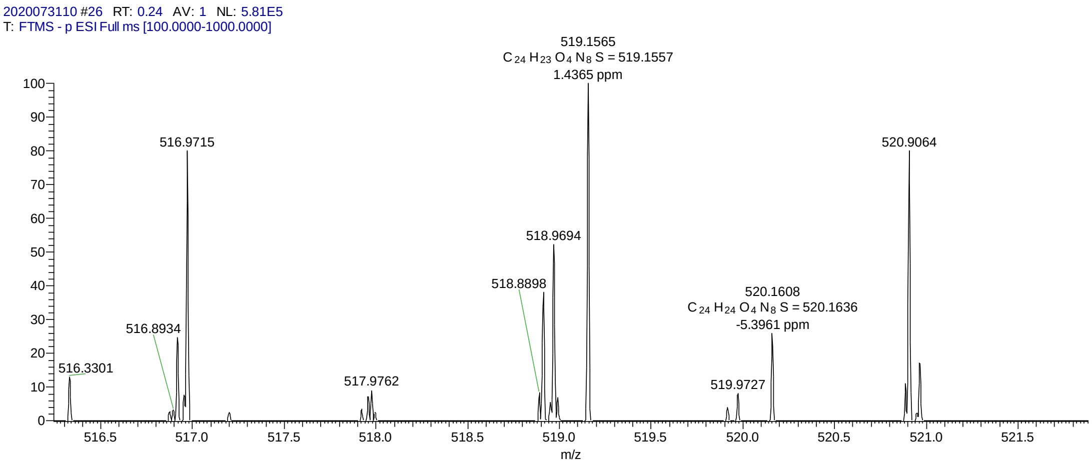

Figure S33. HR-MS Spectrum of 11b.

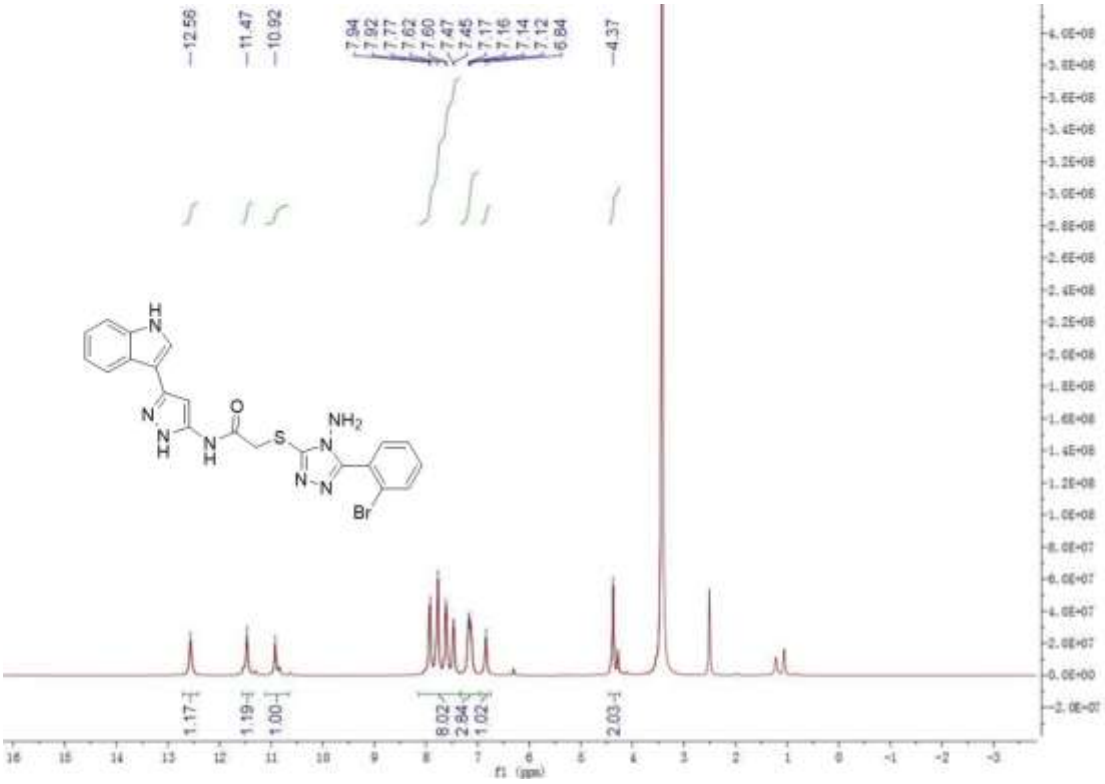

Figure S34. ^{1}H NMR Spectrum (DMSO-d_6, 500 MHz) of 11c.

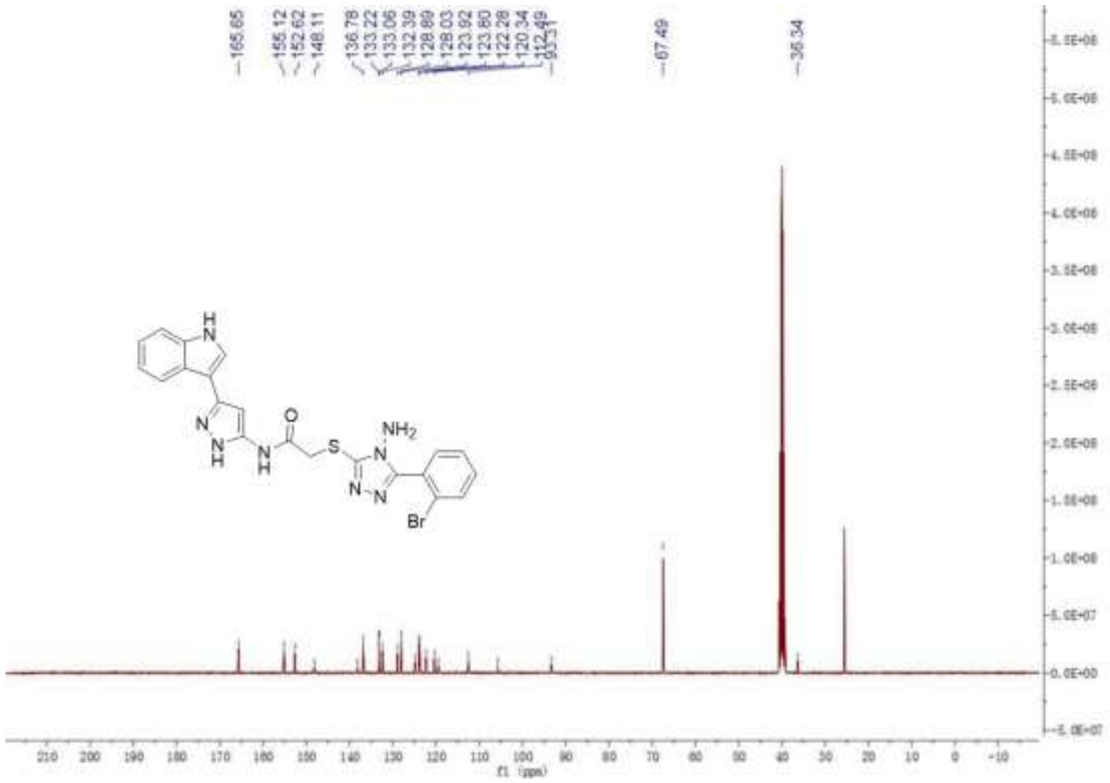

Figure S35. **^{13}C NMR Spectrum (DMSO-d_6, 126 MHz) of 11c.**

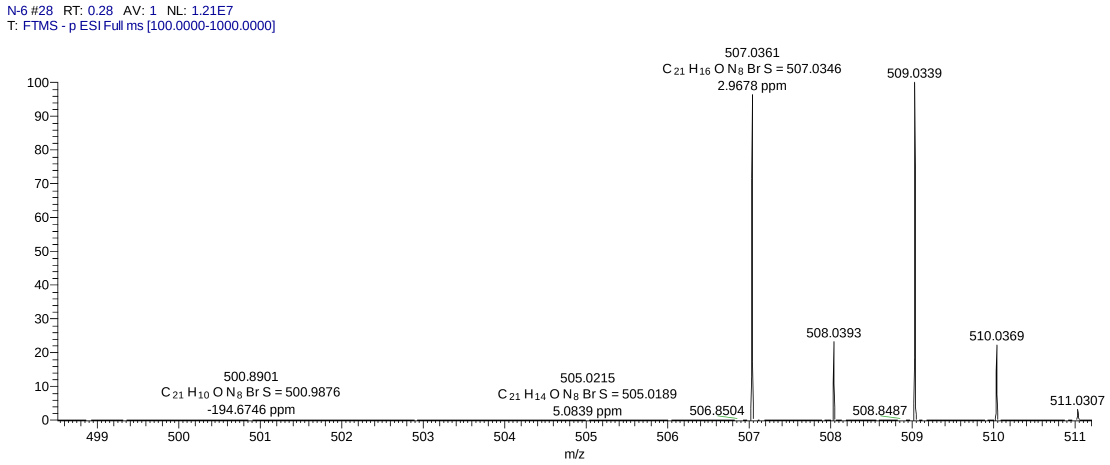

Figure S36. HR-MS Spectrum of 11c.

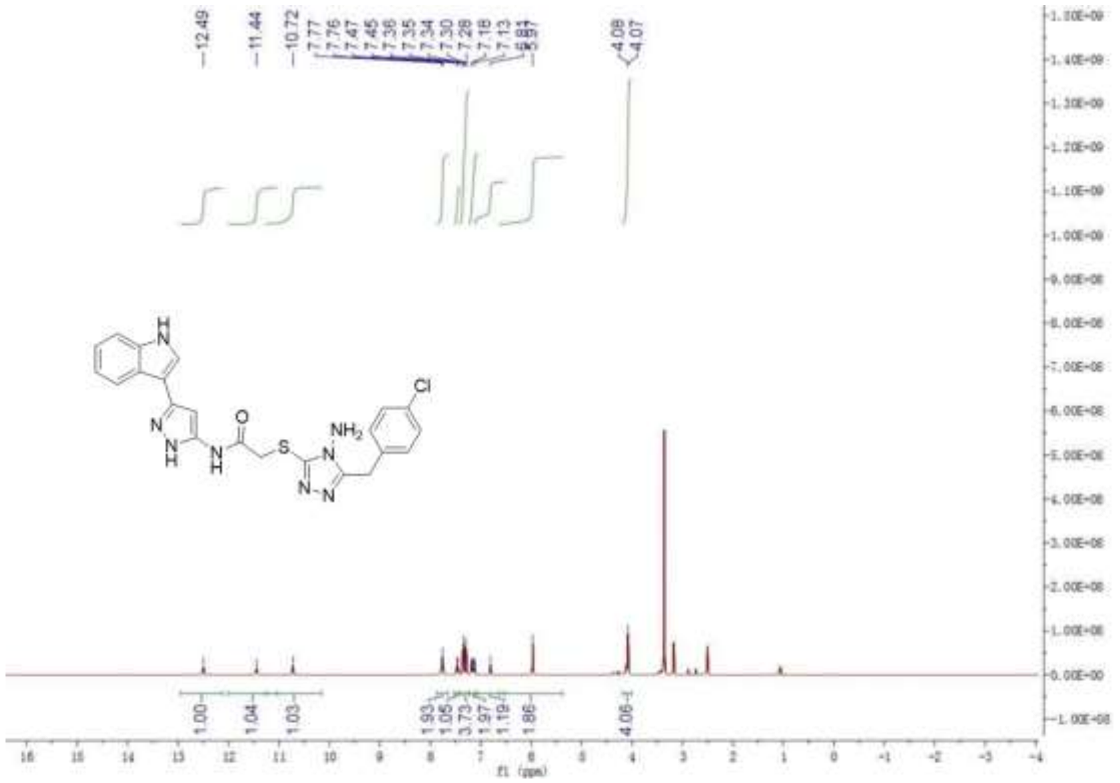

Figure S37. ^{1}H NMR Spectrum (DMSO-d_6, 500 MHz) of 11d.

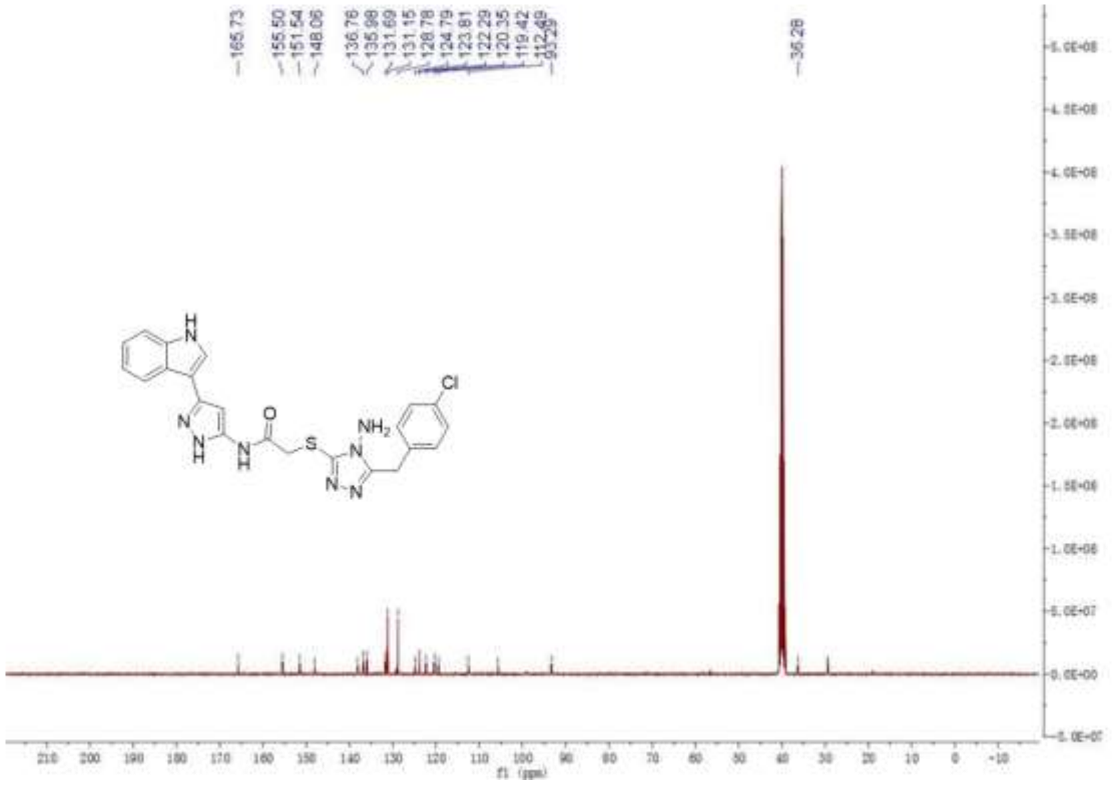

Figure S38. ^{13}C NMR Spectrum (DMSO-d_6, 126 MHz) of 11d.

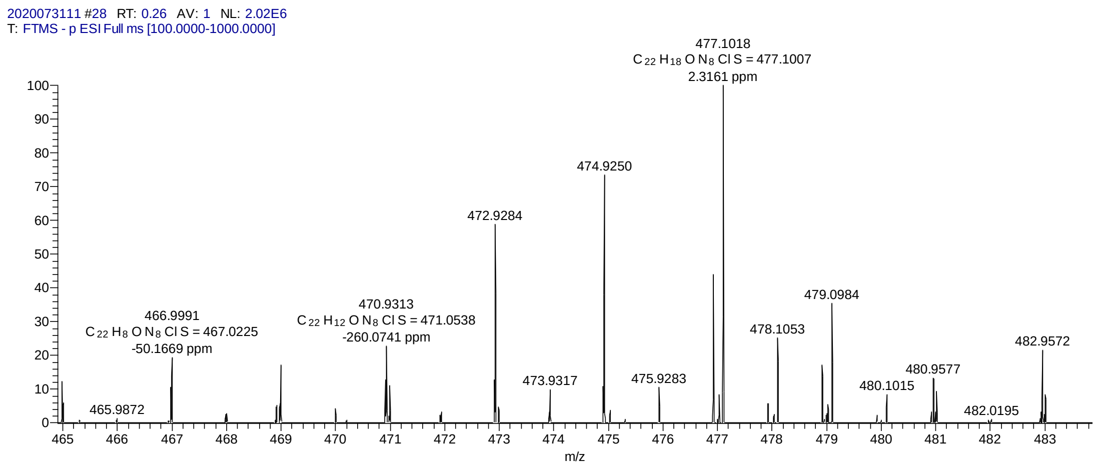

Figure S39. HR-MS Spectrum of 11d.

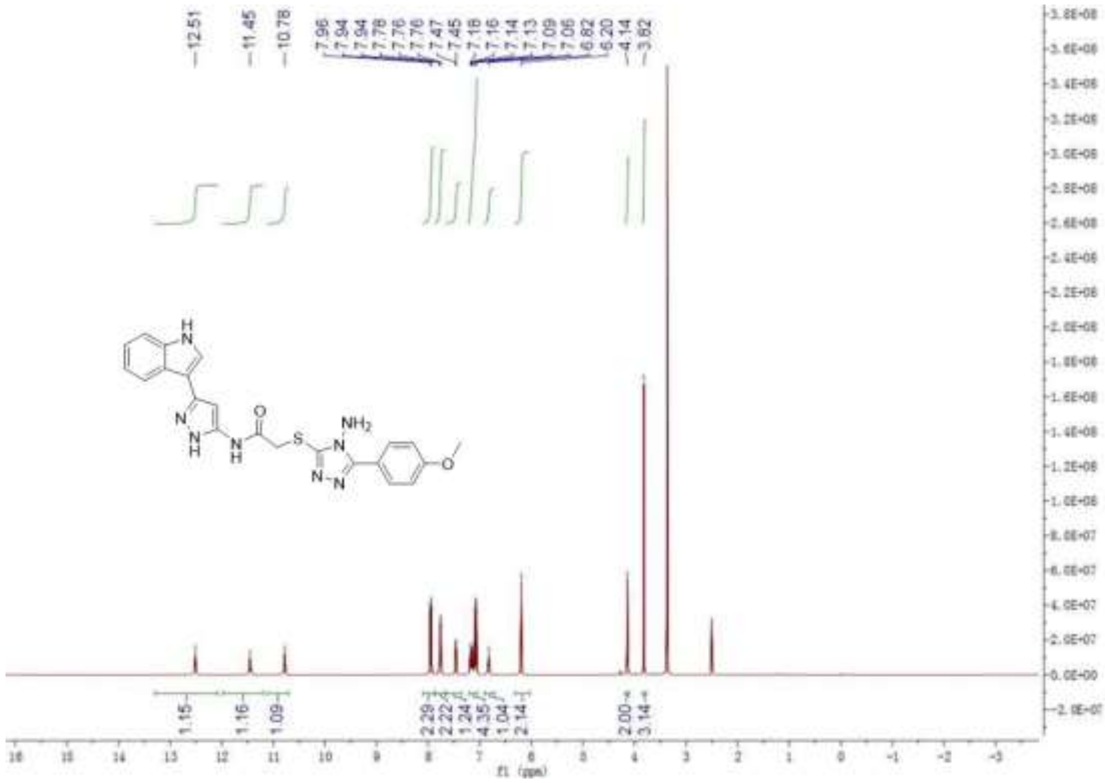

Figure S40. ^{1}H NMR Spectrum (DMSO-d_6, 500 MHz) of 11e.

Information Classification: General

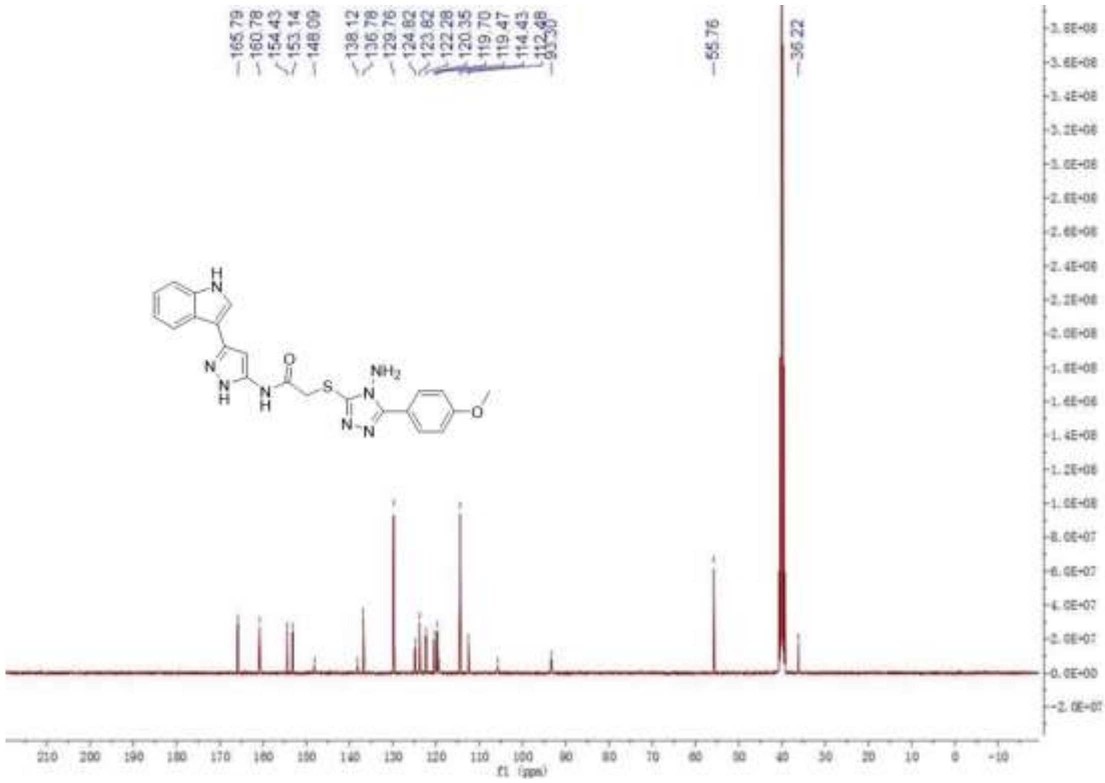

Figure S41. ^{13}**C NMR Spectrum (DMSO-*d*$_6$, 126 MHz) of 11e.**

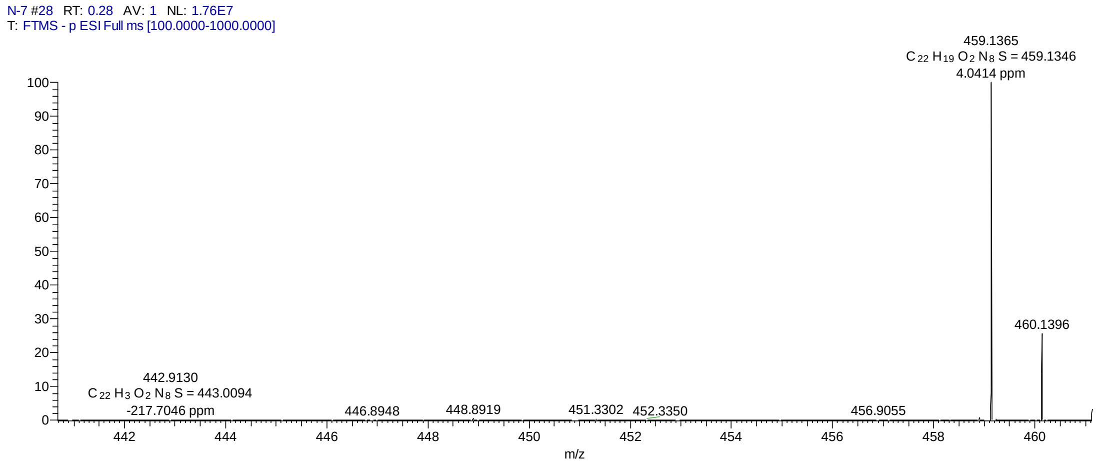

Figure S42. HR-MS Spectrum of 11e.

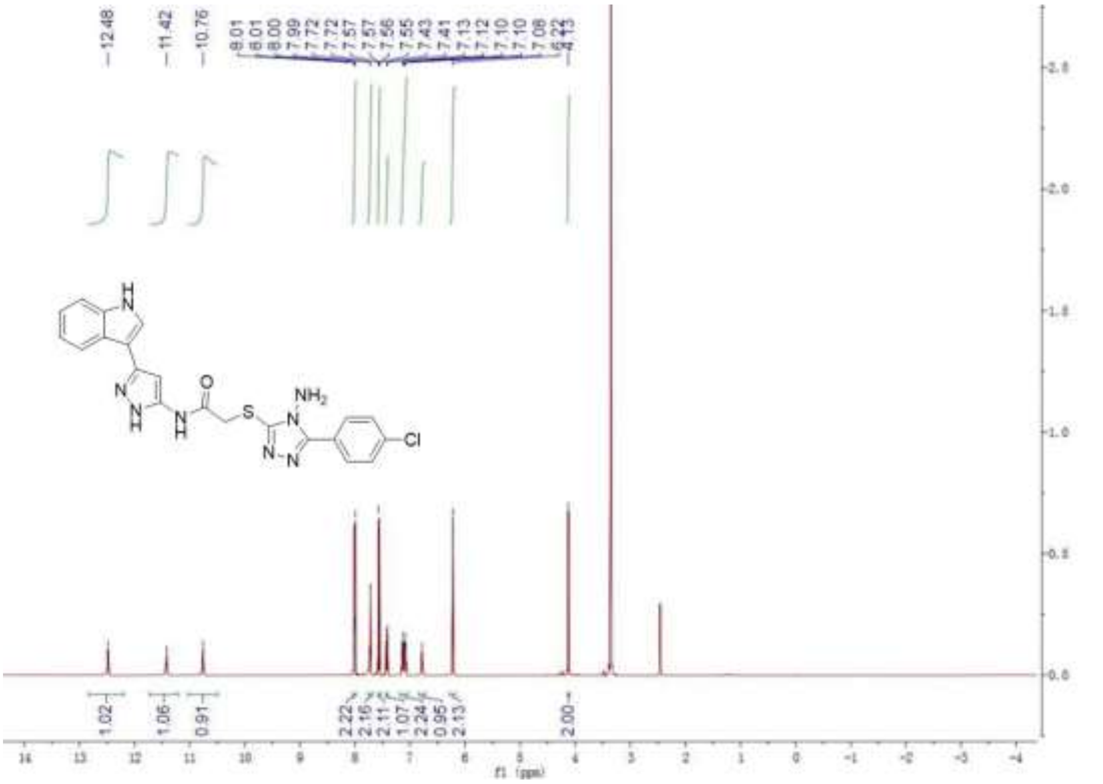

Figure S43. ^{1}**H NMR Spectrum (DMSO-*d*$_6$, 500 MHz) of 11f.**

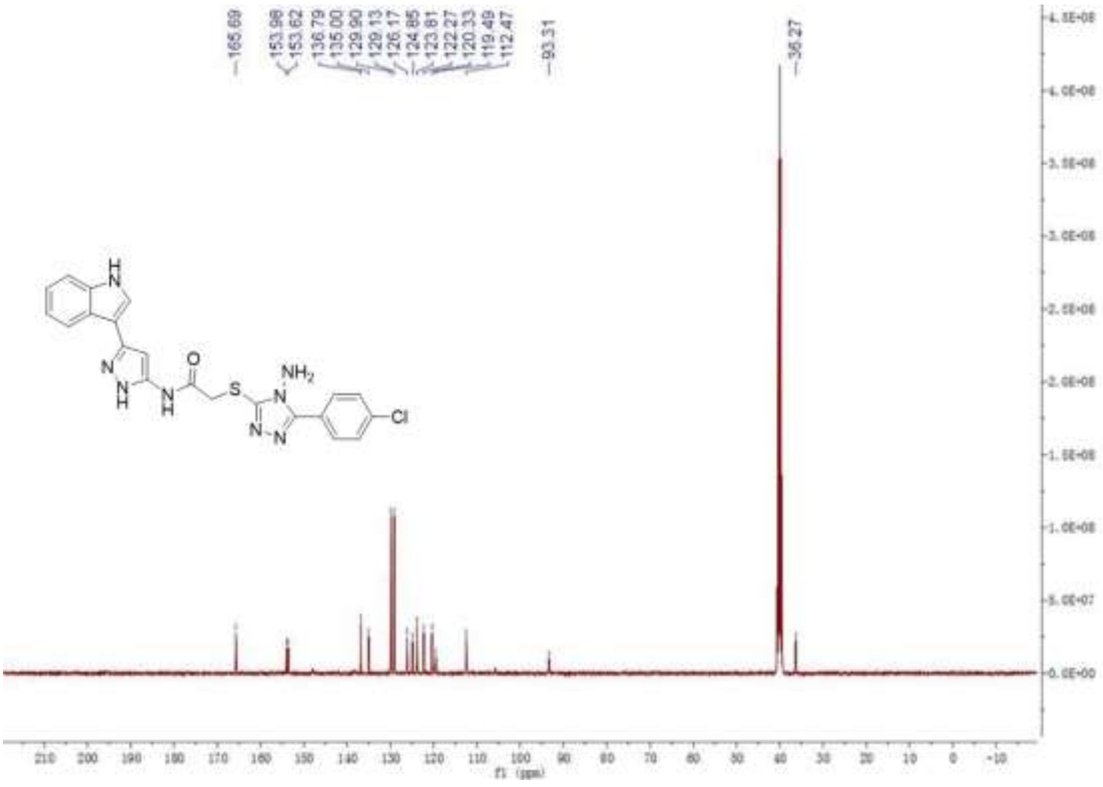

Figure S44. ^{13}**C NMR Spectrum (DMSO-*d*$_6$, 126 MHz) of 11f.**

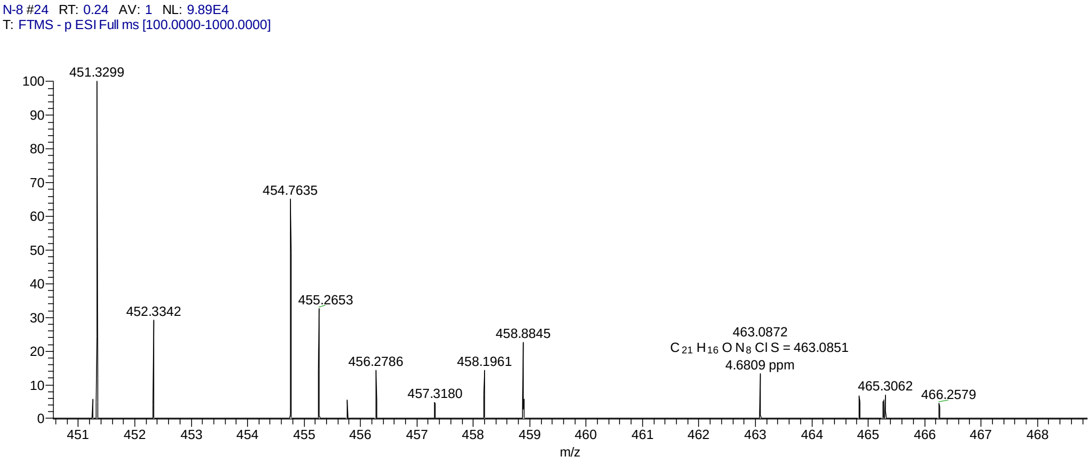

Figure S45. HR-MS Spectrum of 11f.

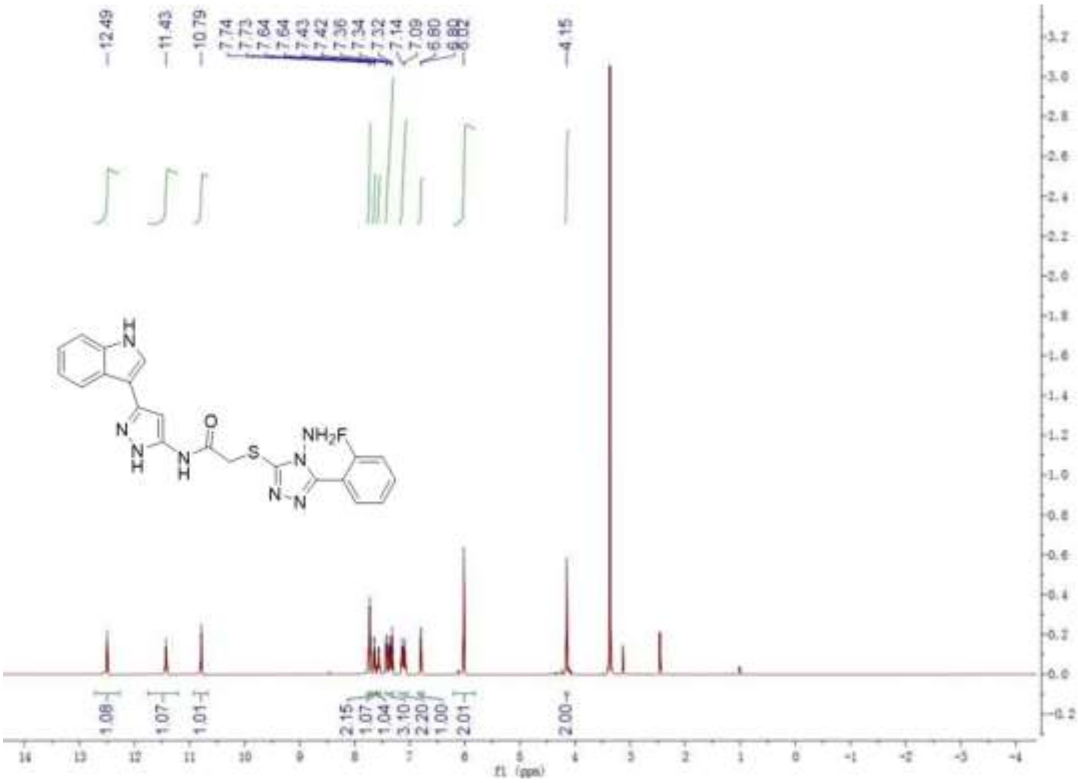

Figure S46. ^{1}H NMR Spectrum (DMSO-d_6, 500 MHz) of 11g.

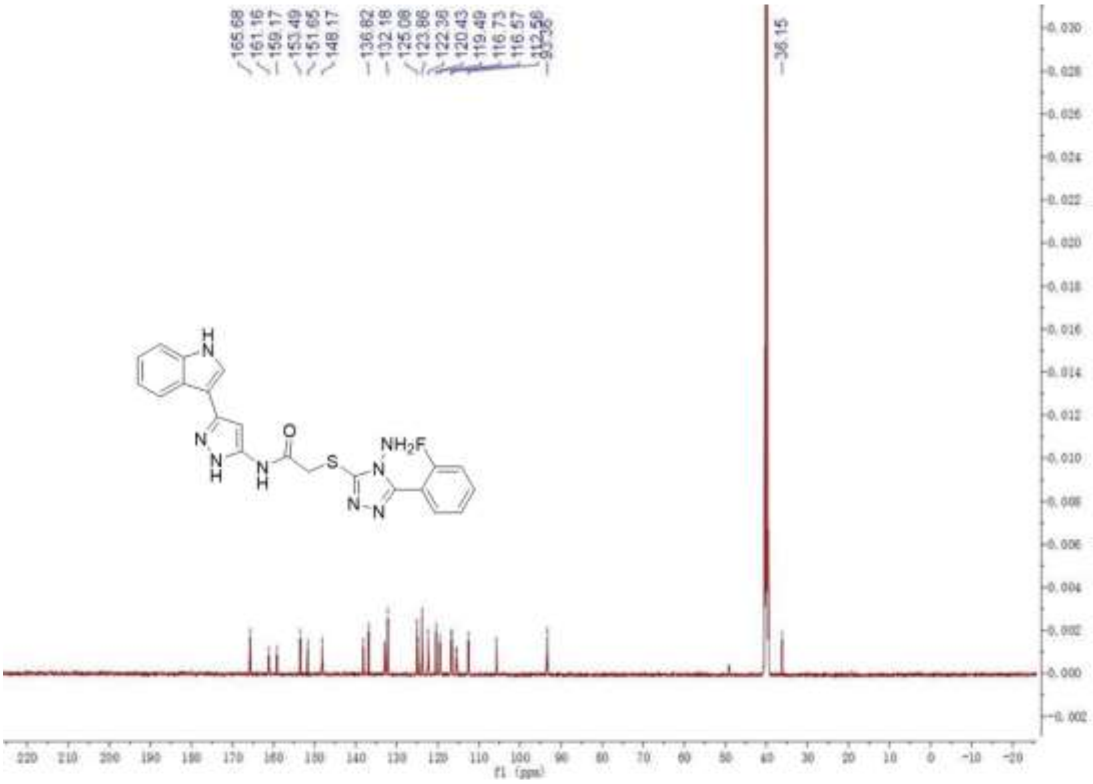

Figure S47. ^{13}C NMR Spectrum (DMSO-d_6, 126 MHz) of 11g.

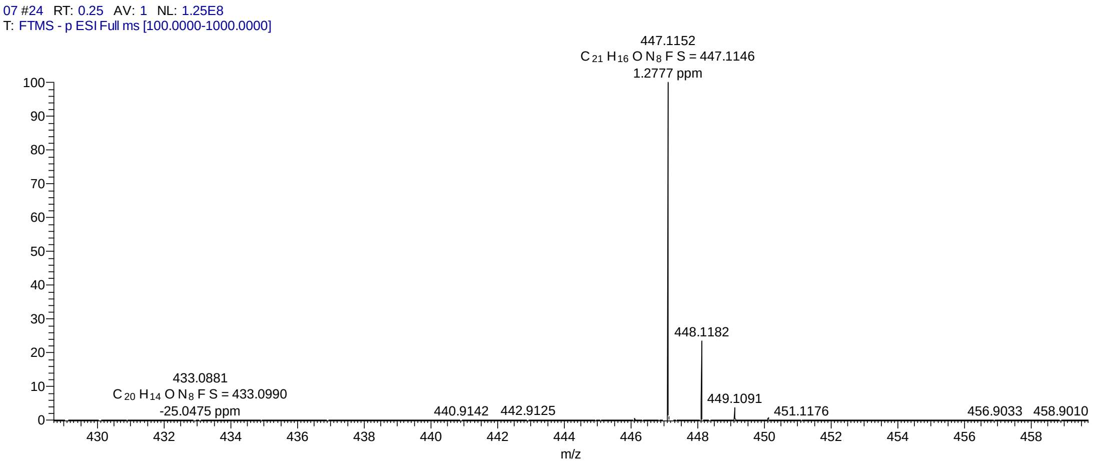

Figure S48. HR-MS Spectrum of 11g.

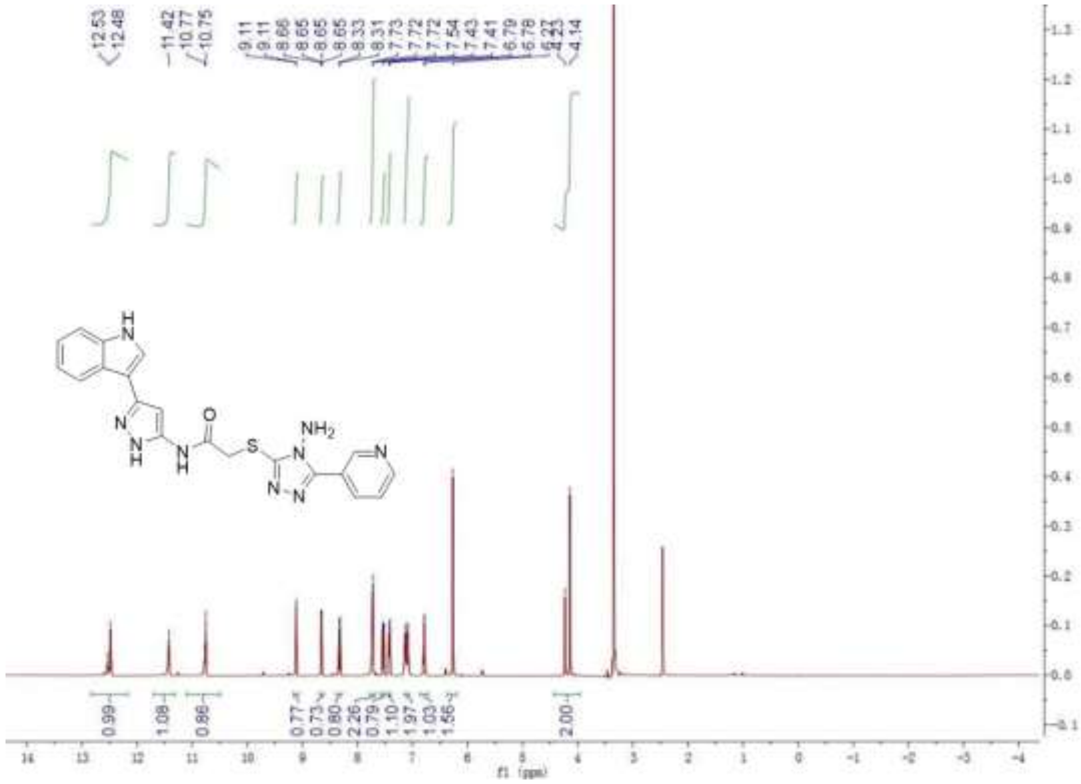

Figure S49. ^{1}H **NMR Spectrum (DMSO-d_6, 500 MHz) of 11h.**

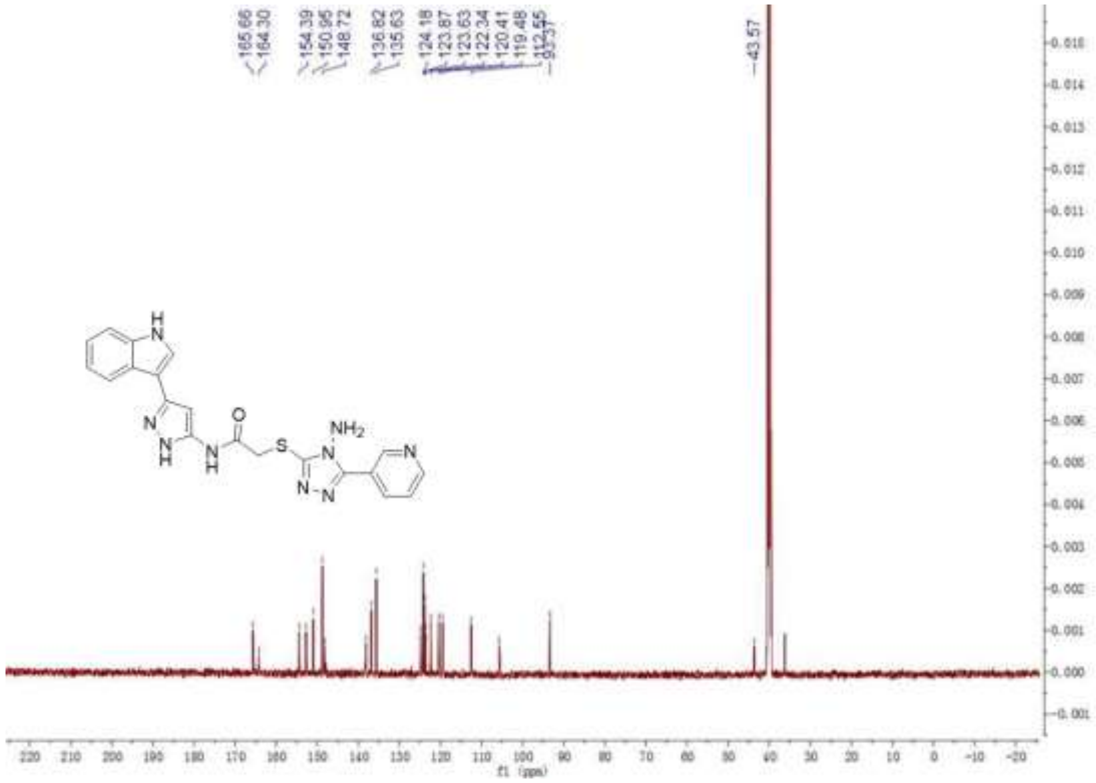

Figure S50. ^{13}C **NMR Spectrum (DMSO-d_6, 126 MHz) of 11h.**

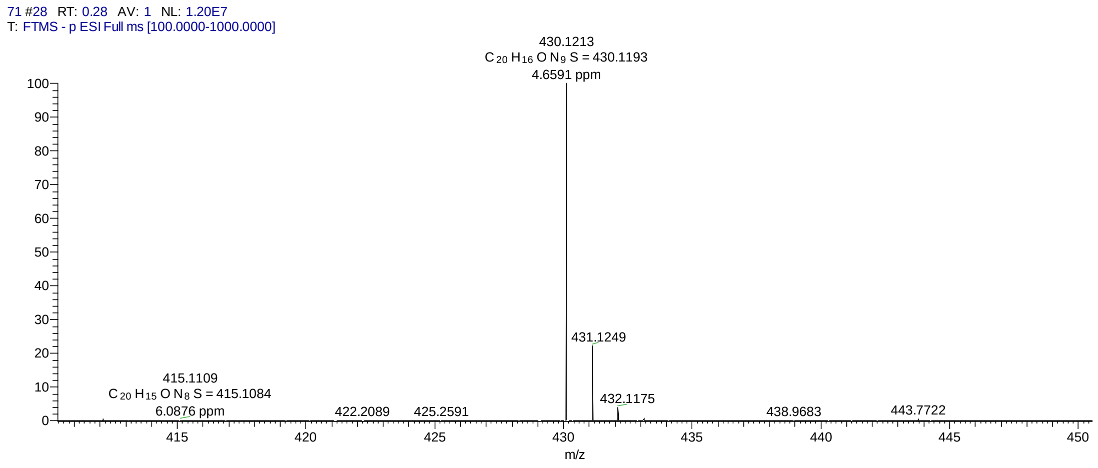

Figure S51. HR-MS Spectrum of 11h.

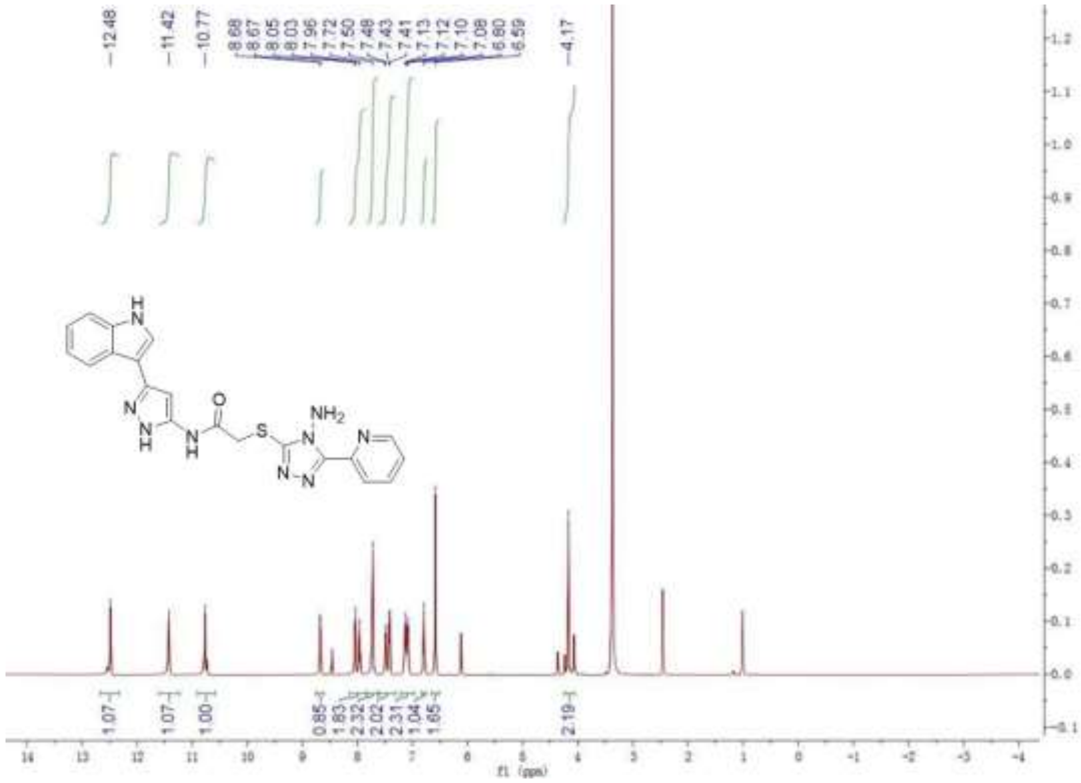

Figure S52. ^{1}H NMR Spectrum (DMSO-d_6, 500 MHz) of 11i.

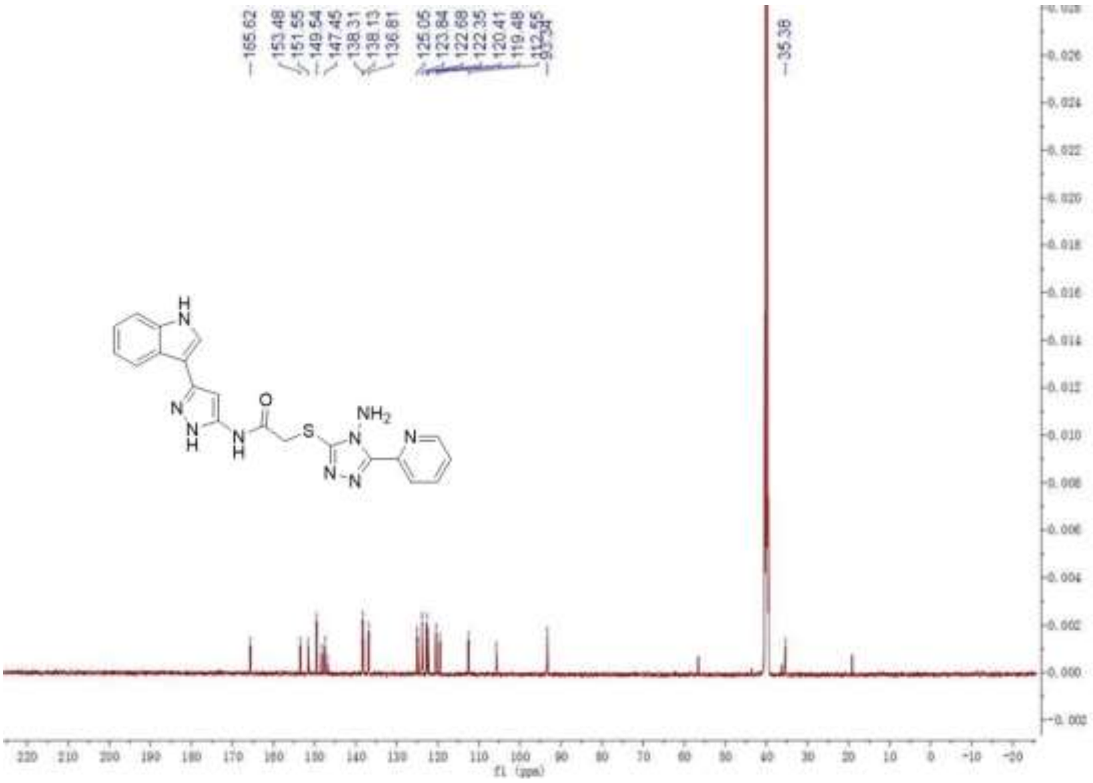

Figure S53. ^{13}C **NMR Spectrum (DMSO-d_6, 126 MHz) of 11i.**

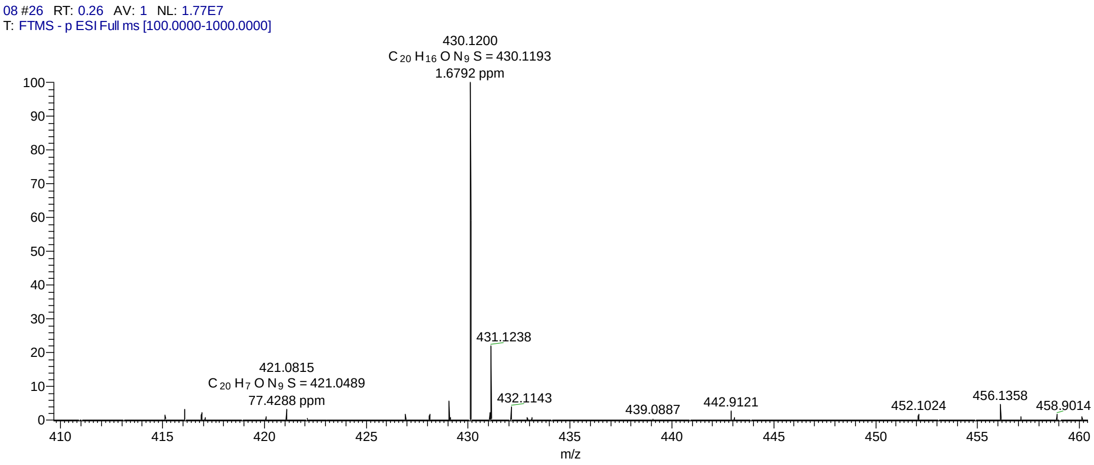

Figure S54. HR-MS Spectrum of 11i.

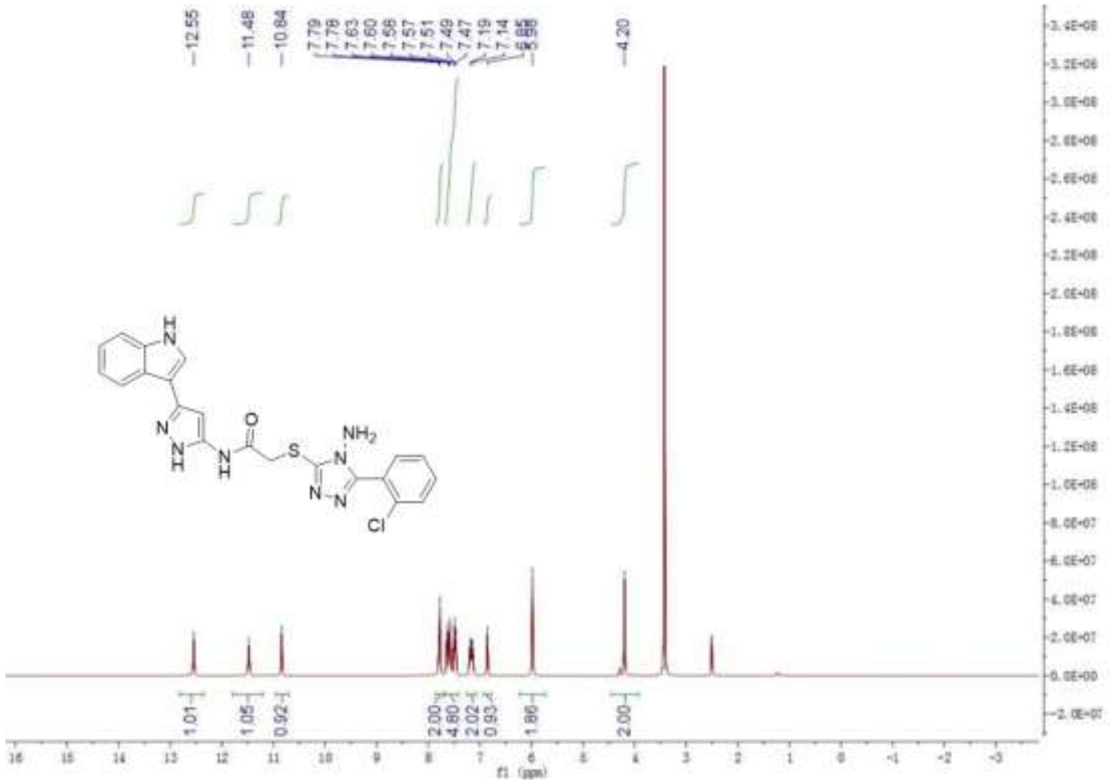

Figure S55. ^{1}**H NMR Spectrum (DMSO-**d_6**, 500 MHz) of 11j.**

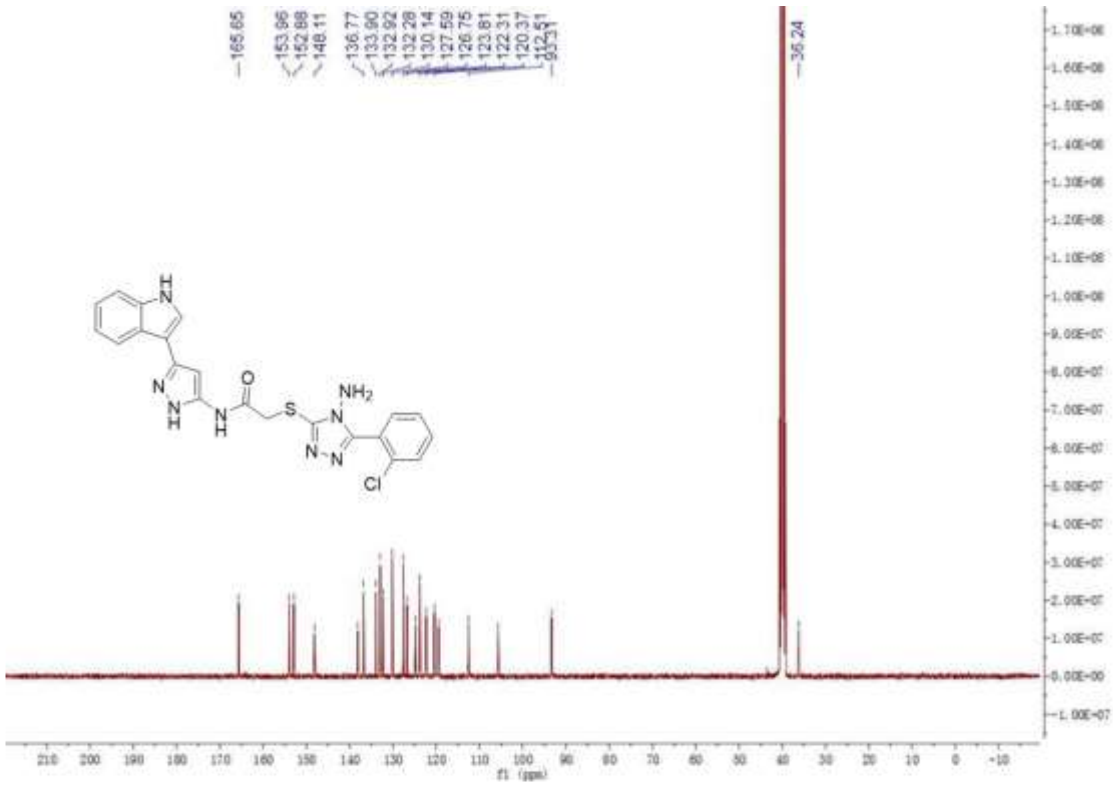

Figure S56. ^{13}**C NMR Spectrum (DMSO-**d_6**, 126 MHz) of 11j.**

Information Classification: General

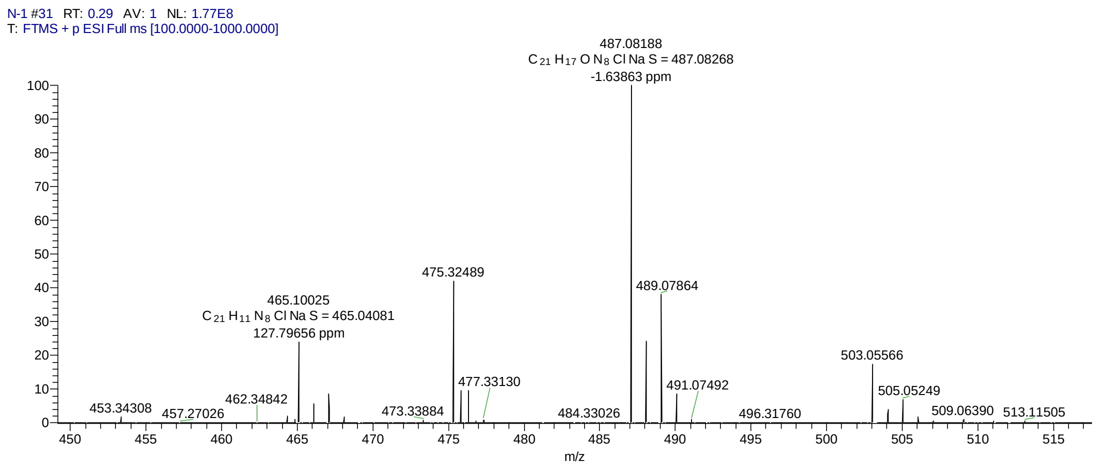

Figure S57. HR-MS Spectrum of 11j.

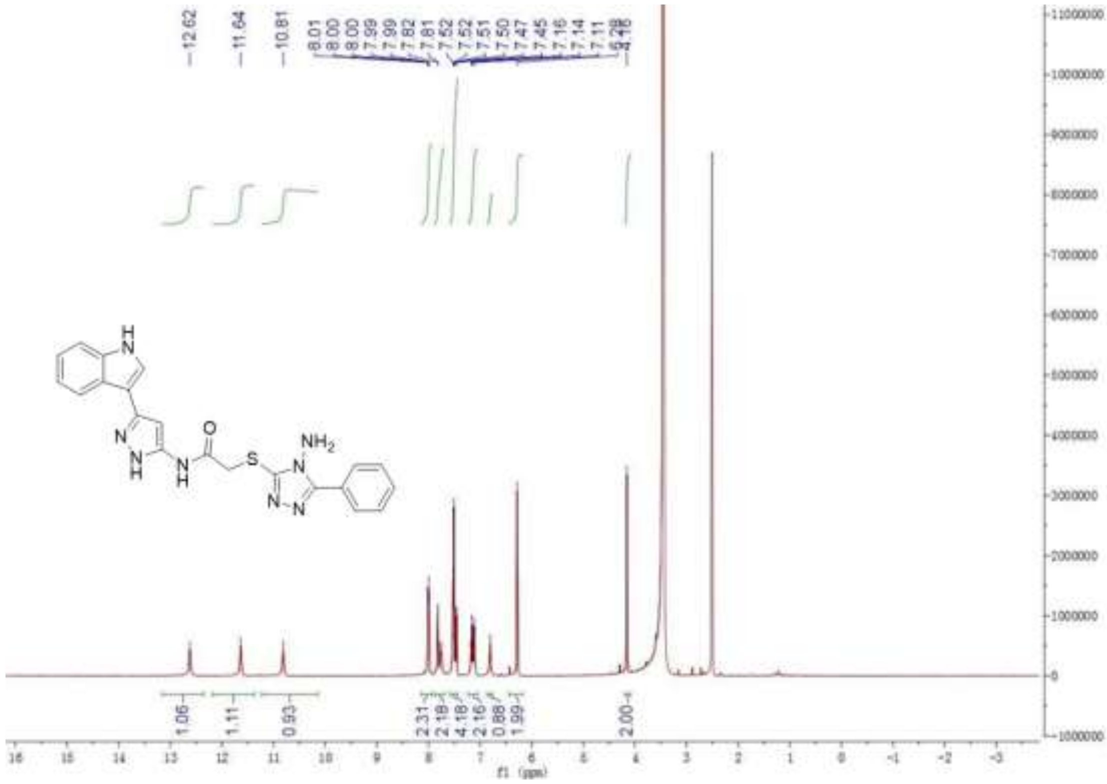

Figure S58. ^{1}H NMR Spectrum (DMSO-d_6, 500 MHz) of 11k.

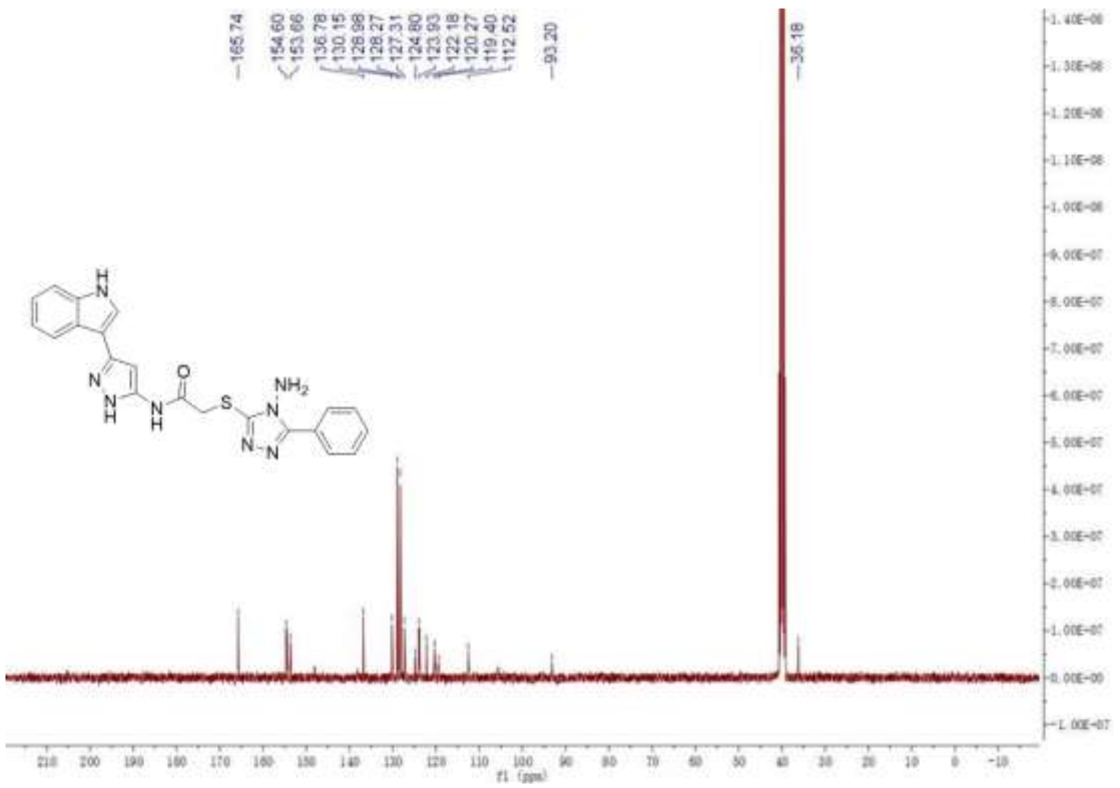

Figure S59. ^{13}C NMR Spectrum (DMSO-d_6, 126 MHz) of 11k.

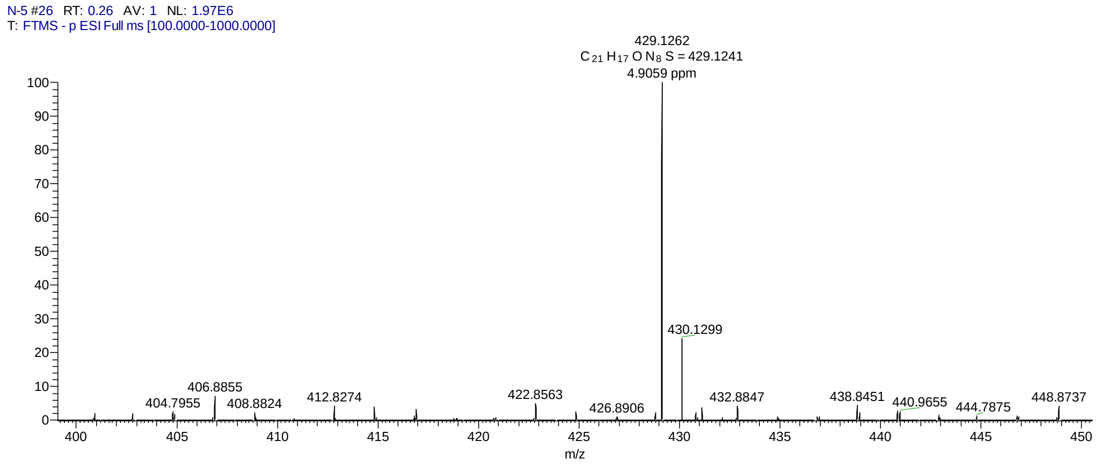

Figure S60. HR-MS Spectrum of 11k.

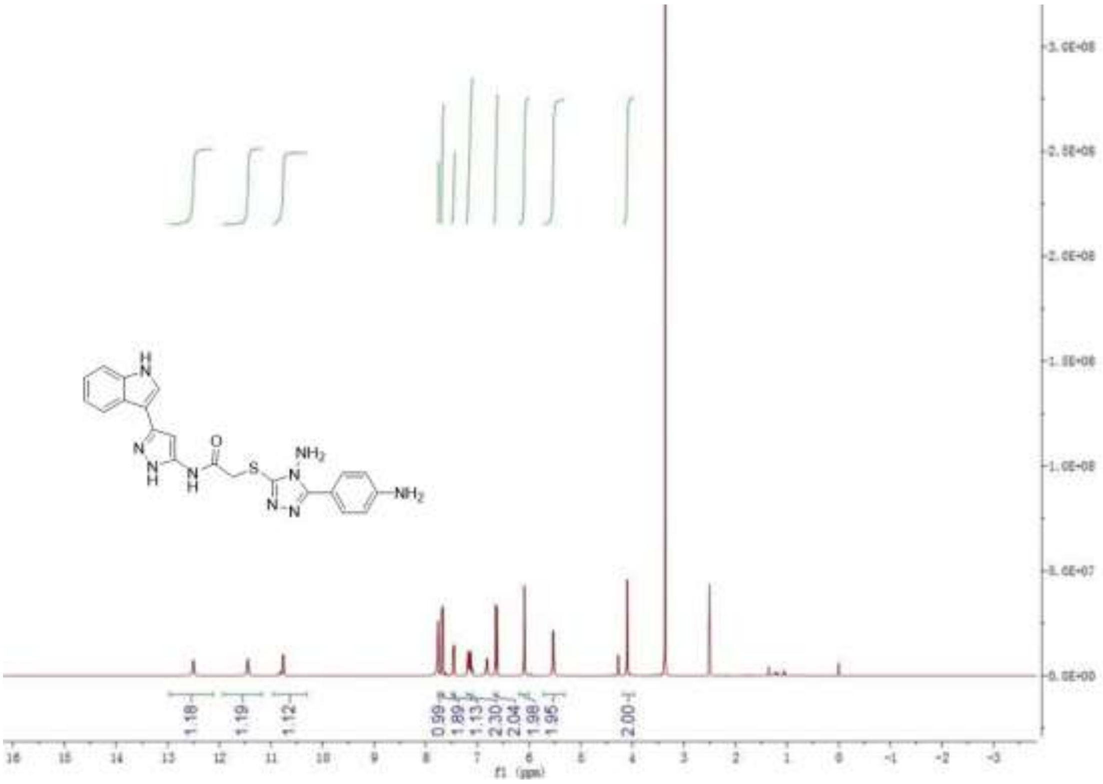

Figure S61. **^{1}H NMR Spectrum (DMSO-d_6, 500 MHz) of 11l.**

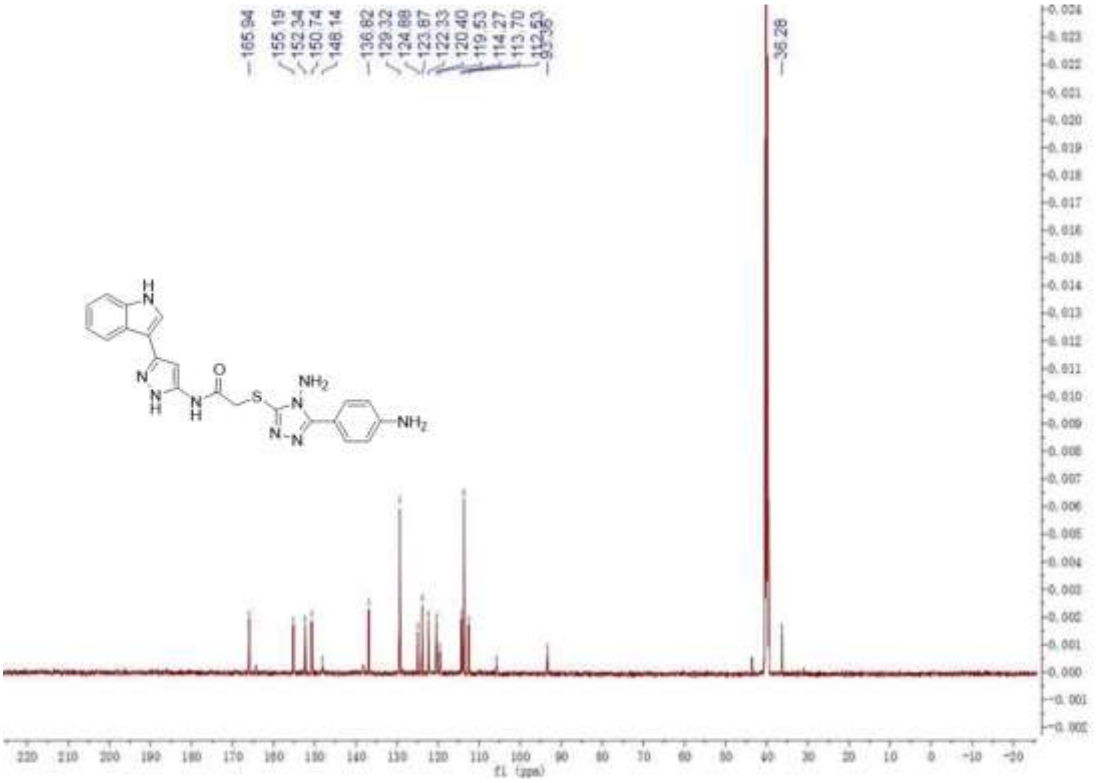

Figure S62. **^{13}C NMR Spectrum (DMSO-d_6, 101 MHz) of 11l.**

Information Classification: General

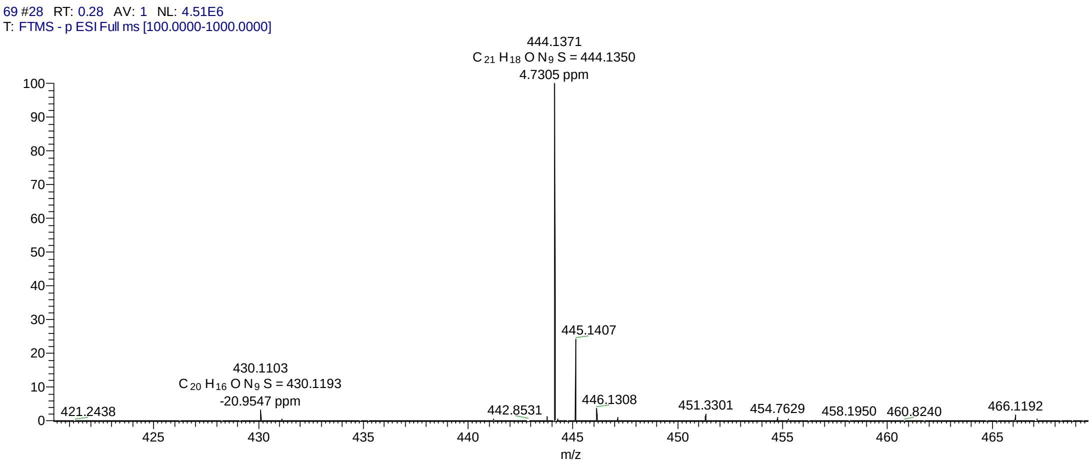

Figure S63. HR-MS Spectrum of 11l.

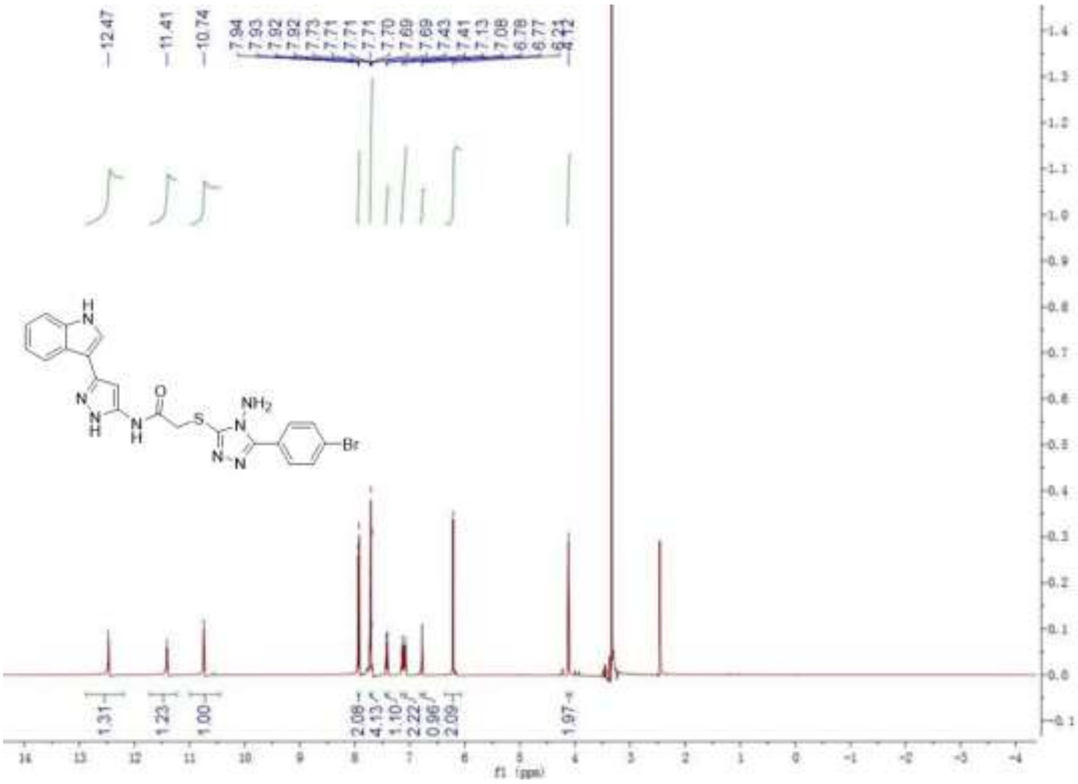

Figure S64. ^{1}H NMR Spectrum (DMSO-d_6, 500 MHz) of 11m.

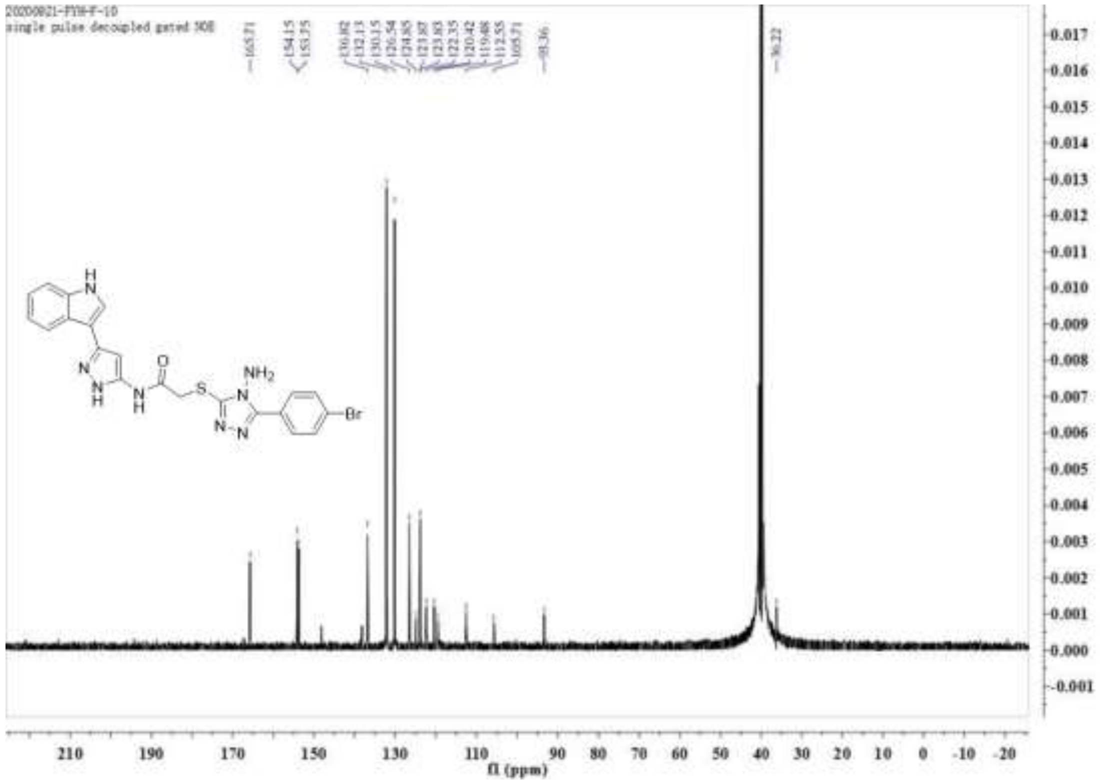

Figure S65. ^{13}**C NMR Spectrum (DMSO-*d*$_6$, 126 MHz) of 11m.**

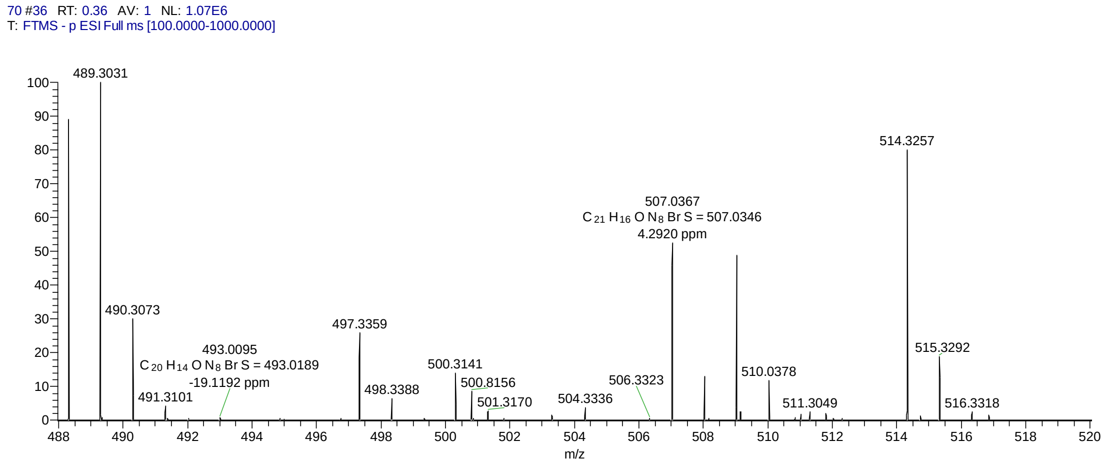

Figure S66. HR-MS Spectrum of 11m.

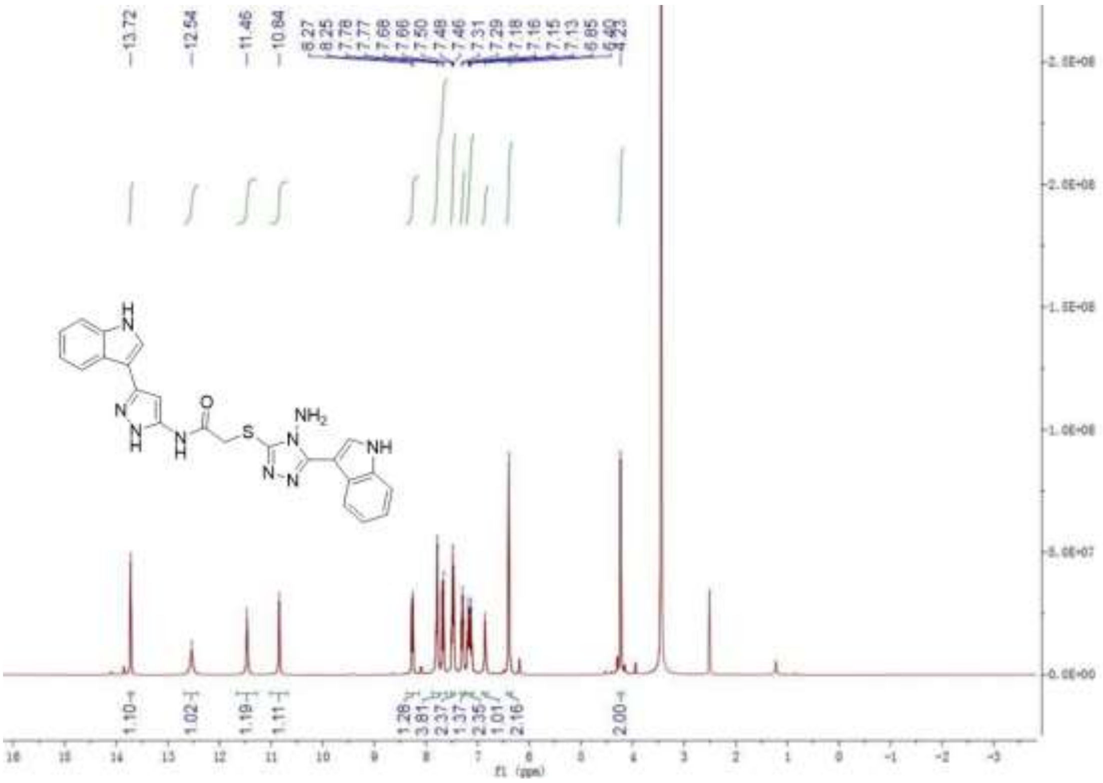

Figure S67. ^{1}**H NMR Spectrum (DMSO-*d*$_6$, 400 MHz) of 11n.**

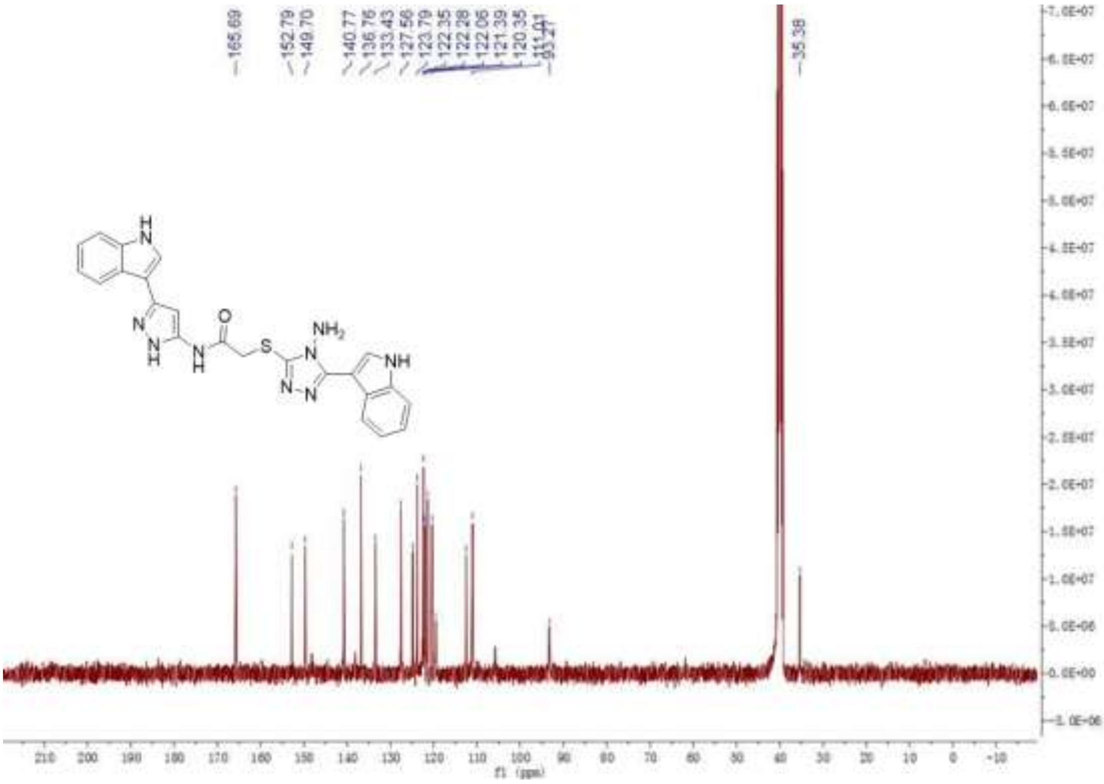

Figure S68. ^{13}**C NMR Spectrum (DMSO-*d*$_6$, 101 MHz) of 11n.**

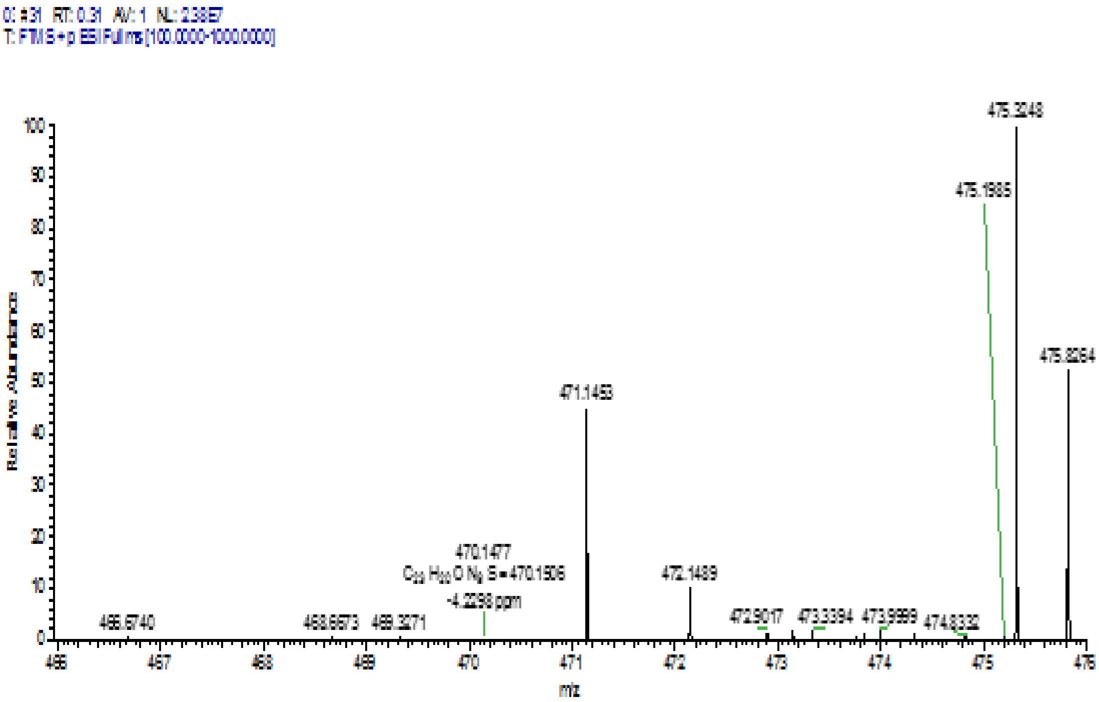

Figure S69. HR-MS Spectrum of 11n.

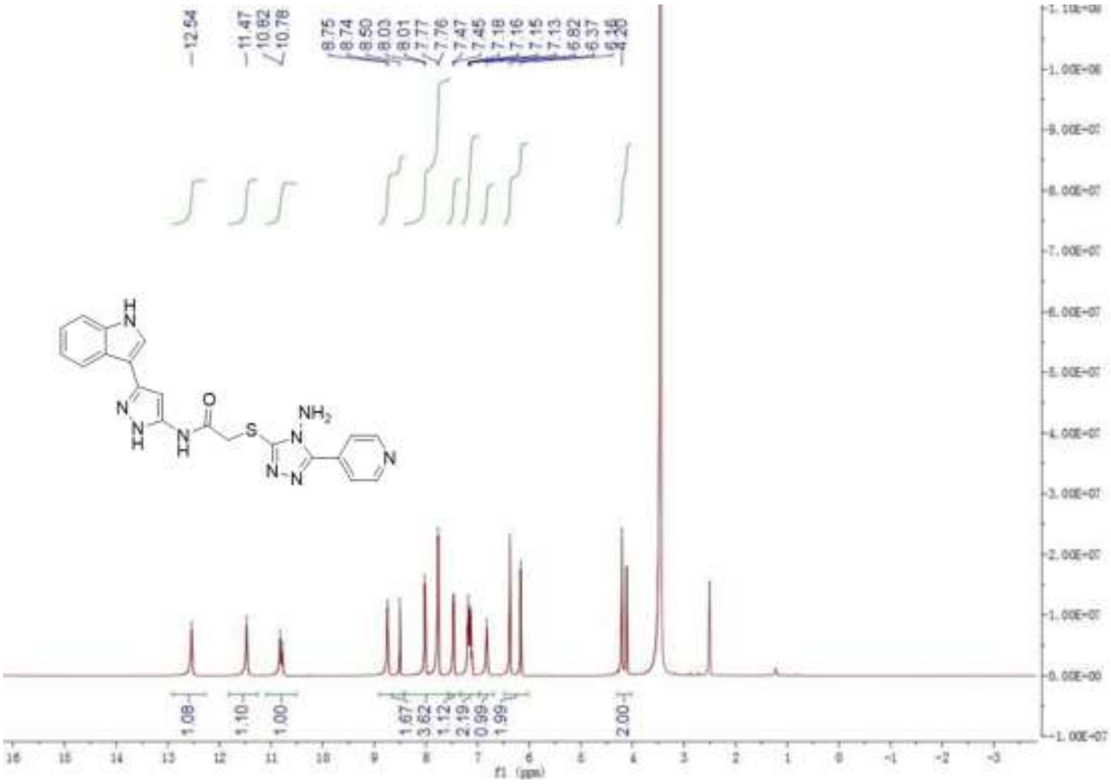

Figure S70. ^{1}H NMR Spectrum (DMSO-d_6, 400 MHz) of 11o.

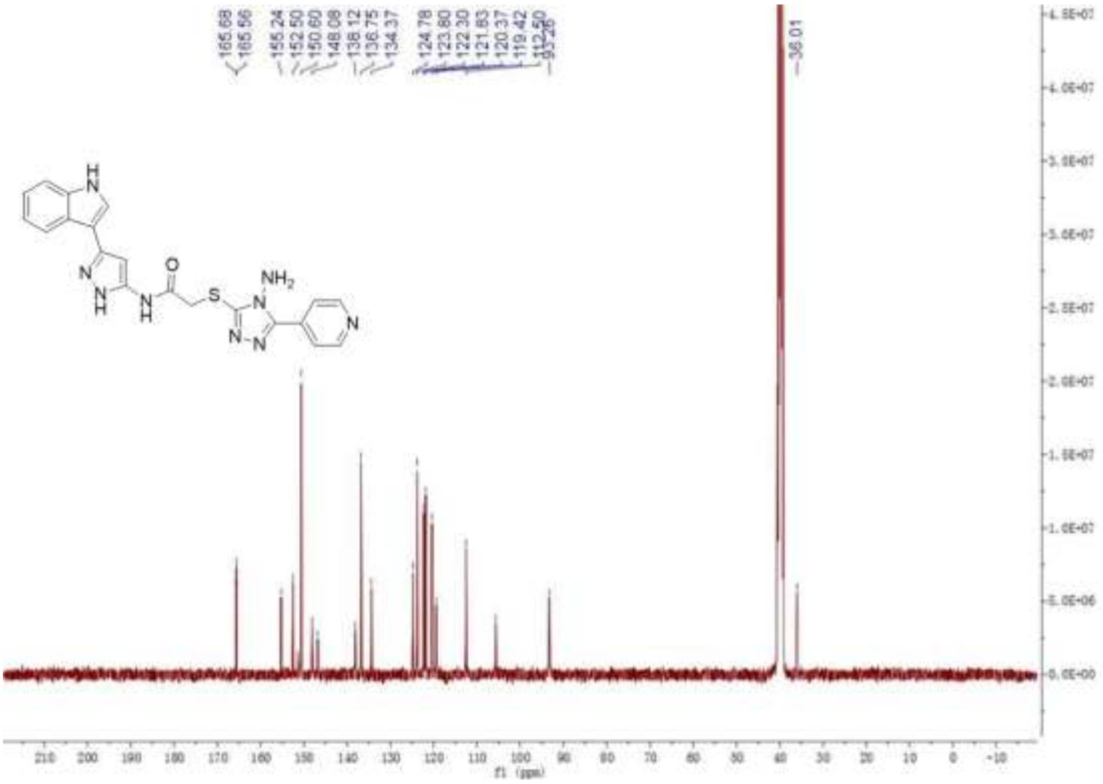

Figure S71. ^{13}**C NMR Spectrum (DMSO-*d*$_6$, 101 MHz) of 11o.**

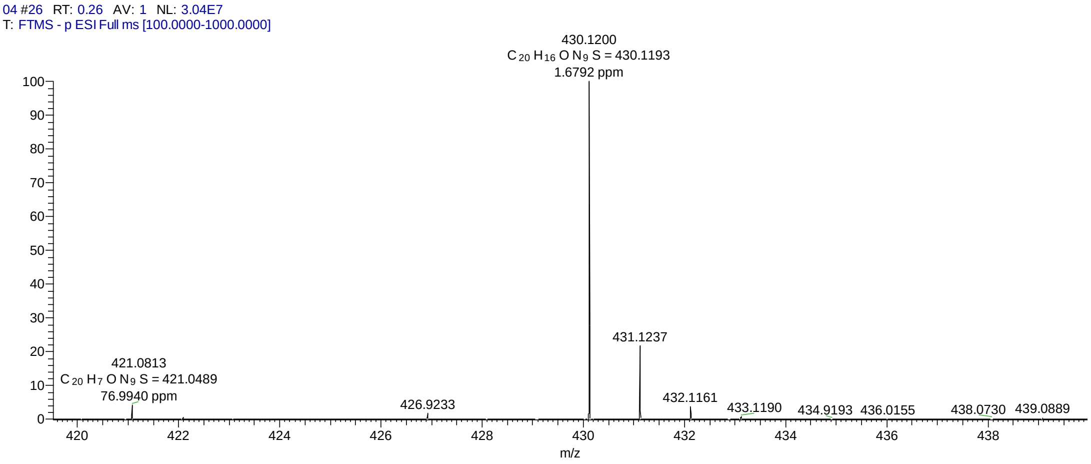

Figure S72. HR-MS Spectrum of 11o.

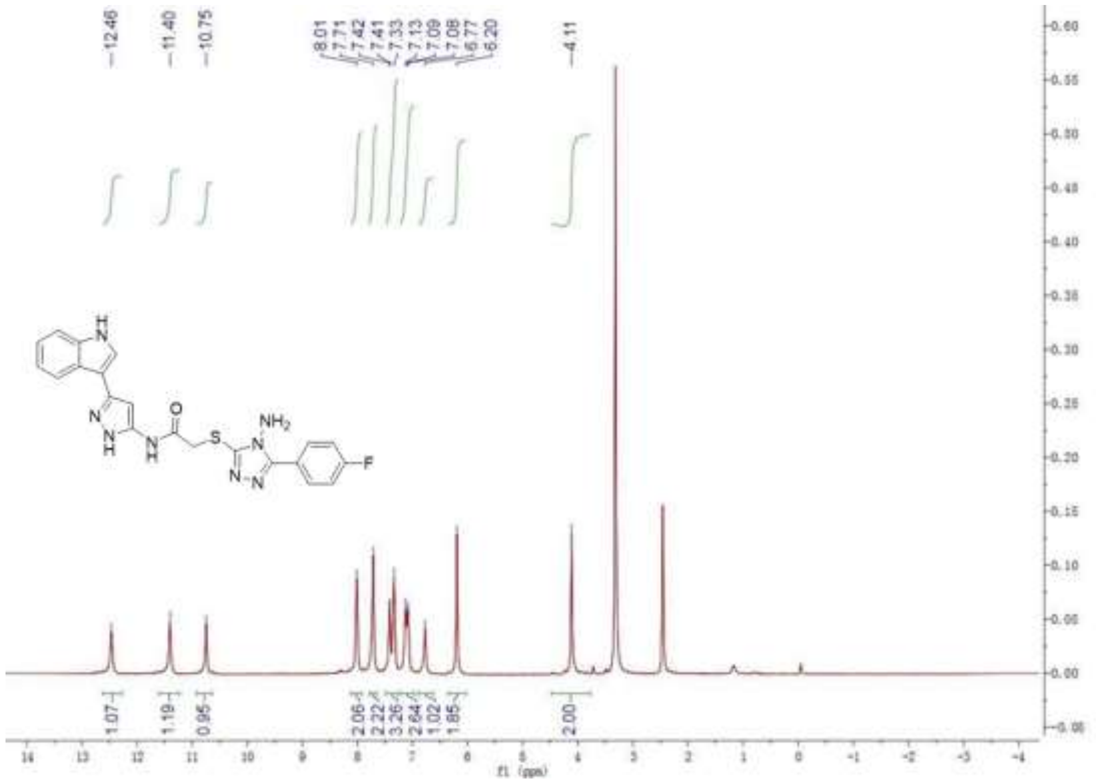

Figure S73. **^{1}H NMR Spectrum (DMSO-d_6, 500 MHz) of 11p.**

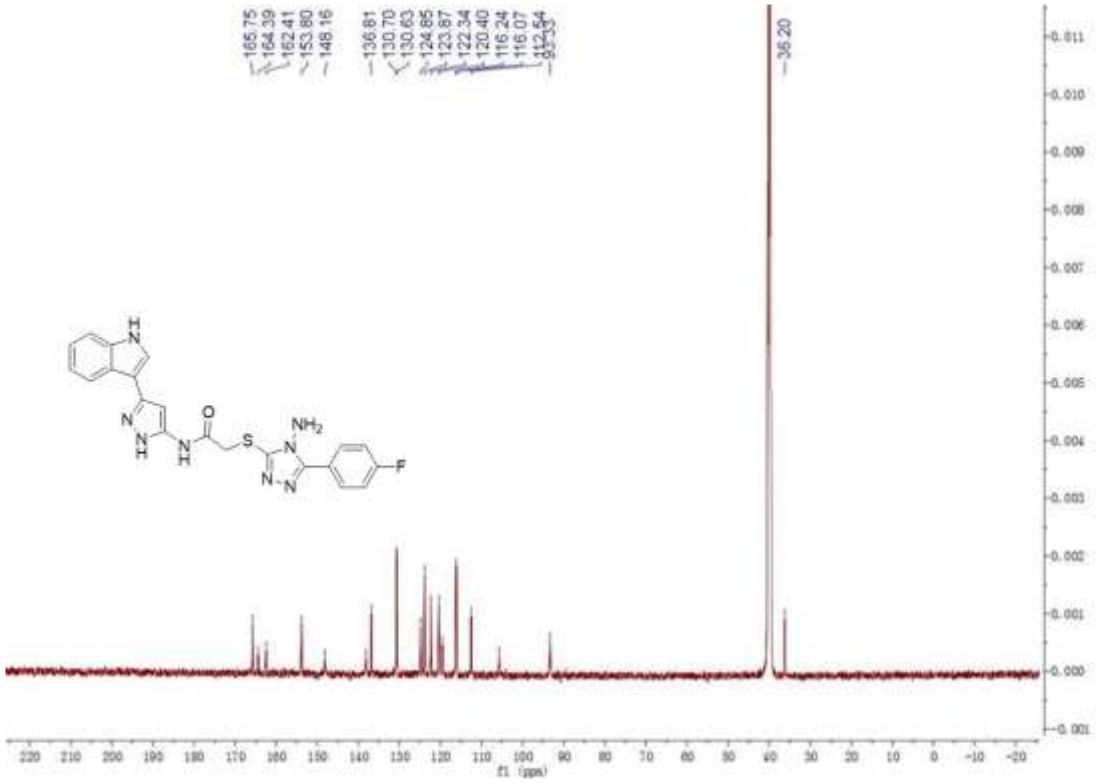

Figure S74. **^{13}C NMR Spectrum (DMSO-d_6, 126 MHz) of 11p.**

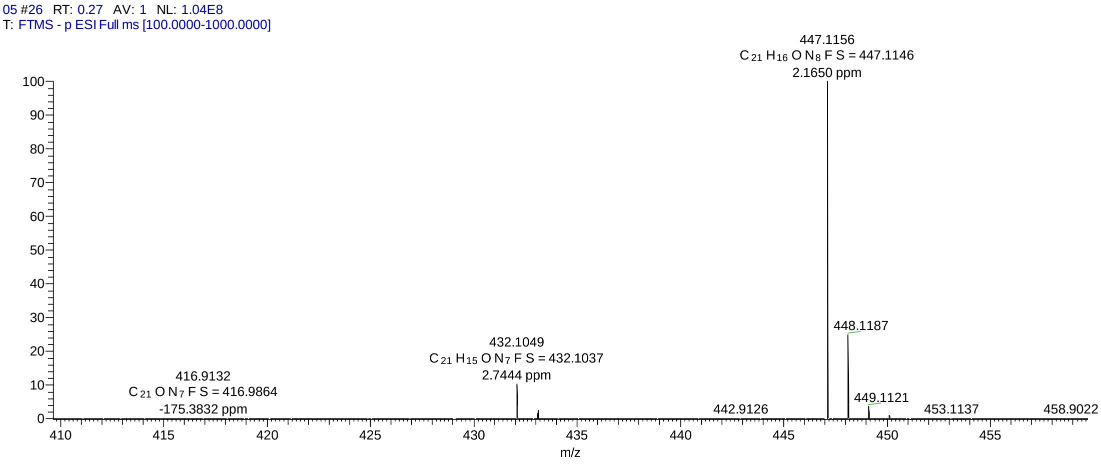

Figure S75. HR-MS Spectrum of 11p.

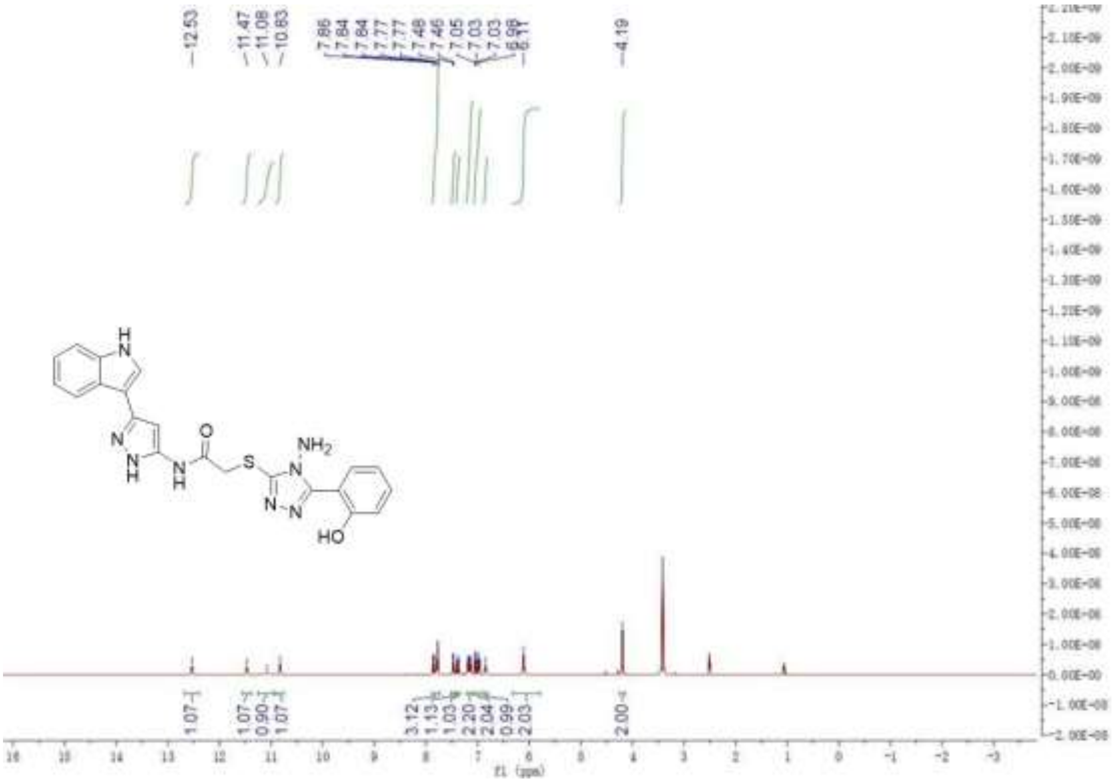

Figure S76. ^{1}H NMR Spectrum (DMSO-d_6, 500 MHz) of 11q.

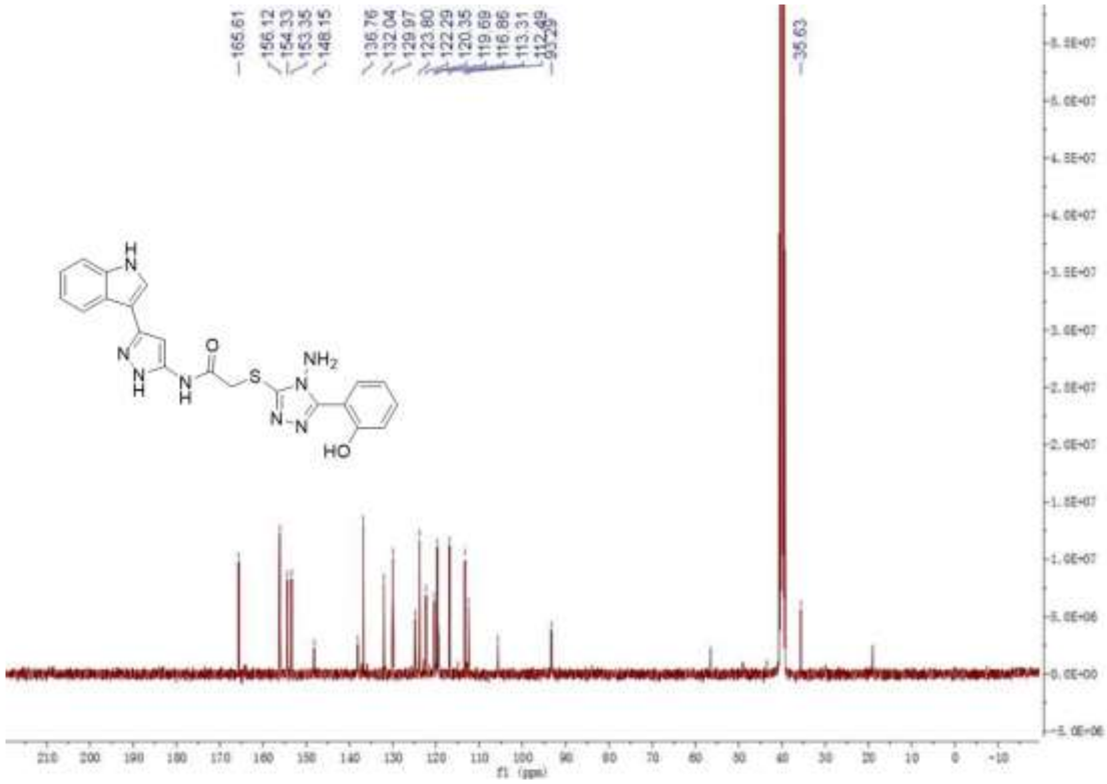

Figure S77. ^{13}C NMR Spectrum (DMSO-d6, 126 MHz) of 11q.

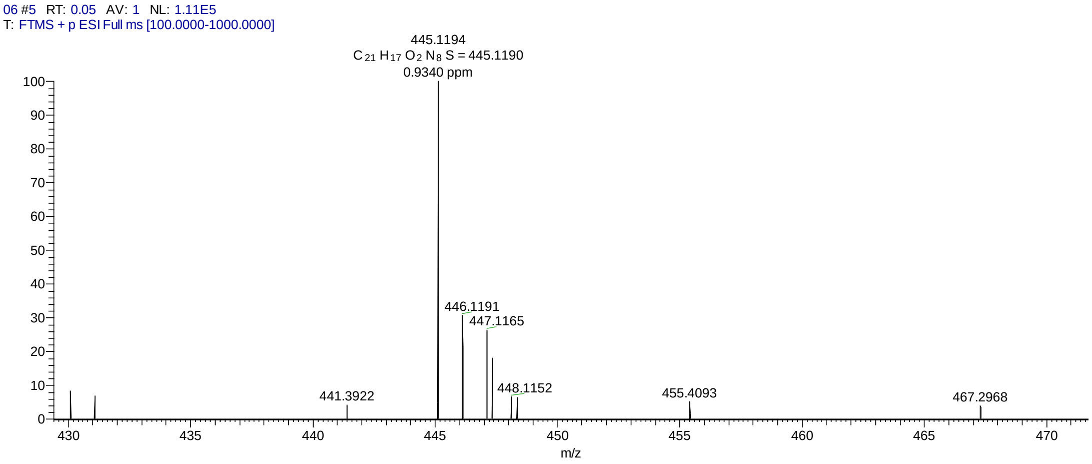

Figure S78. HR-MS Spectrum of 11q.

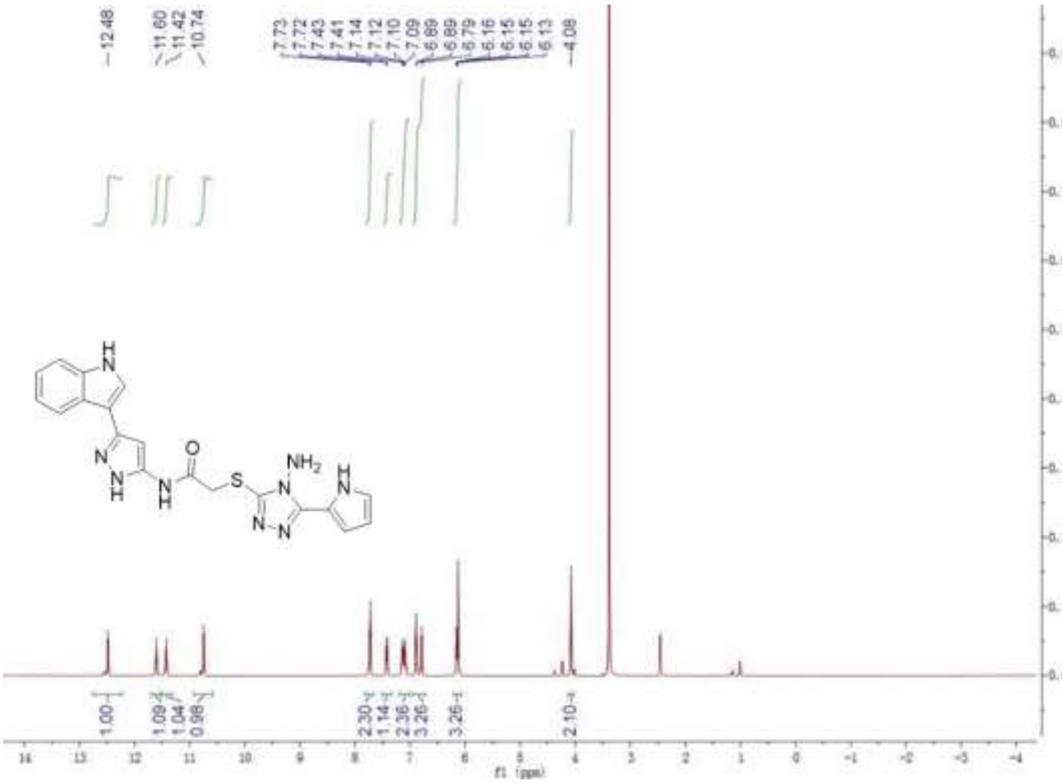

Figure S79. ^{1}H NMR Spectrum (DMSO-d6, 500 MHz) of 11r.

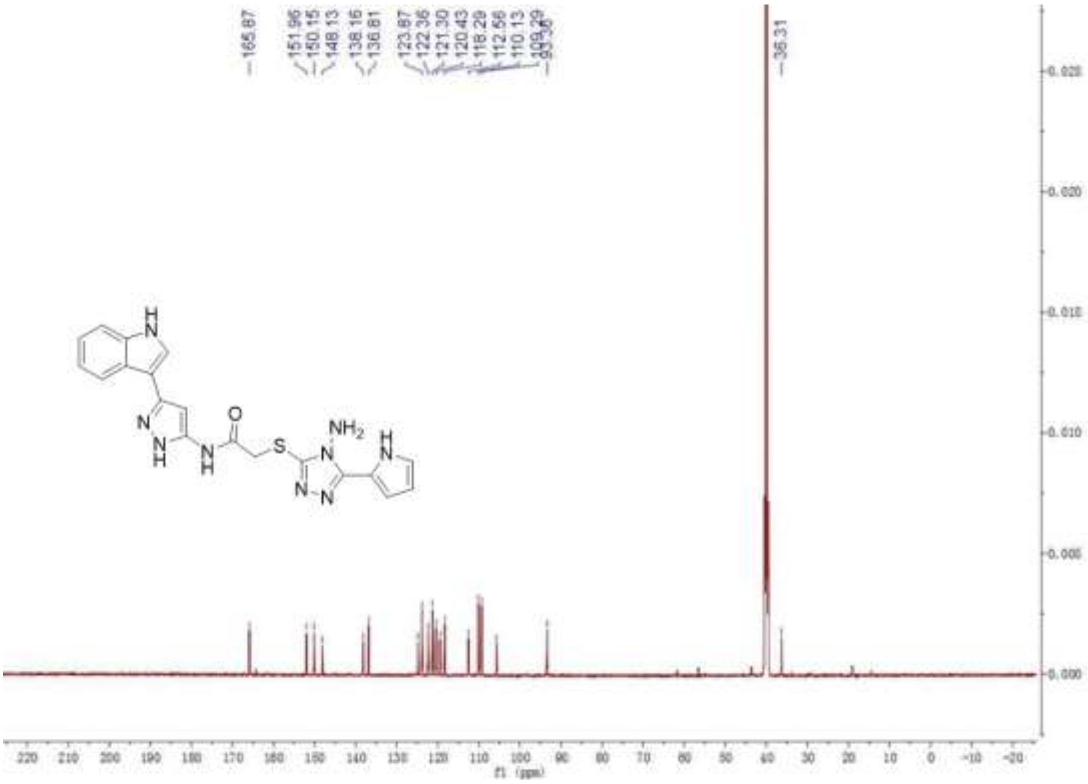

Figure S80. ^{13}C NMR Spectrum (DMSO-d6, 126 MHz) of 11r.

Information Classification: General

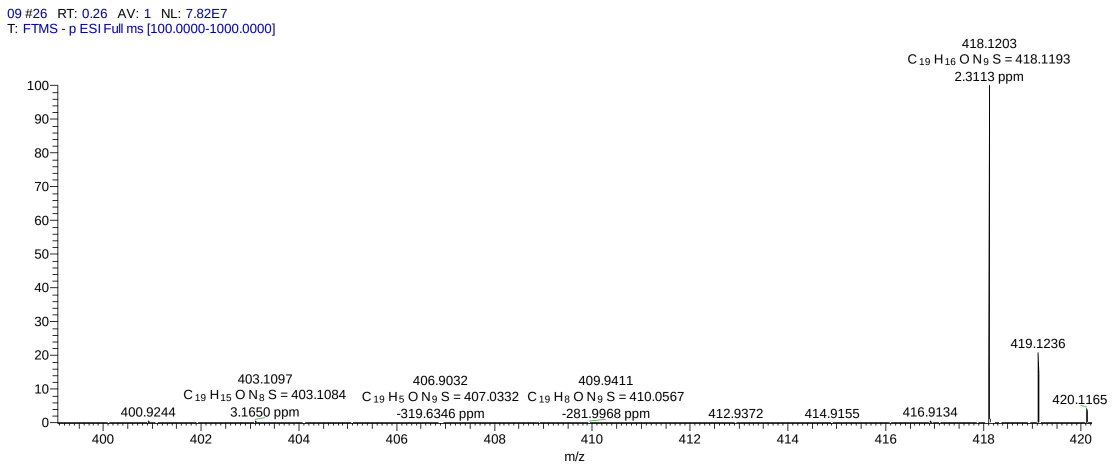

Figure S81. HR-MS Spectrum of 11r.

2. ^{1}H NMR and ^{13}C NMR spectra for key intermediate 4.

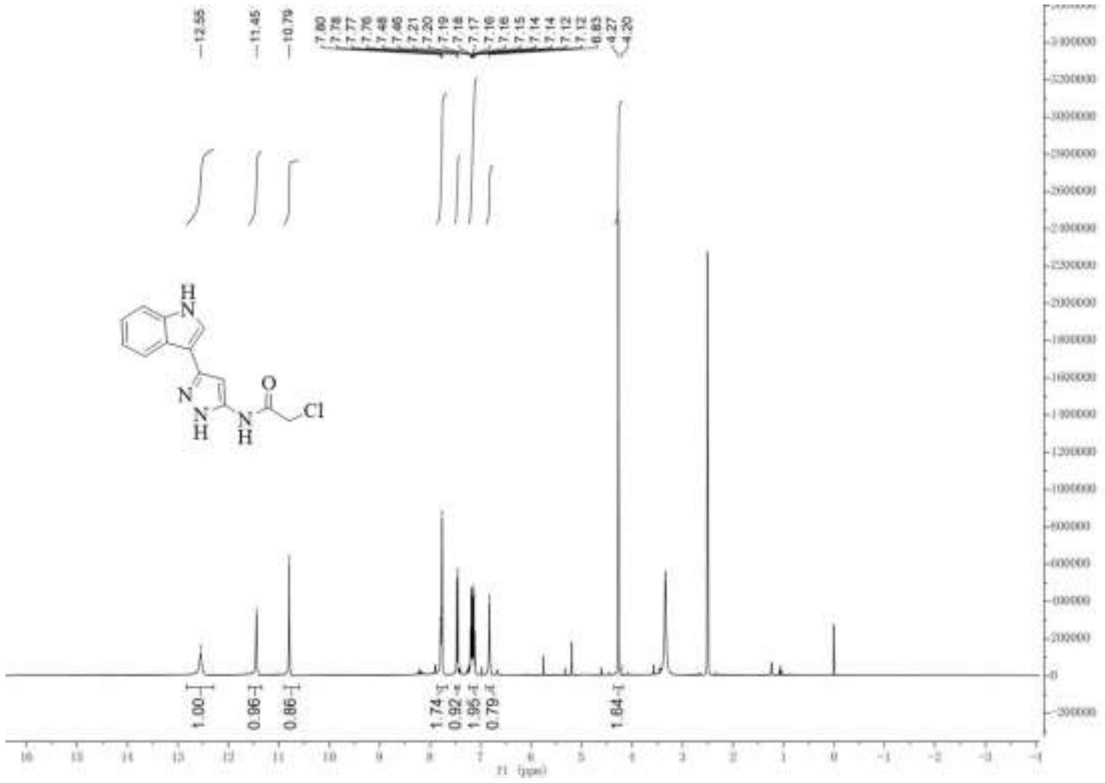

Figure S82. ^{1}H NMR Spectrum (DMSO-d6, 500 MHz) of key intermediate 4.

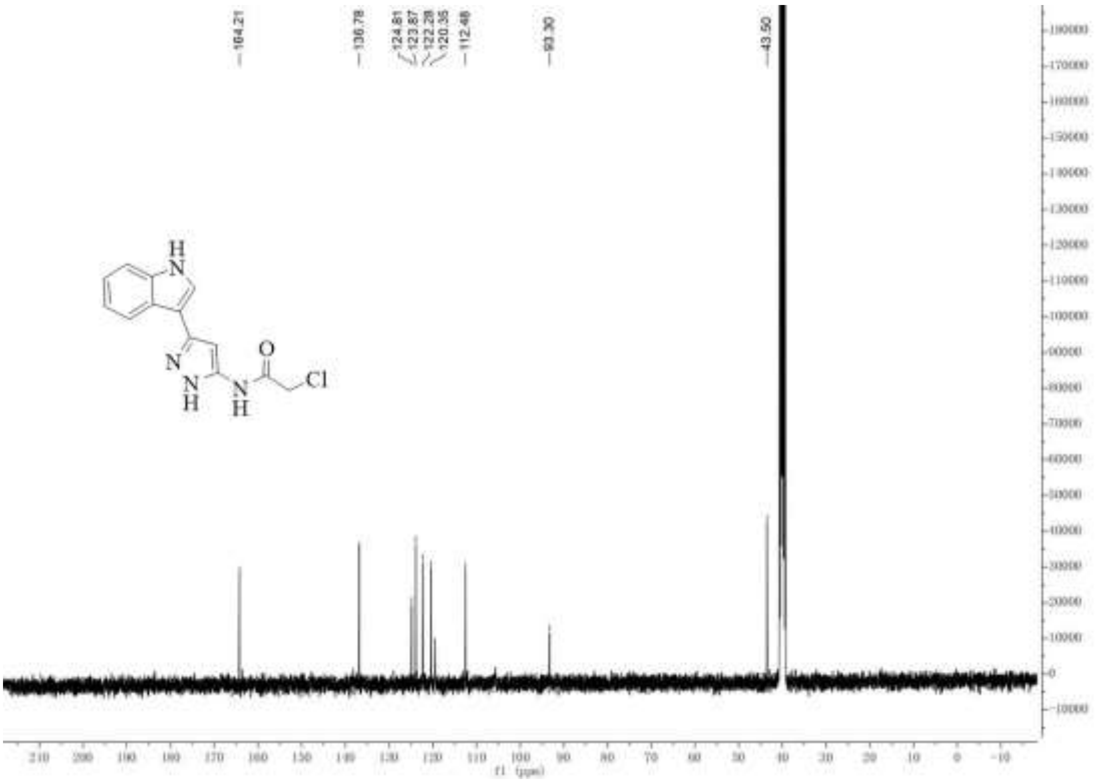

Figure S83. ^{13}C NMR Spectrum (DMSO-d6, 126 MHz) of key intermediate 4.

Information Classification: General

3. Superimposition of the docked ligand of gefitinib with 10b and 11h, respectively.

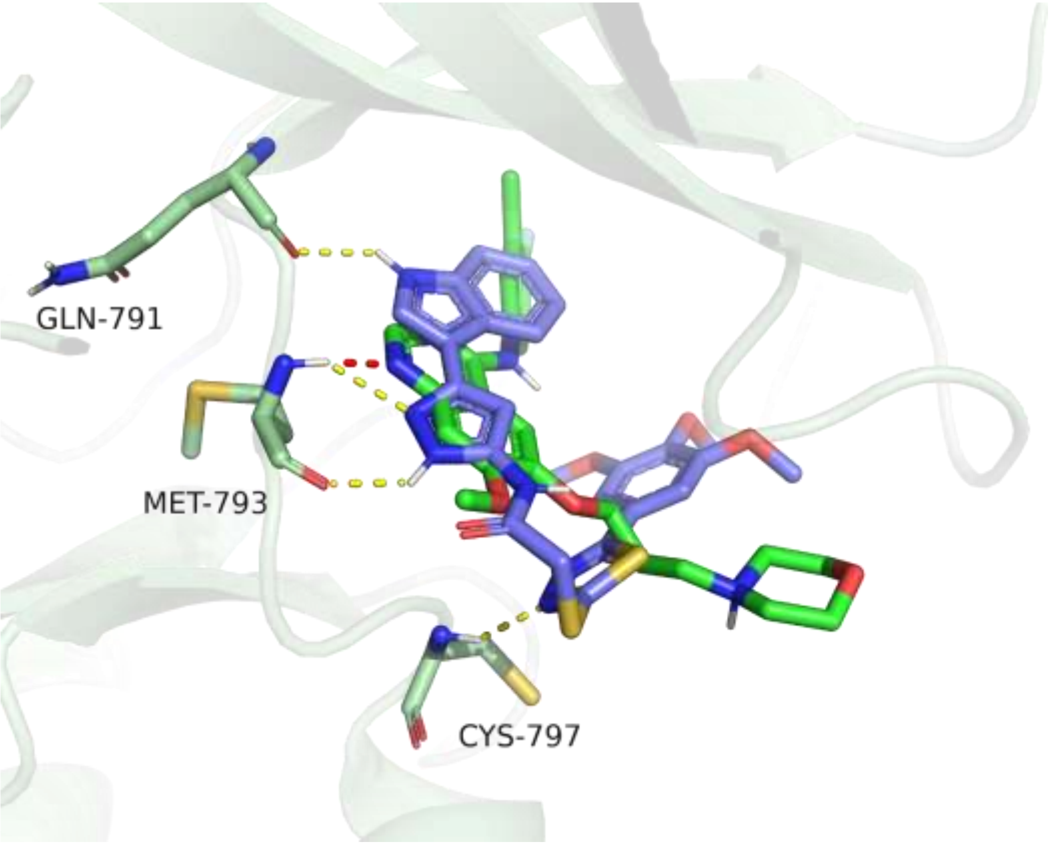

Figure S84. Superimposition of gefitinib with 10b.

Information Classification: General

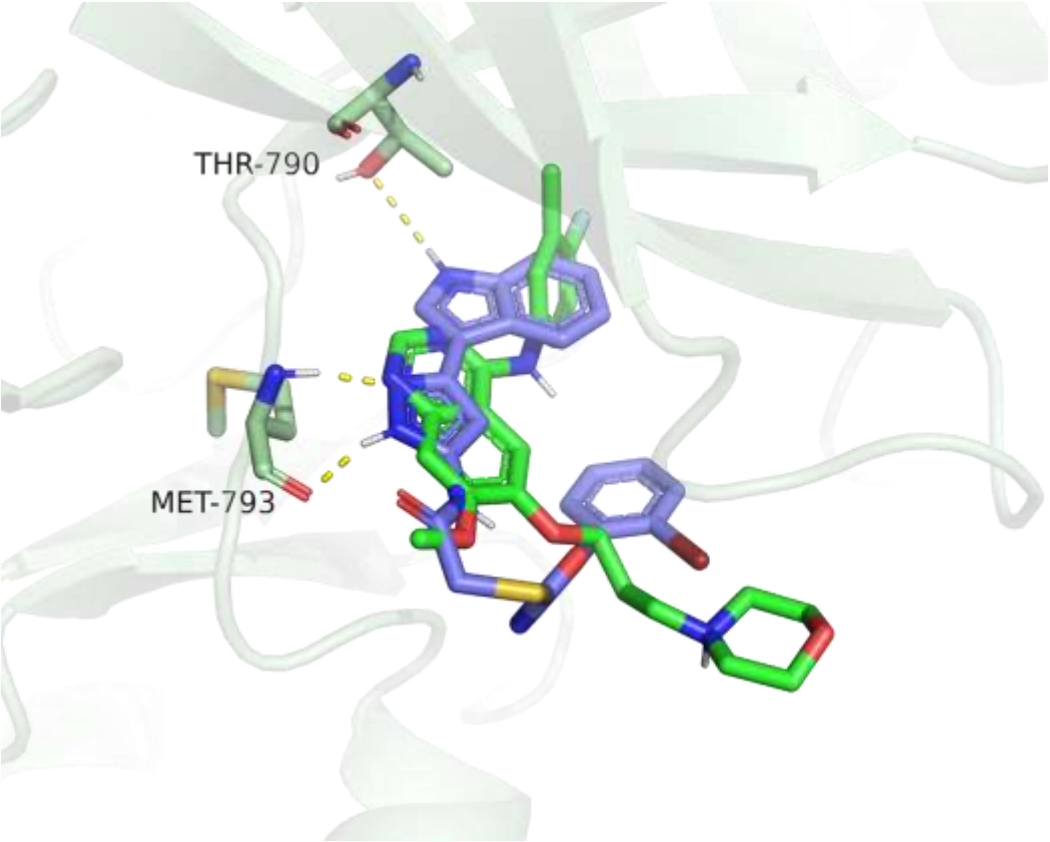

Figure S85. Superimposition of gefitinib with 11h.

4. Table S1. *In vitro* inhibitory activities of target compounds for human cancer cell lines A549, PC-3，HepG2, K562 and HEK293.

Table S1. *In vitro* inhibitory activities of target compounds for human cancer cell lines A549, PC-3，HepG2, K562 and HEK293.

Compounds	Inhibition rate (%, 10 μM)				
	A549	PC - 3	HepG2	K562	HEK293
**10a**	85.6 ± 3.54	95.7 ± 0.624	76.0 ± 3.39	96.0 ± 2.31	87.6 ± 2.19
**10b**	96.2 ± 0.737	88.4 ± 5.99	92.1 ± 3.01	97.2 ± 0.526	77.9 ± 2.08
**10c**	24.3 ± 2.77	30.3 ± 12.6	11.9 ± 2.21	27.1 ± 1.87	33.0 ± 1.46
**10d**	90.7 ± 6.19	82.6 ± 4.95	93.7 ± 1.88	96.1 ± 1.29	84.7 ± 2.75
**10e**	92.2 ± 1.01	92.5 ± 0.391	95.5 ± 0.354	98.7 ± 1.05	85.5 ± 1.26
**10f**	85.2 ± 3.63	58.7 ± 28.6	90.3 ± 2.77	95.8 ± 3.98	60.9 ± 6.65
**10g**	55.2 ± 3.17	96.4 ± 2.21	56.9 ± 9.69	97.4 ± 0.838	68.1 ± 4.49

Information Classification: General

**10h**	43.5 ± 1.70	28.9 ± 2.71	63.2 ± 5.32	64.3 ± 8.28	18.7 ± 8.52
**10i**	35.7 ± 2.75	30.5 ± 9.10	51.9 ± 5.57	40.5 ± 6.54	46.5 ± 7.50
**11a**	32.9 ± 3.84	79.0 ± 3.97	19.3 ± 4.13	95.6 ± 1.68	58.5 ± 2.72
**11b**	32.6 ± 3.42	80.3 ± 3.56	72.7 ± 4.94	96.4 ± 1.16	72.1 ± 1.62
**11c**	10.5 ± 0.521	12.3 ± 2.04	10.4 ± 1.73	14.9 ± 1.32	1.73 ± 2.31
**11d**	87.4 ± 12.5	56.2 ± 9.24	16.3 ± 2.10	95.4 ± 0.809	63.4 ± 7.49
**11e**	47.9 ± 9.44	54.7 ± 14.8	63.5 ± 20.0	98.7 ± 0.418	67.3 ± 1.39
**11f**	32.8 ± 4.22	28.9 ± 3.01	46.9 ± 6.62	25.0 ± 3.24	40.1 ± 2.19
**11g**	67.5 ± 1.65	72.2 ± 13.2	74.9 ± 25.5	98.5 ± 0.653	65.4 ± 6.38
**11h**	97.8 ± 0.354	65.1 ± 0.595	93.6 ± 0.215	98.6 ± 0.417	77.8 ± 4.16
**11i**	94.8 ± 1.91	83.6 ± 5.66	95.3 ± 3.04	99.5 ± 0.155	67.1 ± 2.18
**11j**	13.7 ± 1.49	17.8 ± 2.85	14.4 ± 0.686	14.3 ± 0.525	10.9 ± 7.59
**11k**	23.2 ± 0.539	23.1 ± 3.99	11.1 ± 0.757	19.2 ± 3.55	0.497 ± 5.03
**11l**	96.7 ± 0.847	71.9 ± 4.55	93.5 ± 1.56	99.0 ± 0.200	79.6 ± 2.23
**11m**	71.7 ± 5.61	32.8 ± 6.50	60.9 ± 7.02	98.5 ± 0.434	54.2 ± 5.97
**11n**	31.0 ± 0.996	56.6 ± 12.9	80.5 ± 3.62	97.1 ± 0.377	44.7 ± 4.73
**11p**	23.9 ± 3.03	17.1 ± 1.22	22.8 ± 2.22	11.7 ± 0.639	5.33 ± 14.9
**11q**	89.4 ± 2.21	86.3 ± 0.332	84.7 ± 11.4	99.2 ± 1.20	99.3 ± 0.0200
**11r**	94.2 ± 1.81	79.4 ± 9.21	96.5 ± 1.22	95.0 ± 2.52	72.3 ± 5.76
**5F**	54.3 ± 1.07	37.0 ± 6.18	26.4 ± 0.513	39.9 ± 6.44	72.1 ± 3.71

Information Classification: General
